# Synthesis of novel derivatives of 5-hydroxymethylcytosine and 5-formylcytosine as tools for epigenetics

**DOI:** 10.3762/bjoc.10.2

**Published:** 2014-01-03

**Authors:** Anna Chentsova, Era Kapourani, Athanassios Giannis

**Affiliations:** 1 Institut für Organische Chemie, Fakultät für Chemie und Mineralogie, Universität Leipzig, Johannisallee 29, 04103 Leipzig, Germany

**Keywords:** 3,6-dihydrodeoxycytidine derivatives, DNA demethylation, epigenetics, 5-hydroxymethylcytosine (5hmC) derivatives, TET-enzymes

## Abstract

In this work we present for the first time the synthesis of novel 5-hydroxymethylcytosine (5hmC) and 5-formylcytosine (5fC) derivatives that can be used as tools in the emerging field of epigenetics for deciphering chemical biology of TET-mediated processes.

## Introduction

**Scheme 1:**
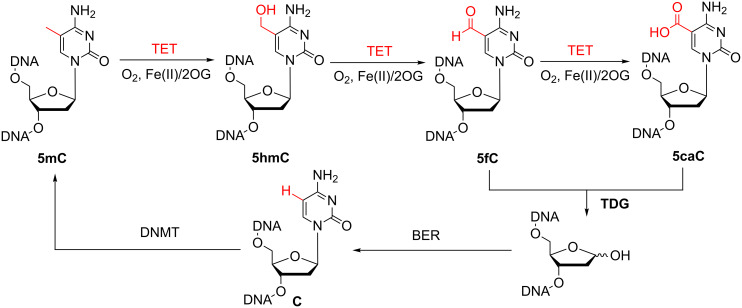
Proposed steps for DNA demethylation (for details see text).

Epigenetic modifications play a crucial role in cell differentiation and cell development [1]. They control gene expression through several mechanisms such as non-coding RNAs, histone modifications (acetylation, methylation, phosphorylation, etc.) [2], and DNA methylation [3–7]. The latter takes place at the C-5 position of the cytosine moiety in CpG islands establishing the so called 5^th^ base: 5-methylcytosine (5mC), a well-known epigenetic mark that correlates with gene silencing [8]. Recently, conversion of the 5mC moiety to 5-hydroxymethylcytosine (5hmC) and to higher oxidation products such as 5-formylcytosine (5fC) and 5-carboxylcytosine (5caC) by the action of ten-eleven-translocation (TET) enzymes was discovered [9–13]. The TET proteins are identified as 2-oxoglutarate (2OG) and Fe(II)-dependent oxygenases [10,14]. Whereas the DNA methylation is a densely studied field, its reverse process has not yet been deciphered. In trying to understand DNA demethylation several mechanisms involving new cytosine-modified bases as intermediates have been proposed (Scheme 1). (1) The most widely accepted pathway includes iterative oxidation of 5mC catalyzed by TET enzymes followed by removal of 5fC and 5caC by thymine DNA glycosylase (TDG). Excision of 5fC and 5caC generates an abasic site, which is further repaired resulting in replacement of 5mC with unmodified cytosine (C) [15–18]. (2) The second alternative scenario still remains controversial [19–20]. It links the oxidative action of TET enzymes, the subsequent deamination of 5hmC to 5-hydroxymethyluracil (5hmU) by cytidine deaminases AID (activation-induced cytidine deaminase) or APOBEC (apolipoprotein B mRNA editing enzyme, catalytic polypeptide) with the base excision repair (BER) machinery [15–17,21]. (3) Among other putative demethylation mechanisms is the direct dehydroxymethylation of 5hmC to cytosine by action of DNA methyltransferases (DNMT). This enzymatic process was observed in vitro, whether it also works in vivo is yet to be elucidated [15,22]. (4) Lastly, decarboxylation of the 5caC by an unknown decarboxylase excluding action of BER should also be considered [15,23]. This variety of demethylation pathways might indicate that different tissues utilize different demethylation pathways [1,24].

While DNA methylation is usually associated with gene repression [8,25], active demethylation seems to allow cells to unblock silenced genes aiming at epigenetic reprogramming of their genetic material [26]. Current accepted models propose that 5hmC could be involved in epigenetic modulation of gene activity. In fact, 5hmC was discovered also in embryonic stem cells and seems to play a decisive role in their self-renewal process [27]. Interestingly, the levels of 5hmC in several cancer types are strongly reduced relative to the corresponding normal tissue around the tumor [28]. To gain deeper insights into the chemical biology of DNA demethylation pathways further exploration of the TET-mediated processes is necessary. Analogues of 5hmC with substituents preventing formation of 5fC and 5caC species could serve as useful tools for ongoing investigations in this emerging field.

## Results and Discussion

**Figure 1:**
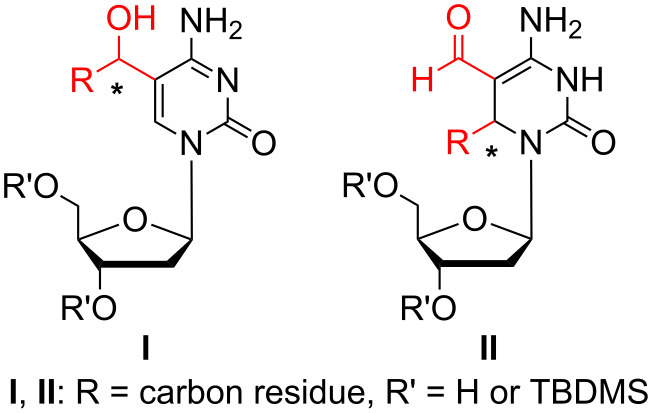
Structures of the synthesized compounds.

Herein, we describe the synthesis of compounds with the general formula **I** which represents modified cytidine analogues bearing a secondary alcohol at position C-5 of cytosine. Additionally, a synthesis of 3,6-dihydrodeoxycytidine derivatives of general formula **II** is presented (Figure 1).

**Scheme 2:**
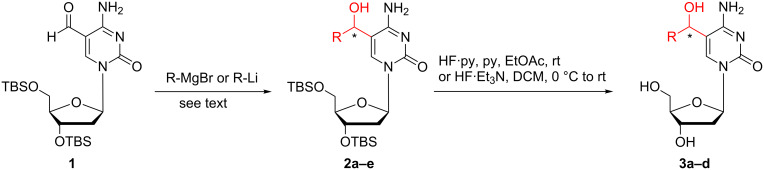
Synthesis of the 2'-deoxycytidine analogues.

We chose the known aldehyde **1** [29] (prepared from commercially available 2’-deoxycytidine) as a starting material for the envisioned transformations (Scheme 2). To the best of our knowledge, the addition of organometallic compounds (organolithium and organomagnesium, etc.) to aldehyde **1** is not described in the literature. Compound **1** was readily converted to 5hmC analogues **2a**–**e** by treatment with various Grignard reagents (methylmagnesium bromide, THF, 0 °C → room temperature, or vinylmagnesium bromide, THF, 0 °C → room temperature) and organolithium reagents (lithium (trimethylsilyl)acetylide, THF, −40 °C → −20 °C or lithium phenylacetylide, THF, −78 °C → −50 °C) (Scheme 2). These alcohols were obtained as a mixture of diastereomers in yields ranging from 43% to 96% (Table 1). Compound **2b** was isolated in moderate yield of 43% due to the cleavage of the TMS-group during the reaction resulting in formation of derivative **2e** with a yield of 26%. The obtained derivatives **2a**–**d** were further treated with Olah’s reagent and pyridine in EtOAc at room temperature or HF·triethylamine complex [30] in DCM at 0 °C to afford the deprotected 2’-deoxycytidine analogues **3a**–**d** as mixtures of diastereomers in yields of 60–75%.

**Table 1:** Yields and ratios of diastereomeric alcohols **2a**–**e** and **3a**–**d**.

Entry	**2**/**3**	Yield [%]	Ratio^a^

1	**2a**, R = methyl	96	1.1:1
2	**2b**, R = (TMS)ethynyl	43	1.9:1
3	**2c**, R = phenylethynyl	68	1.2:1
4	**2d**, R = vinyl	77	1.1:1
5	**2e**, R = ethynyl	26	1.2:1
6	**3a**, R = methyl	75	n.d.
7	**3b,** R = ethynyl	60	n.d.
8	**3c**, R = phenylethynyl	72	1.1:1
9	**3d**, R = vinyl	73	n.d.

^a^Determined by ^1^H NMR; n.d. = not determined.

**Scheme 3:**
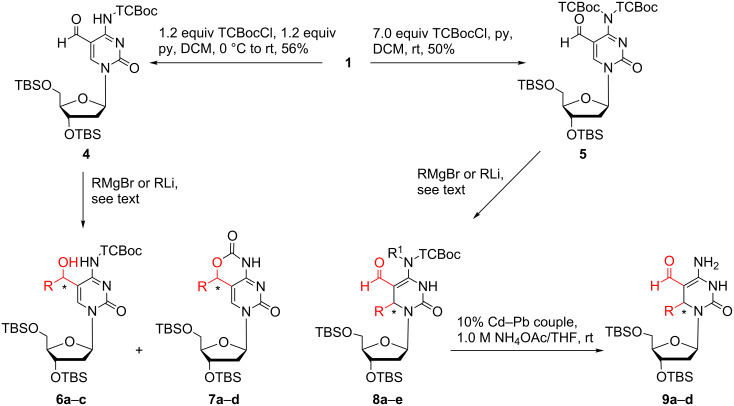
Reactions of TCBoc-protected aldehydes **4** and **5** with organometallic reagents.

Next, we synthesized the *N*-4-protected cytidine derivatives **4** and **5** by treatment of aldehyde **1** with β,β,β-trichloro-*tert*-butoxycarbonyl chloride (TCBocCl) [31] in the presence of pyridine in DCM (Scheme 3). The reaction of **4** with Grignard (methylmagnesium bromide, THF, 0 °C → room temperature, or vinylmagnesium bromide, THF, 0 °C → room temperature) and organolithium reagents (lithium (trimethylsilyl)acetylide, THF, −60 °C → −50 °C or lithium phenylacetylide, THF, −78 °C) afforded derivatives **6a**–**c** and carbamates **7a**–**c** as mixtures of diastereomers (Table 2). It should be mentioned that upon storage at room temperature derivatives **6a**–**c** undergo slow intramolecular cyclization to the corresponding carbamates **7a**–**c**. The reaction of aldehyde **4** and vinylmagnesium bromide yielded directly carbamate **7d**.

**Table 2:** Yields and ratios of diastereomers **6a**–**c, 7a**–**d, 8a**–**e** and **9a**–**d**.

Entry	**6**/**7**/**8**/**9**	Yield [%]	Ratio^a^

1	**6a**, R = methyl	28	n.d
2	**6b**, R = (TMS)ethynyl	42	2:1
3	**6c**, R = phenylethynyl	42	1.6:1
4	**7a**, R = methyl	35	1.9:1
5	**7b**, R = (TMS)ethynyl	30	1.1:1
6	**7c**, R = phenylethynyl	30	1.4:1
7	**7d**, R = vinyl	69	2.3:1
8	**8a**, R = methyl, R^1^ = H	38	2.4:1
9	**8b**, R = (TMS)ethynyl, R^1^ = H	80	3.2:1^b^
10	**8c**, R = phenylethynyl, R^1^ = H	71	1.1:1
11	**8d**, R = vinyl, R^1^ = H	37	2.6:1
12	**8e**, R = vinyl, R^1^ = TCBoc	17	5.7:1
13	**9a**, R = methyl	40	2.4:1
14	**9b**, R = (TMS)ethynyl	77	–
15	**9c**, R = phenylethynyl	44	1:1
16	**9d**, R = vinyl	61	2.6:1

^a^Determined by ^1^H NMR; ^b^pure epimers were isolated by HPLC; n.d. = not determined.

**Scheme 4:**
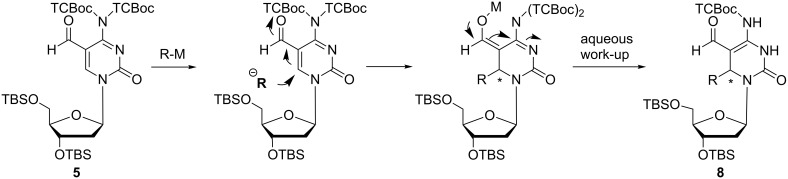
Proposed mechanism for the formation of 3,6-dihydrodeoxycytidine derivatives **8a**–**d** (M = Li, Mg).

Surprisingly, the reaction of derivative **5** bearing a *N*-(TCBoc)_2_ group with organometallic compounds (methylmagnesium bromide, THF, 0 °C → room temperature, lithium (trimethylsilyl)acetylide, THF, −50 °C, lithium phenylacetylide, THF, −78 °C → −50 °C, vinylmagnesium bromide, THF, 0 °C) afforded 3,6-dihydrodeoxycytidine derivatives **8a**–**e** as mixtures of diastereomers. In case of **8b** the diastereomers were separated by HPLC. Products arising from addition of the organometallic reagents to the aldehyde group (1,2-addition) were not observed. The formation of compounds **8a**–**d** can be explained assuming a Michael-type reaction of aldehyde **5** with organometallic reagents, subsequent isomerisation of the double bond followed by removal of one TCBoc group during the reaction and work-up as shown in Scheme 4.

Finally, cleavage of the TCBoc group was achieved by the action of the 10% Cd–Pb couple [32] on compounds **8a**–**d** in THF and 1.0 M aq NH_4_OAc to provide derivatives **9a**–**d** (Scheme 3).

## Conclusion

In summary, the reaction of 5fC derivatives **1**, **4**, and **5** with organometallic reagents (RMgBr, RLi) was investigated and enabled the synthesis of novel derivatives of 5-hydroxymethylcytosine and 5-formylcytosine: whereas aldehydes **1** and **4** afforded cytosine derivatives **2a**–**e**, **6a**–**c** and **7a**–**d**, the reaction of derivative **5** yielded 3,6-dihydrodeoxycytidine derivatives **8a**–**d** which subsequently after removal of the TCBoc group afforded derivatives **9a**–**d**. These new nucleobase modified 2’-deoxycytidine analogues can be used in the synthesis [29,33–34] of modified DNA oligomers for further studies of the TET-mediated processes which are of great importance in the emerging field of epigenetics. In addition they could find application as novel antivirals and/or as antimetabolites [35–36]. The majority of the obtained compounds contain functionalized side chains thus allowing further manipulations.

## Supporting Information

File 1 Experimental details and analytical data of all synthesized compounds.
