# Supplementary material for: Synthesis of novel derivatives of 5-hydroxymethylcytosine and 5-formylcytosine as tools for epigenetics
Source: Beilstein J Org Chem. 2014 Jan 3;10:7–11. doi: 10.3762/bjoc.10.2 (PMC3896258; doi:10.3762/bjoc.10.2)
Supplement: File 1 — Experimental details and analytical data of all synthesized compounds. [file Beilstein_J_Org_Chem-10-07-s001.pdf]

Supporting Information

for

Synthesis of novel derivatives of 5-hydroxymethylcytosine and 5-formylcytosine as tools for epigenetics

Anna Chentsova, Era Kapourani and Athanassios Giannis*

Address: Institut für Organische Chemie, Fakultät für Chemie und Mineralogie, Universität Leipzig, Johannisallee 29, 04103 Leipzig, Germany

Email: Athanassios Giannis - giannis@uni-leipzig.de

* Corresponding author

Experimental details and analytical data of all synthesized compounds

Table of Contents

General experimental methods: S3
3’,5’-Di(*tert*-butyldimethylsilyl)-5-(1-hydroxyethyl)-2’-deoxycytidine (**2a**) S4
5-(1-Hydroxyethyl)-2’-deoxycytidine (**3a**)........ S5
3’,5’-Di(*tert*-butyldimethylsilyl)-5-(1-hydroxy-3-(trimethylsilyl)prop-2-ynyl)-2’-deoxycytidine (**2b**) and 3’,5’-(*tert*-butyldimethylsilyl)-5-(1-hydroxyprop-2-ynyl)-2’-deoxycytidine (**2e**) S5
5-(1-Hydroxyprop-2-ynyl)-2’-deoxycytidine (**3b**) S7
3’,5’-Di(*tert*-butyldimethylsilyl)-5-(1-hydroxy-3-phenylprop-2-ynyl)-2’-deoxycytidine (**2c**) S8
5-(1-Hydroxy-3-phenylprop-2-ynyl)-2’-deoxycytidine (**3c**) S9
3’,5’-Di(*tert*-butyldimethylsilyl)-5-(1-hydroxyprop-2-enyl)-2’-deoxycytidine (**2d**) S9
5-(1-Hydroxyprop-2-enyl)-2’-deoxycytidine (**3d**)........ S10

3’,5’-Di(*tert*-butyldimethylsilyl)-N^4-(2,2,2-trichloro-*tert*-butyloxycarbonyl)-5-formyl-2’-deoxycytidine (**4**) S11
3’,5’-Di(*tert*-butyldimethylsilyl)-N^4, N^4-bis(2,2,2-trichloro-*tert*-butyloxycarbonyl)-5-formyl-2’-deoxycytidine (**5**) S12
3',5'-Di(*tert*-butyldimethylsilyl)-N^4-(2,2,2-trichloro-*tert*-butyloxycarbonyl)-5-(1-hydroxyethyl)-2’-deoxycytidine (**6a**) and 6-((2*R*,4*S*,5*R*)-4-(*tert*-butyldimethylsilyloxy)-5-((*tert*-butyldimethylsilyloxy)methyl)tetrahydrofuran-2-yl)-4-methyl-1*H*-pyrimido[4,5-*d*][1,3]oxazine-2,7(4*H*,6*H*)-dione (**7a**) S13
3',5'-Di(*tert*-butyldimethylsilyl)-N^4-(2,2,2-trichloro-*tert*-butyloxycarbonyl)-5-(1-hydroxy-3-(trimethylsilyl)prop-2-ynyl)-2’-deoxycytidine (**6b**) and 6-((2*R*,4*S*,5*R*)-4-(*tert*-butyldimethylsilyloxy)-5-((*tert*-butyldimethylsilyloxy)methyl)tetrahydrofuran-2-yl)-4-((trimethylsilyl)ethynyl)-1*H*-pyrimido[4,5-*d*][1,3]oxazine-2,7(4*H*,6*H*)-dione (**7b**) S14
3',5'-Di(*tert*-butyldimethylsilyl)-N^4-(2,2,2-trichloro-*tert*-butyloxycarbonyl)-5-(1-hydroxy-3-phenylprop-2-ynyl)-2’-deoxycytidine (**6c**) and 6-((2*R*,4*S*,5*R*)-4-(*tert*-butyldimethylsilyloxy)-5-((*tert*-butyldimethylsilyloxy)methyl)tetrahydrofuran-2-yl)-4-(phenylethynyl)-1*H*-pyrimido[4,5-*d*][1,3]oxazine-2,7(4*H*,6*H*)-dione (**7c**) S16
6-((2*R*,4*S*,5*R*)-4-(*tert*-Butyldimethylsilyloxy)-5-((*tert*-butyldimethylsilyloxy)methyl)tetrahydrofuran-2-yl)-4-vinyl-1*H*-pyrimido[4,5-*d*][1,3]oxazine-2,7(4*H*,6*H*)-dione (**7d**) S17
3’,5’-Di(*tert*-butyldimethylsilyl)-N^4-(2,2,2-trichloro-*tert*-butyloxycarbonyl)-3,6-dihydro-5-formyl-6-methyl-2’-deoxycytidine (**8a**) S18
3’,5’-Di(*tert*-butyldimethylsilyl)-N^4-(2,2,2-trichloro-*tert*-butyloxycarbonyl)-3,6-dihydro-5-formyl-6-(trimethylsilylethynyl)-2’-deoxycytidine (**8b**) S19
3’,5’-Di(*tert*-butyldimethylsilyl)-N^4-(2,2,2-trichloro-tert-butyloxycarbonyl)-3,6-dihydro-5-formyl-6-(2-phenylethynyl)-2’-deoxycytidine (**8c**) S21
3’,5’-Di(*tert*-butyldimethylsilyl)-N^4-(2,2,2-trichloro-*tert*-butyloxycarbonyl)-3,6-dihydro-5-formyl-6-vinyl-2’-deoxycytidine (**8d**) and 3’,5’-(*tert*-butyldimethylsilyl)-N^4,N^4-bis(2,2,2-trichloro-*tert*-butyloxycarbonyl)-3,6-dihydro-5-formyl-6-vinyl-2’-deoxycytidine (**8e**) S22
3’,5’-Di(*tert*-butyldimethylsilyl)-3,6-dihydro-5-formyl-6-methyl-2’-deoxycytidine (**9a**) S23
3',5'-Di(*tert*-butyldimethylsilyl)-3,6-dihydro-5-formyl-6-(trimethylsilylethynyl)-2’-deoxycytidine (**9b**) S24
3',5'-Di(*tert*-butyldimethylsilyl)-3,6-dihydro-5-formyl-6-(2-phenylethynyl)-2’-deoxycytidine (**9c**) S25
3',5'-Di(*tert*-butyldimethylsilyl)-3,6-dihydro-5-formyl-6-vinyl-2’-deoxycytidine **9d** S26
NMR Spectra: S27

General experimental methods:

All reactions were run under an atmosphere of argon unless otherwise indicated. Room temperature refers to 22 °C, ambient pressure to 1013 hPa. Reagents and anhydrous solvents were transferred via oven-dried syringe or cannula. Flasks were flame dried under vacuum and cooled under a constant stream of argon. Tetrahydrofuran (THF) was distilled under argon from potassium, dichloromethane from SICAPENT (phosphorus pentoxide on solid support with indicator). Pyridine was purchased from Acros (anhydrous over molecular sieves). All other chemicals were purchased from ABCR, Acros, Aldrich, Alfa Aesar, TCI Europe and VWR at the highest commercially available purity and used as such. Reactions were monitored by thin layer chromatography using Merck silica gel 60 F_{254} TLC aluminium sheets and visualized under a UV lamp and/or with anisaldehyde staining solution. Chromatographic purification was performed as flash chromatography on Acros silica gel 35–70, 60 Å, using a forced flow of eluent (method of Still) or MACHEREY-NAGEL silica gel 60 M C18-Reversed phase. Concentration under reduced pressure was performed by rotary evaporation at 40 °C at the appropriate pressure. NMR spectra were recorded on a Varian Mercury plus 300 (operating at 300 MHz for ^{1}H and 75 MHz for ^{13}C acquisitions), a Varian Mercury plus 400 (operating at 400 MHz for ^{1}H, 100 MHz for ^{13}C). Chemical shifts δ are reported in ppm with the solvent resonance as internal standard (chloroform-d_1: 7.26 (^{1}H NMR), 77.16 (^{13}C NMR); methanol-d_4: 3.31 (^{1}H NMR), 49.00 (^{13}C NMR); dimethyl sulfoxide-d_6: 2.50 (^{1}H NMR), 39.52 (^{13}C NMR). Coupling constants *J* are given in Hertz (Hz). Multiplicities are classified by the following abbreviations: s = singlet, d = doublet, t = triplet and combinations thereof, or m = multiplet or bs = broad signal. High resolution mass spectra were obtained on a Bruker Daltonics ESI-FT-ICR-MS APEX II [7 T]. IR spectra were obtained on an ATI/MATTSON Genesis FT-IR as thin film or KBr disk. Absorbance frequencies are reported in reciprocal centimeters (cm^{-1}). Melting points were measured with a Büchi "Melting Point B-540” and are uncorrected. Optical rotation data was obtained with a Schmidt+Haensch Polartronic MHZ-8 at the sodium-D line (589 nm) using a 50 mm path-length cell in the solvent and concentration indicated.

In ^{13}C NMR reports of diastereomeric mixtures one value is given in the case of overlapped signals, otherwise the corresponding signal is given in parentheses.

In ^{1}H NMR reports of diastereomeric mixtures protons marked with asterisk indicate the signals of corresponding minor diastereomer. The ratio of the diastereomers was determined by ^{1}H NMR.

3',5'-Di(*tert*-butyldimethylsilyl)-5-(1-hydroxyethyl)-2'-deoxycytidine (2a)

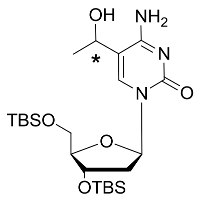

2a

To an ice-cooled solution of aldehyde **1** (100 mg, 207 µmol, 1.0 equiv) in THF (2.00 mL) methylmagnesium bromide (3.0 M in Et_2O; 138 µL, 413 µmol, 2.0 equiv) was added dropwise and the solution stirred at 0 °C. After one hour the reaction mixture was allowed to warm to room temperature and stirred at this temperature for four hours. Subsequently additional amount of methylmagnesium bromide (3.0 M in Et_2O; 69.0 µL, 207 µmol, 1.0 equiv) was added and the reaction mixture was stirred overnight at room temperature. Additional portion of methylmagnesium bromide (3.0 M in Et_2O; 69.0 µL, 207 µmol, 1.0 equiv) was then added and the reaction mixture was stirred for another three hours at room temperature before it was quenched with saturated aqueous NH_4Cl (5 mL) and diluted with DCM (10 mL). The phases were separated and the aqueous layer was extracted with DCM (2 × 10 mL). The combined organic layers were washed with brine (10 mL), dried over $MgSO_4$ and the solvents were removed under reduced pressure. The crude product was purified by column chromatography (DCM/MeOH, 60:1 → 15:1 *v/v*) to yield a mixture of diastereomeric alcohols **2a** with a ratio of 1.1:1 (99.0 mg, 198 µmol, 96%) as a colourless oil.

R_f: 0.37 (DCM/MeOH = 15:1 *v/v*).

IR: (KBr) ν_{max} = 2858, 2954, 2857, 1656, 1435, 1095, 838, 780, 554 cm^{-1}.

^{1}H-NMR: (400 MHz, CD_3OD) δ (ppm) 7.76 (s, 1H, **H-6**), 7.73 (s, 1H, **H-6***), 6.30-6.26 (m, 2H, **H-1'**, **H-1'***), 4.74-4.44 (m, 2H, C**H**-CH_3, C**H***-CH_3), 4.47-4.42 (m, 2H, **H-3'**, **H-3'***), 3.96-3.95 (m, 2H, **H-4'**, **H-4'***), 3.82-3.81 (m, 4H, **H-5'**, **H-5'***), 2.36-2.30 (m, 2H, **H-2'**, **H-2'***), 2.11-2.03 (m, 2H, **H-2'**, **H-2'***), 1.43-1.42 (m, 6H, CH-C**H$_3$**, CH-C**H**$_3$*), 0.93-0.91 (m, 18H, Si-C(C**H$_3$**)$_3$, Si-C(C**H$_3$**)$_3$*), 0.91 (s, 18H, Si-C(C**H$_3$**)$_3$, Si-C(C**H$_3$**)$_3$*), 0.12 (s, 12H, Si-(C**H$_3$**)$_2$, Si-(C**H$_3$**)$_2$*), 0.11 (s, 12H, Si-(C**H$_3$**)$_2$, Si-(C**H$_3$**)$_2$*).

^{13}C-NMR: (100 MHz, CD_3OD) δ (ppm) 165.0 (**C-4**), 157.9 (**C-2**), 138.3 (**C-6**), 89.5 (89.4) (**C-4'**), 87.4 (87.3) (**C-1'**), 73.9 (73.8) (**C-3'**), 65.7 (**C**H-CH_3), 64.3 (**C-5'**), 42.3 (42.1) (**C-2'**), 26.5 (Si-C(**C**H$_3$)$_3$), 26.2 (Si-C(**C**H$_3$)$_3$), 22.9 (22.8) (CH-**C**H$_3$), 19.2 (Si-**C**(CH$_3$)$_3$), 18.8 (Si-**C**(CH$_3$)$_3$), -4.6 (Si-**C**H$_3$), -4.7 (Si-**C**H$_3$), -5.2 (Si-**C**H$_3$). **C-5** is not observed.

HR-MS: (ESI positive, MeOH), $[M+H]^+$ calcd for $C_{23}H_{46}N_3O_5Si_2$: 500.29705, found: 500.29727, $[2M+H]^+$ calcd for $C_{46}H_{91}N_6O_{10}Si_4$: 999.58683, found: 999.58716.

5-(1-Hydroxyethyl)-2'-deoxycytidine (3a)

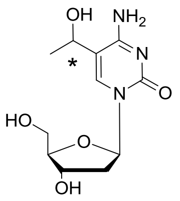

3a

In a polypropylene tube the mixture of epimeric alcohols **2a** (98.0 mg, 196 µmol, 1.0 equiv) was dissolved in DCM (10.0 mL) and cooled to 0 °C. Subsequently HF·Et_3N (64.0 µL, 392 µmol, 2.0 equiv) was added in one portion. The solution was allowed to warm to room temperature and stirred overnight. After addition of TMSOMe (0.700 mL, 5.08 mmol, 26 equiv) the reaction mixture was stirred for another 30 min. The suspension was centrifuged for 10 min at 5000 rpm, the supernatant was collected and the residue was resuspended in DCM and centrifuged for 10 min at 5000 rpm. The procedure was repeated one more time and the crude product was purified by reversed phase column chromatography (H_2O/MeCN 4:1 *v/v*) to yield a mixture of diastereomeric alcohols **3a** (40.0 mg, 147 µmol, 75%) as a colourless oil. A ratio could not be calculated.

IR: (CCl_4) ν_{max} = 3441, 2924, 2360, 1736, 1652, 1383, 1247, 1026, 795, 602, 456 cm^{-1}.

^{1}H-NMR: (300 MHz, CD_3OD) δ (ppm) 8.06 -8.05 (m, 2H, **H-6, H-6***), 6.31-6.26 (m, 2H, **H-1'**, **H-1'***), 4.75-4.68 (m, 2H, C**H**-CH_3, C**H**-CH_3*), 4.42-4.37 (m, 2H, **H-3'**, **H-3'***), 3.98-3.94 (m, 2H, **H-4'**, **H-4'***), 3.87-3.81 (m, 2H, **H-5'**, **H-5'***), 3.78-3.73 (m, 2H, **H-5'**, **H-5'***), 2.42-2.34 (m, 2H, **H-2'**, **H-2'***), 2.21-2.12 (m, 2H, **H-2'**, **H-2'***), 1.48-1.46 (m, 6H, CH-C**H_3**, CH-C**H_3***).

^{13}C-NMR: (100 MHz, CD_3OD) δ (ppm) 166.1 (**C-4**), 157.9 (**C-2**), 139.3 (**C-6**), 111.8 (111.7) (**C-5**), 88.90 (88.88) (**C-4'**), 87.7 (87.6) (**C-1'**), 71.92 (71.90) (**C-3'**), 65.8 (65.7) (**C**H-CH_3), 62.6 (**C-5'**), 42.21 (42.18) (**C-2'**), 22.42 (22.40) (CH-**C**H_3).

HR-MS: (ESI positive, MeOH), $[M+Na]^+$ calcd for $C_{11}H_{17}N_3O_5Na$: 294.10604, found: 294.10617, $[2M+Na]^+$ calcd for $C_{22}H_{34}N_6O_{10}Na$: 565.22286, found: 565.22330.

3',5'-Di(*tert*-butyldimethylsilyl)-5-(1-hydroxy-3-(trimethylsilyl)prop-2-ynyl)-2'-deoxycytidine (2b) and 3',5'-di(*tert*-butyldimethylsilyl)-5-(1-hydroxyprop-2-ynyl)-2'-deoxycytidine (2e)

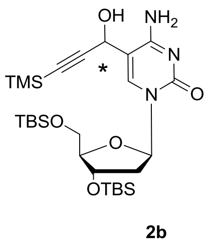

2b

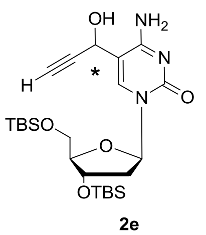

2e

To a stirred solution of trimethylsilylacetylene (671 µL, 4.71 mmol, 4.0 equiv) in THF (9.00 mL) *n*-BuLi (2.5 M in *n*-hexane; 1.89 mL, 4.71 mmol, 4.0 equiv) was added dropwise at −40 °C. The reaction mixture was warmed to −20 °C. After stirring at this temperature for one hour reaction mixture was cooled down to −40 °C and a solution of aldehyde **1** (570 mg, 1.18 mmol, 1.0 equiv) in THF (11.0 mL) was added dropwise. The reaction was stirred at −40 °C for three hours, thereafter at −20 °C for 30 min. The reaction was quenched with saturated aqueous NH_4Cl (20 mL), diluted with Et_2O (30 mL) and the aqueous layer extracted with Et_2O (3 × 10 mL). The combined organic phases were dried over $MgSO_4$ and the solvents were removed under reduced pressure. Purification by silica gel chromatography (DCM/MeOH, 60:1 → 15:1 *v/v*) yielded mixture of diastereomeric alcohols **2b** with a ratio of 1.9:1 (296 mg, 509 µmol, 43 %) as yellow solid and a mixture of diastereomeric alcohols **2e** with a ratio of 1.2:1 (154 mg, 302 µmol, 26%) as a slight yellow oil.

2b:

R_f: 0.21 (DCM/MeOH = 30:1 *v/v*).

IR: (KBr) ν_{max} = 3427, 2955, 2858, 1658, 1604, 1385, 1253, 1094, 840, 779, 555 cm^{-1}.

^{1}H-NMR: (300 MHz, CD_3OD) δ (ppm) 7.96 (s, 1H, **H-6**), 7.95 (s, 1H, **H-6***), 6.24-6.17 (m, 2H, **H-1'**, **H-1'***), 5.30 (s, 2H, C**H**-OH, C**H***-OH), 4.45-4.41 (m, 2H, **H-3'**, **H-3'***), 4.03-3.98 (m, 2H, **H-4'**, **H-4'***), 3.83-3.81 (m, 4H, **H-5'**, **H-5'***), 2.48-2.40 (m, 2H, **H-2'**, **H-2'***), 2.14-2.01 (m, 2H, **H-2'**, **H-2'***), 0.92-0.91 (m, 36H, 2xSi-C(C**H$_3$**)$_3$, 2xSi-C(C**H$_3$**)$_3$*), 0.19-0.18 (m, 18H, Si-(C**H$_3$**)$_3$, Si-(C**H$_3$**)$_3$*), 0.12-0.11 (m, 24H, 2xSi-(C**H$_3$**)$_2$, 2xSi-(C**H$_3$**)$_2$*).

^{13}C-NMR: (100 MHz, $CDCl_3$) δ (ppm) 162.8 (162.6) (**C-4**), 154.9 (**C-2**), 140.2 (139.9) (**C-6**), 106.6 (**C-5**), 102.3 (102.1) (**C**≡C-TMS), 92.8 (92.5) (C≡**C**-TMS), 88.7 (88.5) (**C-4'**), 87.3 (87.1) (**C-1'**), 72.8 (72.5) (**C-3'**), 63.5 (63.4) (**C-5'**), 60.3 (60.2) (**C**H-OH), 42.1 (41.8) (**C-2'**), 26.19 (26.16) (Si-C(**C**H$_3$)$_3$), 26.0 (Si-C(**C**H$_3$)$_3$), 18.59 (18.57) (Si-**C**(CH$_3$)$_3$), 18.2 (Si-**C**(CH$_3$)$_3$), 0.05 (Si-(**C**H$_3$)$_3$), -4.31 (-4.34) (Si-**C**H$_3$), -4.63 (-4.65) (Si-**C**H$_3$), -5.06 (Si-**C**H$_3$), -5.11 (Si-**C**H$_3$).

HR-MS: (ESI positive, EtOAc), $[M+Na]^+$ calcd for $C_{27}H_{51}N_3O_5Si_3Na$: 604.30287, found: 604.30327, $[2M+H]^+$ calcd for $C_{54}H_{103}N_6O_{10}Si_6$: 1163.63458, found: 1163.63592, $[2M+Na]^+$ calcd for $C_{54}H_{102}N_6O_{10}Si_6Na$: 1185.61652, found: 1185.61555.

2e:

R_f: 0.15 (DCM/MeOH = 30:1 *v/v*).

IR: (CCl_4) ν_{max} = 3444, 2929, 1660, 1361, 1256, 1093, 837, 780 cm^{-1}.

^{1}H-NMR: (300 MHz, CD_3OD) δ (ppm) 8.01 (s, 1H, **H-6***), 7.99 (s, 1H, **H-6**), 6.26-6.20 (m, 2H, **H-1'**, **H-1'***), 5.34-5.32 (m, 2H, C**H**-OH, C**H***-OH), 4.47-4.44 (m, 2H, **H-3'**, **H-3'***), 4.01-3.98 (m, 2H, **H-4'**, **H-4'***), 3.82-3.79 (m, 4H, **H-5'**, **H-5'***), 3.15-3.13 (m, 2H, C≡C-**H,** C≡C-**H***), 2.47-2.38 (m, 2H, **H-2'**, **H-2'***), 2.13-2.03 (m, 2H, **H-2'**, **H-2'***), 0.91-0.90 (m, 36H, 2xSi-C(C**H$_3$**)$_3$, 2xSi-C(C**H$_3$**)$_3$*), 0.11 (s, 24H, 2xSi-(C**H$_3$**)$_2$, 2xSi-(C**H$_3$**)$_2$*).

^{13}C-NMR: (100 MHz, CD_3OD) δ (ppm) 164.5 (**C-4**), 157.7 (**C-2**), 140.3 (**C-6**), 108.4 (**C-5**), 89.8 (89.7) (**C-4'**), 88.1 (88.0) (**C-1'**), 82.4 (82.3) (**C**≡C-H), 76.72 (76.69) (C≡**C**-H), 74.2

(74.1) (**C-3'**), 64.38 (64.35) (**C-5'**), 59.81 (59.77) (**C**H-OH), 42.4 (42.3) (**C-2'**), 26.43 (26.41) (Si-C(**C**H_3)$_3$), 26.21 (26.18) (Si-C(**C**H_3)$_3$), 19.2 (19.1) (Si-**C**(CH_3)$_3$), 18.8 (Si-**C**(CH_3)$_3$), -4.56 (-4.55) (Si-**C**H_3), -4.7 (Si-**C**H_3), -5.2 (Si-**C**H_3), -5.30 (-5.32) (Si-**C**H_3).

HR-MS: (ESI positive, MeOH), $[M+H]^+$ calcd for $C_{24}H_{44}N_3O_5Si_2$: 510.2814, found: 510.28181, $[2M+H]^+$ calcd for $C_{48}H_{87}N_6O_{10}Si_4$: 1019.55553, found: 1019.55476.

5-(1-Hydroxyprop-2-ynyl)-2'-deoxycytidine (3b)

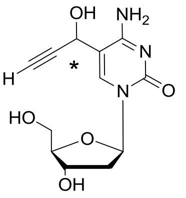

3b

To a solution of epimeric alcohols **2b** (68.6 mg, 118 µmol, 1.0 equiv) in EtOAc (2.80 mL) in a polypropylene tube pyridine (44.0 µL, 542 µmol, 4.6 equiv) and HF·pyridine (70% HF, 13.5 µL, 514 µmol, 4.4 equiv) were added subsequently. After stirring for six hours another portion of pyridine (44.0 µL, 542 µmol, 4.6 equiv) and HF·pyridine (70% HF, 13.5 µL, 514 µmol, 4.4 equiv) was added to the reaction mixture. After stirring for 23 hours an additional portion of pyridine (44.0 µL, 542 µmol, 4.6 equiv) and HF·pyridine (70% HF, 13.5 µL, 514 µmol, 4.4 equiv) was added and the stirring was continuing overnight. After addition of TMSOMe (0.50 mL, 3.63 mmol, 31 equiv) and stirring for one hour, a suspension was formed and centrifuged for 10 min at 3000 rpm. The supernatant was collected, the residue resuspended in EtOAc and centrifuged for 10 min at 3000 rpm. The crude product was purified by reversed phase column chromatography (H_2O/MeCN 4:1 *v/v*) to yield a mixture of diastereomeric alcohols **3b** (20.0 mg, 71.1 µmol, 60%) as a white solid. A ratio could not be calculated.

IR: (CCl_4) ν_{max} = 3444, 2921, 2239, 1733, 1625, 1384, 711, 602, 454 cm^{-1}.

^{1}H NMR (400 MHz, CD_3OD) δ (ppm) 8.22-8.20 (m, 2H, **H-6**, **H-6***), 6.27-6.24 (m, 2H, **H-1'**, **H-1'***), 5.31-5.30 (m, 2H, C**H**-OH, C**H***-OH), 4.37-4.33 (m, 2H, **H-3'**, **H-3'***), 3.96-3.94 (m, 2H, **H-4'**, **H-4'***), 3.81-3.71 (m, 4H, **H-5'**, **H-5'***), 3.13-3.12 (m, 2H, C≡C-**H,** C≡C-**H***), 2.42-2.36 (m, 2H, **H-2'**, **H-2'***), 2.17-2.09 (m, 2H, **H-2'**, **H-2'***).

^{13}C-NMR: (75 MHz, CD_3OD) δ (ppm): 165.5 (**C-4**), 157.8 (**C-2**), 141.1 (**C-6**), 108.0 (**C-5**), 89.0 (**C-4'**), 87.82 (87.79) (**C-1'**), 82.4 (**C**≡C-H), 76.73 (76.66) (C≡**C**-H), 72.20 (72.17) (**C-3'**), 63.00 (62.95) (**C-5'**), 60.0 (**C**H-OH), 42.1 (**C-2'**).

HR-MS: (ESI positive, MeOH), $[M+H]^+$ calcd for $C_{12}H_{16}N_3O_5$: 282.10845, found: 282.10885, $[M+Na]^+$ calcd for $C_{12}H_{15}N_3O_5Na$: 304.09039, found: 304.09060, $[2M+H]^+$ calcd for $C_{24}H_{31}N_6O_{10}$: 563.20962, found: 563.20940.

3',5'-Di(*tert*-butyldimethylsilyl)-5-(1-hydroxy-3-phenylprop-2-ynyl)-2'-deoxycytidine (2c)

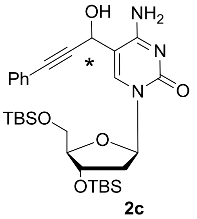

2c

To a stirred solution of phenylacetylene (91.0 µL, 827 µmol, 4.0 equiv) in THF (1.50 mL) at −78 °C *n*-BuLi (2.5 M in *n*-hexane; 331 µL, 827 µmol, 4.0 equiv) was added dropwise. After stirring the reaction mixture at this temperature for 90 min a solution of aldehyde **1** (100 mg, 207 µmol, 1.0 equiv) in THF (2.00 mL) was added dropwise. The reaction was stirred at −78 °C for three hours thereafter at −50 °C till no starting material was detected by TLC. The reaction was quenched with saturated aqueous NH_4Cl (5 mL), diluted with DCM (10 mL). The aqueous layer was extracted with DCM (3 × 10 mL). The combined organic phases were washed with brine (10 mL) and dried over $MgSO_4$ and the solvents were removed under reduced pressure. Purification by silica gel chromatography (DCM/MeOH, 60:1 → 15:1 *v/v*) yielded a mixture of diastereomeric alcohols **2c** with a ratio of 1.2:1 (82.0 mg, 140 µmol, 68%) as a slight yellow oil.

R_f: 0.24 (DCM/MeOH = 30:1 *v/v*).

IR: (KBr) ν_{max} = 3420, 2929, 1655, 1470, 1384, 1095, 838, 780, 558 cm^{-1}.

^{1}H-NMR: (300 MHz, CD_3OD) δ (ppm) 8.08 (d, *J* = 0.8 Hz, 1H, **H-6***), 8.06 (d, *J* = 0.7 Hz, 1H, **H-6**), 7.49-7.45 (m, 4H, C**H** aromat., C**H*** aromat.), 7.40-7.36 (m, 6H, C**H** aromat., C**H*** aromat.), 6.29-6.23 (m, 2H, **H-1'**, **H-1'***), 5.58 (5.57) (m, 2H, C**H**-OH, C**H***-OH), 4.47-4.42 (m, 2H, **H-3'**, **H-3'***), 4.02-3.99 (m, 2H, **H-4'**, **H-4'***), 3.78-3.74 (m, 4H, **H-5'**, **H-5'***), 2.51-2.42 (m, 2H, **H-2'**, **H-2'***), 2.15-2.05 (m, 2H, **H-2'**, **H-2'***), 0.93 (s, 18H, Si-C(C**H3**)3, Si-C(C**H3**)3*), 0.88 (s, 9H, Si-C(C**H3**)3), 0.87 (s, 9H, Si-C(C**H3**)3*), 0.11 (m, 12H, Si-(C**H3**)2, Si-(C**H3**)2*), 0.04 (m, 6H), 0.03 (s, 3H), 0.02 (s, 3H).

^{13}C-NMR: (75 MHz, CD_3OD) δ (ppm) 164.9 (**C-4**), 158.1 (**C-2**), 140.2 (**C-6**), 132.7 (132.6) (**C** aromat.), 130.03 (130.00) (**C** aromat.), 129.62 (129.61) (**C** aromat.), 123.3 (**C** aromat.), 89.75 (89.72), 88.1 (88.0), 87.6 (87.5), 74.3 (74.2) (**C-3'**), 64.6 (**C-5'**), 60.6 (60.5) (**C**H-OH), 42.46 (42.40) (**C-2'**), 26.39 (26.36) (Si-C(**C**H3)3), 26.2 (Si-C(**C**H3)3), 19.11 (19.08) (Si-**C**(CH3)3), 18.8 (Si-**C**(CH3)3), -4.6 (Si-**C**H3), -4.7 (Si-**C**H3), -5.2 (-5.3) (Si-**C**H3), -5.3 (-5.4) (Si-**C**H3).

HR-MS: (ESI positive, MeOH), $[M+H]^+$ calcd for $C_{30}H_{48}N_3O_5Si_2$: 586.31270, found: 586.31259, $[2M+H]^+$ calcd for $C_{60}H_{95}N_6O_{10}Si_4$: 1171.61813, found: 1171.61902.

5-(1-Hydroxy-3-phenylprop-2-ynyl)-2'-deoxycytidine (3c)

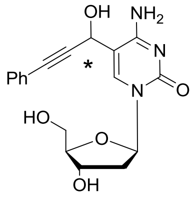

3c

In a polypropylene tube the mixture of epimeric alcohols **2c** (75.0 mg, 128 µmol, 1.0 equiv) was dissolved in EtOAc (3.70 mL), subsequently pyridine (52.0 µL, 640 µmol, 5.0 equiv) and HF·pyridine (70% HF, 48.0 µL, 1.92 mmol, 15 equiv) were added and the reaction mixture was stirred overnight at room temperature. After addition of TMSOMe (0.50 mL, 3.84 mmol, 30 equiv) and stirring for one hour the suspension was centrifuged for 10 min at 3000 rpm and the crude product was purified by reversed phase column chromatography (H_2O/MeCN 4:1 → 2:1 *v/v*) to yield a mixture of diastereomeric alcohols **3c** with a ratio of 1.1:1 (33.0 mg, 92.3 µmol, 72%) as a colourless oil.

IR: (CCl_4) ν_{max} = 3355, 2922, 1660, 1488, 1299, 1093, 1029, 758, 607, 454 cm^{-1}.

^{1}H-NMR: (300 MHz, CD_3OD) δ (ppm) 8.21 (s, 1H, **H-6'**), 8.19 (s, 1H, **H-6'***) 7.49-7.46 (m, 4H, C**H** aromat., C**H*** aromat.), 7.36-7.34 (m, 6H, C**H** aromat., C**H*** aromat.), 6.25 (m, 2H, **H-1'**, **H-1'***), 5.54 (s, 2H, C**H**-OH**,** C**H***-OH), 4.36-4.32 (m, 2H, **H-3'**, **H-3'***), 3.97-3.92 (m, 2H, **H-4'**, **H-4'***), 3.72-3.68 (m, 4H, **H-5'**, **H-5'***), 2.45-2.36 (m, 2H, **H-2'**, **H-2'***), 2.18-2.09 (m, 2H, **H-2'**, **H-2'***).

^{13}C-NMR: (100 MHz, CD_3OD) δ (ppm) 165.5 (**C-4**), 157.8 (**C-2**), 141.0 (140.9) (**C-6**), 132.7 (**C** aromat.), 129.9 (**C** aromat.), 129.6 (**C** aromat.), 123.5 (**C** aromat.), 108.2 (**C**-5), 89.03 (88.97) (**C**-4'), 87.9 (87.8), 87.70 (87.68), 87.5 (87.4), 72.3 (72.2) (**C**-3'), 63.04 (62.98), 60.8 (60.7), 42.08 (42.06) (**C**-2').

HR-MS: (ESI positive, MeOH), $[M+H]^+$ calcd for $C_{18}H_{20}N_3O_5$: 358.13975, found: 358.13957, $[M+Na]^+$ calcd for $C_{18}H_{19}N_3O_5Na$: 380.12169, found: 380.12140, $[2M+H]^+$ calcd for $C_{36}H_{39}N_6O_{10}$: 715.27222, found: 715.27221

3',5'-Di(*tert*-butyldimethylsilyl)-5-(1-hydroxyprop-2-enyl)-2'-deoxycytidine (2d)

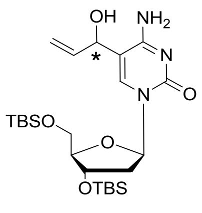

2d

To an ice-cooled solution of aldehyde **1** (50.0 mg, 103 µmol, 1.0 equiv) in THF (1.00 mL) vinylmagnesium bromide (1.0 M in THF; 155 µL, 155 µmol, 1.5 equiv) was added dropwise. After stirring for 20 min the reaction mixture was allowed to warm to room temperature and

was stirred for additional two hours. Subsequently an additional amount of vinylmagnesium bromide (1.0 M in THF; 258 µL, 258 µmol, 2.5 equiv) was added at 0 °C and the reaction mixture was stirred overnight at room temperature. The reaction was quenched with saturated aqueous NH_4Cl (5 mL) and diluted with Et_2O (10 mL). The phases were separated and the aqueous layer was extracted with Et_2O (2 × 5 mL). The combined organic layers were washed with brine (10 mL), dried over $MgSO_4$ and the solvents were removed under reduced pressure. The crude product was purified by column chromatography (DCM/MeOH, 60:1 → 15:1 *v/v*) to yield a mixture of diastereomeric alcohols **2d** with a ratio of 1.1:1 (41.0 mg, 0.19 mmol, 77%) as a slight yellow solid.

R_f: 0.36 (DCM/MeOH = 15:1 *v/v*).

IR: (KBr) ν_{max} = 3420, 1638, 1428, 1385, 1093, 838, 780, 550 cm^{-1}.

^{1}H-NMR: (300 MHz, CD_3OD) δ (ppm) 7.75 (s, 1H, **H-6***), 7.73 (s, 1H, **H-6**), 6.29-6.24 (m, 2H, **H-1'**, **H-1'***), 6.01-5.95 (m, 2H, C**H**=CH$_2$, C**H***=CH$_2$), 5.43-5.35 (m, 2H, CH=C**H$_2$**, CH=C**H$_2$***), 5.28-5.23 (m, 2H, CH=C**H$_2$**, CH=C**H$_2$***), 5.06-5.04 (m, 2H, C**H**-OH, C**H***-OH), 4.46-4.43 (m, 2H, **H-3'**, **H-3'***), 3.99-3.92 (m, 2H, **H-4'**, **H-4'***), 3.81-3.80 (m, 4H, **H-5'**, **H-5'***), 2.39-2.31 (m, 2H, **H-2'**, **H-2'***), 2.14-1.98 (m, 2H, **H-2'**, **H-2'***), 0.93 (s, 18H, Si-C(C**H$_3$**)$_3$, Si-C(C**H$_3$**)$_3$*), 0.91 (s, 18H, Si-C(C**H$_3$**)$_3$, Si-C(C**H$_3$**)$_3$*), 0.12 (s, 12H, Si-(C**H$_3$**)$_2$ Si-(C**H$_3$**)$_2$*), 0.11 (s, 12H, Si-(C**H$_3$**)$_2$, Si-(C**H$_3$**)$_2$*).

^{13}C-NMR: (75 MHz, CD_3OD) δ (ppm) 165.1 (**C-4**), 157.8 (**C-2**), 139.7 (**C-6**), 139.04 (138.97) (**C**H=CH$_2$), 116.7 (116.6) (CH=**C**H$_2$), 89.52 (89.47) (**C-4'**), 87.5 (**C-1'**), 73.96 (73.94), 70.7 (70.5), 64.4 (**C-5'**), 42.34 (42.26) (**C-2'**), 26.5 (Si-C(**C**H$_3$)$_3$), 26.4 (Si-C(**C**H$_3$)$_3$), 19.2 (Si-**C**(CH$_3$)$_3$), 18.8 (Si-**C**(CH$_3$)$_3$), -4.5 (Si-**C**H$_3$), -4.7 (Si-**C**H$_3$), -5.18 (Si-**C**H$_3$), -5.23 (Si-**C**H$_3$). **C-5** is not observed.

HR-MS: (ESI positive, $CHCl_3$/MeOH), $[M+H]^+$ calcd for $C_{24}H_{46}N_3O_5Si_2$: 512.29705, found: 512.29713.

5-(1-Hydroxyprop-2-enyl)-2'-deoxycytidine (3d)

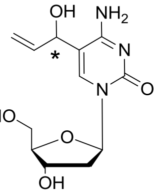

3d

In a polypropylene tube mixture of epimeric alcohols **2d** (134 mg, 262 µmol, 1.0 equiv) was dissolved in EtOAc (6.00 mL). Subsequently pyridine (97.0 µL, 1.20 mmol, 4.6 equiv) and HF·pyridine (70% HF, 30.0 µL, 1.15 mmol, 4.4 equiv) were added and the solution was stirred for six hours. An additional portion of pyridine (97.0 µL, 1.20 mmol, 4.6 equiv) and HF·pyridine (70% HF, 30.0 µL, 1.15 mmol, 4.4 equiv) was added to the reaction mixture which was stirred for 46 hours. After the addition of TMSOMe (1.00 mL, 7.24 mmol, 28 equiv) a suspension was formed, which was stirred for two hours and centrifuged for 10 min at 3000 rpm. The supernatant was then collected and the residue resuspended in EtOAc and centrifuged for 10 min at 3000 rpm. The procedure was repeated one more time. The

reaction yielded a mixture of diastereomeric alcohols **3d** (54.0 mg, 191 µmol, 73%) as a slight yellow oil. A ratio could not be calculated.

IR: (KBr) ν_{max} = 3419, 1656, 1603, 1516, 1480, 1300, 1257, 1094, 1054, 931, 789, 765, 545 cm^{-1}.

^{1}H-NMR: (400 MHz, CD_3OD) δ (ppm) 8.02-8.01 (m, 2H, **H-6**, **H-6***), 6.26-6.23 (m, 2H, **H-1'**, **H-1'***), 6.12-5.93 (m, 2H, C**H**=CH$_2$, C**H**=CH$_2$*), 5.42-5.36 (m, 2H, CH=C**H$_2$**, CH=C**H$_2$***), 5.27-5.24 (m, 2H, CH=C**H$_2$**, CH=C**H$_2$***), 5.04-5.02 (m, 2H, C**H**-OH, C**H***-OH), 4.38-4.34 (m, 2H, **H-3'**, **H-3'***), 3.95-3.92 (m, 2H, **H-4'**, **H-4'***), 3.83-3.66 (m, 4H, **H-5'**, **H-5'***), 2.39-2.33 (m, 2H, **H-2'**, **H-2'***), 2.16-2.10 (m, 2H, **H-2'**, **H-2'***).

^{13}C-NMR: (75 MHz, CD_3OD) δ (ppm) 165.7 (**C-4**), 157.7 (**C-2**), 140.6 (**C-6**), 139.1 (139.0) (**C**H=CH$_2$), 116.51 (116.45) (CH=**C**H$_2$), 109.5 (**C-5**), 88.9 (**C-4'**), 87.6 (**C-1'**), 71.87 (71.85) (**C-3'**), 70.8 (70.70) (**C**H-OH), 62.6 (**C-5'**), 42.13 (42.09) (**C-2'**).

HR-MS: (ESI positive, MeOH), $[M+H]^+$ calcd for $C_{12}H_{18}N_3O_5$: 284.12410, found: 284.12412, $[M+Na]^+$ calcd for $C_{12}H_{17}N_3O_5Na$: 306.10604, found: 306.10606.

3',5'-Di(*tert*-butyldimethylsilyl)-*N*^{4}-(2,2,2-trichloro-*tert*-butyloxycarbonyl)-5-formyl-2'-deoxycytidine (4)

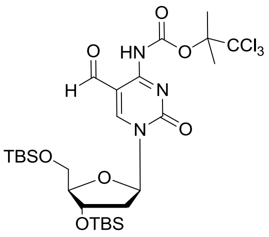

4

To a solution of aldehyde **1** (0.500 g, 1.03 mmol, 1.0 equiv) in DCM (4.00 mL) pyridine (100 µL, 1.24 mmol, 1.2 equiv) was added. The reaction mixture was cooled to 0 °C whereby the solution of TCBocCl (0.300 g, 1.25 mmol, 1.2 equiv) in DCM (2.00 mL) was added dropwise. The resulting clear solution was warmed to room temperature and stirred overnight. Subsequently it was poured into ice. After the phases were separated and the aqueous layer was extracted with DCM (3 × 5 mL), the combined organic phases were washed several times with saturated aqueous $CuSO_4$ solution and dried over $MgSO_4$. The solvents were removed under reduced pressure. The crude product was purified by column chromatography (Hex/EtOAc, 6:1 → 1:1 *v/v*) to yield **4** (395 mg, 5.75 µmol, 56%) as a yellow solid.

R$_f$: 0.48 (Hex/EtOAc = 2:1 *v/v*).

IR: (CCl_4) ν_{max} = 2954, 2857, 1778, 1663, 1564, 1489, 1386, 1254, 1142, 1029, 837, 782 cm^{-1}.

^{1}H-NMR: (400 MHz, $CDCl_3$) δ (ppm) 10.63 (s, 1H, N**H**), 9.46 (s, 1H, C**H**O), 8.71 (s, 1H, **H-6**), 6.19 (dd, *J* = 6.0, 6.0 Hz, 1H, **H-1'**), 4.35-4.32 (m, 1H, **H-3'**), 4.10-4.07 (m, 1H, **H-4'**), 3.98 (dd, *J* = 11.7, 2.5 Hz, 1H, **H-5'**), 3.78 (dd, *J* = 11.7, 2.5 Hz, 1H**, H-5'**), 2.71-2.65 (m, 1H,

H-2'), 2.13-2.06 (m, 1H, **H-2'**), 2.00-1.99 (m, 6H, C(C**H**$_3$)$_2$), 0.89 (s, 18H, 2xSi-C(C**H$_3$**)$_3$), 0.10 (s, 3H, Si-C**H$_3$**), 0.07 (s, 6H, 2xSi-C**H$_3$**), 0.06 (s, 3H, Si-C**H$_3$**).

^{13}C-NMR: (75 MHz, CDCl$_3$) δ (ppm) 187.3 (**C**HO), 159.8 (**C-2**), 154.0 (NH**C**OO), 152.5 (**C-6**), 147.2 (**C-4**), 105.2 (**C**Cl$_3$), 90.3 (**C-5**), 89.2 (**C-4'**), 88.7 (**C-1'**), 71.6 (**C-3'**), 62.7 (**C-5'**), 42.9 (**C-2'**), 26.1 (Si-C(**C**H$_3$)$_3$), 25.9 (Si-C(**C**H$_3$)$_3$), 21.6 (C(**C**H$_3$)), 21.5 (C(**C**H$_3$)) 18.6 (Si-**C**(CH$_3$)$_3$), 18.1 (Si-**C**(CH$_3$)$_3$), -4.4 (Si-**C**H$_3$), -4.8 (Si-**C**H$_3$), -5.1 (Si-**C**H$_3$), -5.2 (Si-**C**H$_3$). **C**(CH$_3$)$_2$ is not observed.

HR-MS: (ESI positive, CHCl$_3$/MeOH), $[M+H]^+$ calcd for $C_{27}H_{47}Cl_3N_3O_7Si_2$: 686.20126, found: 686.20085.

Melting range: 46 °C - 48 °C.

Optical rotation: $[\alpha]_D^{24}(\text{deg cm}^3\ \text{g}^{-1}\ \text{dm}^{-1}) = +26.8$ (c = 1.0, CHCl$_3$).

3',5'-(*tert*-Butyldimethylsilyl)-*N*^{4}, *N*^{4}-bis(2,2,2-trichloro-*tert*-butyloxycarbonyl)-5-formyl-2'-deoxycytidine (5)

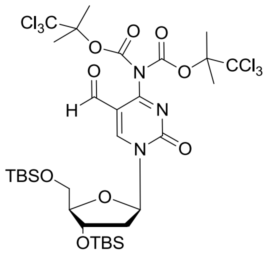

5

To a solution of aldehyde **1** (1.50 g, 3.10 mmol, 1.0 equiv) in DCM (44.0 mL) and pyridine (30.0 mL) a solution of TCBocCl (5.22 g, 21.7 mmol, 7.0 equiv) in DCM (12.0 mL) was added dropwise at room temperature. After stirring at this temperature overnight the reaction mixture was poured into ice. After phase separation, the aqueous layer was extracted with DCM (3 × 30 mL). The combined organic phases were washed with brine and dried over MgSO$_4$. The solvents were removed under reduced pressure. The crude product was purified by column chromatography (Hex/EtOAc, 20:1 → 1:1 *v/v*) to yield **5** (1.39 g, 1.56 mmol, 50%) as a yellow solid.

R$_f$: 0.61 (Hex/EtOAc = 2:1 *v/v*).

IR: (KBr) ν_{max} = 3441, 2954, 1819, 1686, 1520, 1389, 1289, 1110, 838, 792 cm^{-1}.

^{1}H-NMR: (400 MHz, CDCl$_3$) δ (ppm) 9.62 (s, 1H, C**H**O), 8.90 (s, 1H, **H-6**), 6.19 (dd, *J* = 5.8, 5.8 Hz, 1H, **H-1'**), 4.37-4.41 (m, 1H, **H-3'**), 4.06-4.08 (m, 1H, **H-4'**), 3.99 (dd, *J* = 11.6, 2.9 Hz, 1H, **H-5'**), 3.81 (dd, *J* = 11.6, 2.9 Hz, 1H, **H-5'**), 2.62-2.68 (m, 1H, **H-2'**), 2.03-2.09 (m, 1H, **H-2'**), 1.92-1.91 (m, 12H, 2xC(C**H**$_3$)$_2$, 0.92 (s, 9H, Si-C(C**H$_3$**)$_3$, 0.90 (s, 9H, Si-C(C**H$_3$**)$_3$, 0.12 (s, 3H, Si-C**H$_3$**),0.11 (s, 9H, 3xSi-C**H$_3$**);

^{13}C-NMR: (100 MHz, $CDCl_3$) δ (ppm) 183.3 (**C**HO), 160.9 (**C-2**), 153.6 (N**C**OO), 152.0 (**C-6**), 146.1 (**C-4**), 113.5, 105.4 (**C**Cl_3), 91.7, 88.8 (**C-4'**), 88.3 (**C-1'**), 70.3 (**C-3'**), 62.1 (**C-5'**), 42.4 (**C-2'**), 26.2 (Si-C(**C**H_3)$_3$), 25.8 (Si-C(**C**H_3)$_3$), 21.39 (C(**C**H_3)$_2$), 21.36 (C(**C**H_3)$_2$), 18.7 (Si-**C**(CH_3)$_3$), 18.1 (Si-**C**(CH_3)$_3$), -4.4 (Si-**C**H_3), -4.7 (Si-**C**H_3), -4.9 (Si-**C**H_3), -5.2 (Si-**C**H_3).

HR-MS: (ESI positive, $CHCl_3$/MeOH), $[M+Na]^+$ calcd for $C_{32}H_{51}Cl_6N_3O_9Si_2Na$: 910.11872, found: 910.11808.

Melting range: 64 °C-66 °C.

Optical rotation: $[\alpha]_D^{24}(\deg\ cm^3\ g^{-1}\ dm^{-1}) = +31.6$ (c = 1.0, $CHCl_3$).

3',5'-Di(*tert*-butyldimethylsilyl)-*N*^{4}-(2,2,2-trichloro-*tert*-butyloxycarbonyl)-5-(1-hydroxyethyl)-2'-deoxycytidine (6a) and 6-((2*R*,4*S*,5*R*)-4-(*tert*-butyldimethylsilyloxy)-5-((*tert*-butyldimethylsilyloxy)methyl)tetrahydrofuran-2-yl)-4-methyl-1*H*-pyrimido[4,5-*d*][1,3]oxazine-2,7(4*H*,6*H*)-dione (7a)

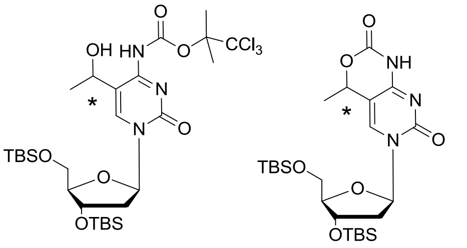

6a **7a**

To an ice-cooled solution of aldehyde **4** (81.0 mg, 118 µmol, 1.0 equiv) in THF (3.00 mL) methylmagnesium bromide (3.0 M in Et_2O; 78.0 µL, 236 µmol, 2.0 equiv) was added dropwise and the solution was allowed to warm to room temperature. After one hour the reaction mixture was cooled down to 0 °C and an additional amount of methylmagnesium bromide (3.0 M in Et_2O; 78.0 µL, 236 µmol, 2.0 equiv) was added dropwise. The reaction mixture was stirred overnight at room temperature. Afterwards the reaction was quenched with saturated aqueous NH_4Cl (5 mL) and diluted with DCM (5 mL). The phases were separated and the aqueous layer was extracted with DCM (2 × 5 mL). The combined organic layers were washed with brine (10 mL), dried over $MgSO_4$ and the solvents were removed under reduced pressure. The crude product was purified by column chromatography (Hex/EtOAc, 20:1 → 2:1 *v/v* and than DCM/MeOH, 30:1 → 15:1 *v/v*) to yield a mixture of diastereomeric alcohols **6a** (23 mg, 32.7 µmol, 28%) as yellow oil (a ratio could not be calculated) and a mixture of diastereomers **7a** with a ratio of 1.9:1 (22 mg, 41.8 µmol, 35%) as a slight yellow oil.

6a:

R_f: 0.57 (Hex/EtOAc = 3:1 *v/v*).

Due to spontaneous cyclisation of **6a** to **7a** ^{1}H-NMR spectrum of **6a** contains compound **7a**.

^{1}H-NMR: (300 MHz, $CDCl_3$) δ (ppm) 12.05 (s, 2H), 7.60 (s, 1H, **H-6**), 7.59 (s, 1H, **H-6***), 6.28-6.21 (m, 2H, **H-1'**, **H-1'***), 4.72-4.67 (m, 2H, C**H**-CH_3, C**H***-CH_3), 4.40-4.36 (m, 2H,

H-3', **H-3'***), 4.00-3.97 (m, 2H, **H-4'**, **H-4'***), 3.81-3.77 (m, 4H, **H-5'**, **H-5'***), 2.39-2.30 (m, 2H, **H-2'**, **H-2'***), 2.07-1.95 (m, 14H, **H-2'**, **H-2'***, C(C**H**$_3$)$_2$, C(C**H**$_3$)$_2$*), 1.50-1.46 (m, 3H, CH-C**H$_3$**, CH-C**H$_3$***), 0.91 (s, 18H, Si-C(C**H$_3$**)$_3$, Si-C(C**H$_3$**)$_3$*), 0.89 (s, 18H, Si-C(C**H$_3$**)$_3$, Si-C(C**H$_3$**)$_3$*), 0.10 (s, 12H, Si-(C**H$_3$**)$_2$, Si-(C**H$_3$**)$_2$*), 0.08-0.07 (m, 12H, Si-(C**H$_3$**)$_2$, Si-(C**H$_3$**)$_2$*).

HR-MS: (ESI positive, $CHCl_3$/MeOH), $[M+H]^+$ calcd for $C_{28}H_{51}Cl_3N_3O_7Si_2$: 702.23256, found: 702.23273.

7a:

R$_f$: 0.24 (DCM/MeOH = 30:1 *v/v*).

IR: (CCl_4) ν_{max} = 2929, 2857, 1758, 1669, 1561, 1489, 1362, 1254, 1077, 837, 780, 668 cm^{-1}.

^{1}H-NMR: (300 MHz, $CDCl_3$) δ (ppm) 8.38 (bs, 2H, N**H,** N**H***), 8.00 (d, *J* = 1.1 Hz, 1H, **H-6**), 7.94 (d, *J* = 1.2 Hz, 1H, **H-6***), 6.24-6.17 (m, 2H, **H-1'**, **H-1'***), 5.36-5.28 (m, 2H, C**H**-CH$_3$, C**H***-CH$_3$), 4.37-4.30 (m, 2H, **C-3'**, **C-3'***), 4.08-3.99 (m, 2H, **C-4'**, **C-4'***), 3.93-3.84 (m, 2H, **C-5'**, **C-5'***), 3.78-3.73 (m, 2H, **C-5'**, **C-5'***), 2.64-2.56 (m, 2H, **C-2'**, **C-2'***), 2.03-1.89 (m, 2H, **C-2'**, **C-2'***), 1.65 (d, *J* = 1.0 Hz, 3H, CH-C**H**$_3$), 1.62 (d, *J* = 0.8 Hz, 3H, CH-C**H$_3$***), 0.90-0.88 (m, 36H, 2xSi-C(C**H$_3$**)$_3$, 2xSi-C(C**H$_3$**)$_3$*), 0.09-0.06 (m, 24H, 2xSi-(C**H$_3$**)$_2$, 2xSi-(C**H$_3$**)$_2$*).

^{13}C-NMR: (75 MHz, $CDCl_3$) δ (ppm) 159.1 (158.9) (**C-4**), 154.6 (**C-2**), 149.8 (149.6) (NH**C**OO), 138.4 (138.1) (**C-6**), 101.6 (101.3) (**C-5**), 88.8 (88.7) (**C-4'**), 88.0 (87.7) (**C-1'**), 72.9 (72.7), 72.1 (72.0), 63.0 (62.9) (**C-5'**), 42.8 (42.7) (**C-2'**), 26.1 (26.0) (Si-C(**C**H$_3$)$_3$), 25.9 (Si-C(**C**H$_3$)$_3$), 20.9 (20.3) (CH-**C**H$_3$), 18.6 (18.50) (Si-**C**(CH$_3$)$_3$), 18.14 (18.12) (Si-**C**(CH$_3$)$_3$), -4.4 (-4.5) (Si-**C**H$_3$), -4.8 (Si-**C**H$_3$), -5.1 (-5.2) (Si-**C**H$_3$), -5.3 (Si-**C**H$_3$).

HR-MS: (ESI positive, MeOH), $[M+Na]^+$ calcd for $C_{24}H_{43}N_3O_6Si_2Na$: 548.25826, found: 548.25858, $[2M+Na]^+$ calcd for $C_{48}H_{86}N_6O_{12}Si_4Na$: 1073.52730, found: 1073.52735.

3',5'-Di(*tert*-butyldimethylsilyl)-*N*^{4}-(2,2,2-trichloro-*tert*-butyloxycarbonyl)-5-(1-hydroxy-3-(trimethylsilyl)prop-2-ynyl)-2'-deoxycytidine (6b) and 6-((2*R*,4*S*,5*R*)-4-(*tert*-butyldimethylsilyloxy)-5-((*tert*-butyldimethylsilyloxy)methyl)tetrahydrofuran-2-yl)-4-((trimethylsilyl)ethynyl)-1*H*-pyrimido[4,5-*d*][1,3]oxazine-2,7(4*H*,6*H*)-dione (7b)

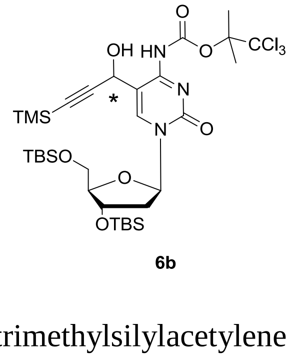

6b

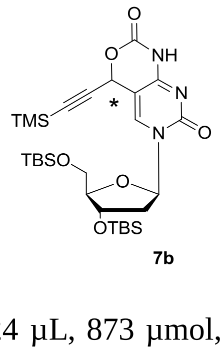

7b

To a stirred solution of trimethylsilylacetylene (124 µL, 873 µmol, 4.0 equiv) in THF (1.50 mL) *n*-BuLi (2.5 M in *n*-hexane; 349 µL, 873 µmol, 4.0 equiv) was added dropwise at −40 °C. The reaction mixture was stirred at this temperature for one hour. After the reaction mixture was cooled down to −60 °C a solution of aldehyde **4** (150 mg, 218 µmol, 1.0 equiv) in THF

(1.50 mL) was added dropwise. The reaction mixture was stirred at −60 °C for two and a half hours and thereafter at −50 °C for 30 min. The reaction was quenched with saturated aqueous NH_4Cl (5 mL), diluted with DCM (5 mL) and the aqueous layer was extracted with DCM (3 × 5 mL). The combined organic phases were washed with brine, dried over $MgSO_4$ and the solvents were removed under reduced pressure. Purification by silica gel chromatography (Hex/EtOAc, 20:1 → 1:1 *v/v* and than DCM/MeOH, 30:1 → 15:1 *v/v*) yielded a mixture of diastereomeric alcohols **6b** with a ratio of 2:1 (72.0 mg, 91.7 µmol, 42%) as a yellow oil and a mixture of diastereomers **7b** with a ratio of 1.1:1 (40.0 mg, 65.8 µmol, 30%) as a yellow oil.

6b:

R_f: 0.55 (Hex/EtOAc = 3:1 *v/v*).

IR: (CCl_4) ν_{max} = 3337, 2955, 2858, 1769, 1666, 1564, 1472, 1371, 1253, 1030, 840, 786 cm^{-1}.

^{1}H-NMR: (400 MHz, $CDCl_3$) δ (ppm) 12.02 (bs, 2H), 7.94 (s, 1H, **H-6**), 7.90 (s, 1H, **H-6***), 6.19-6.12 (m, 2H, **H-1’**, **H-1’***), 5.38 (s, 1H, C**H**-OH), 5.31(s, 1H, C**H***-OH), 4.37-4.35 (m, 2H, **H-3’**, **H-3’***), 4.05-4.01 (m, 2H, **H-4’**, **H-4’***), 3.80-3.71 (m, 4H, **H-5’**, **H-5’***), 2.46-2.43 (m, 2H, **H-2’**, **H-2’***), 2.09-2.01 (m, 2H, **H-2’**, **H-2’***), 1.95 (s, 12H, C(C**H**$_3$)$_2$, C(C**H**$_3$)$_2$*), 0.90 (s, 36H, 2xSi-C(C**H$_3$**)$_3$, 2xSi-C(C**H$_3$**)$_3$*), 0.19 (s, 18H, Si-(C**H$_3$**)$_3$, Si-(C**H$_3$**)$_3$*), 0.09-0.08 (m, 24H, 2xSi-(C**H$_3$**)$_2$, 2xSi-(C**H$_3$**)$_2$*).

In ^{13}C-NMR spectrum of **6b** signals of **7b** could be observed due to spontaneous cyclisation of diastereomeric alcohols **6b**.

^{13}C-NMR: (100 MHz, $CDCl_3$) δ (ppm) 159.6, 147.2, 138.3, 112.7, 106.3, 102.5, 97.7, 96.9, 92.1, 88.8, 87.4, 73.12 (72.8), 63.5, 60.5, 41.7, 26.0, 25.9, 21.43, 21.40, 18.5, 18.1, 0.01, -4.50, -4.69, -5.14, -5.18.

HR-MS: (ESI positive, $CHCl_3$/MeOH), $[M+H]^+$ calcd for $C_{32}H_{57}Cl_3N_3O_7Si_3$: 784.25644, found: 784.25647, $[M+Na]^+$ calcd for $C_{32}H_{56}Cl_3N_3O_7Si_3Na$: 806.23838, found: 806.23818.

7b:

R_f: 0.27 (DCM/MeOH = 30:1 *v/v*).

IR: (CCl_4) ν_{max} =3186, 2929, 2857, 1766, 1673, 1564, 1491, 1362, 1316, 1252, 1058, 840, 780, 669 cm^{-1}.

^{1}H-NMR: (300 MHz, $CDCl_3$) δ (ppm) 8.22 (s, 1H, **H-6**), 8.19 (s, 1H, **H-6***), 6.21-6.14 (m, 2H, **H-1’**, **H-1’***), 5.81 (d, *J* = 1.0 Hz, 1H, C**H***-C≡C-TMS), 5.80 (d, *J* = 0.9 Hz, 1H, C**H***-C≡C-TMS), 4.57 (bs, 2H), 4.35-4.32 (m, 2H, **H-3’**, **H-3’***), 4.10-4.07 (m, 1H, **H-4’**), 4.10-4.03 (m, 1H, **H-4’***), 3.89-3.84 (m, 2H, **H-5’**, **H-5’***), 3.80-3.74 (m, 2H, **H-5’**, **H-5’***), 2.73-2.56 (m, 2H, **H-2’**, **H-2’***), 2.08-1.98 (m, 2H, **H-2’**, **H-2’***), 0.91-0.88 (m, 36H, 2xSi-C(C**H$_3$**)$_3$, 2xSi-C(C**H$_3$**)$_3$*), 0.20 (s, 9H, Si-(C**H**$_3$)$_3$), 0.19 (s, 9H, Si-(C**H**$_3$)$_3$*), 0.09-0.07 (m, 24H, 2xSi-(C**H$_3$**)$_2$, 2xSi-(C**H$_3$**)$_2$*).

^{13}C-NMR: (75 MHz, $CDCl_3$) δ (ppm) 158.7 (**C-4**), 154.6 (**C-2**), 148.7 (NH**C**OO), 140.1 (**C-6**), 97.9 (**C-5**), 97.0 (**C**≡C-TMS), 96.5 (C≡**C**-TMS), 89.1 (**C-4’**), 88.6 (**C-1’**), 72.3 (72.0) (**C-3’**), 66.2 (65.9) (**C**H-C≡C-TMS), 63.0 (**C-5’**), 42.8 (C-2’), 26.2 (26.1) (Si-C(**C**H$_3$)$_3$), 26.0

(25.9) (Si-C(**C**H_3)$_3$), 18.4 (18.1) (Si-**C**(CH_3)$_3$), - 0.029 (-0.351) (Si-(**C**H_3)$_3$), -4.4 (Si-**C**H_3), -4.8 (Si-**C**H_3), -5.2 (Si-**C**H_3), -5.3 (Si-**C**H_3).

HR-MS: (ESI positive, MeOH), $[M+Na]^+$ calcd for $C_{28}H_{49}N_3O_6Si_3Na$: 630,28269, found: 630.28187.

3',5'-Di(*tert*-butyldimethylsilyl)-*N*^{4}-(2,2,2-trichloro-*tert*-butyloxycarbonyl)-5-(1-hydroxy-3-phenylprop-2-ynyl)-2'-deoxycytidine (6c) and 6-((2*R*,4*S*,5*R*)-4-(*tert*-butyldimethylsilyloxy)-5-((*tert*-butyldimethylsilyloxy)methyl)tetrahydrofuran-2-yl)-4-(phenylethynyl)-1*H*-pyrimido[4,5-*d*][1,3]oxazine-2,7(4*H*,6*H*)-dione (7c)

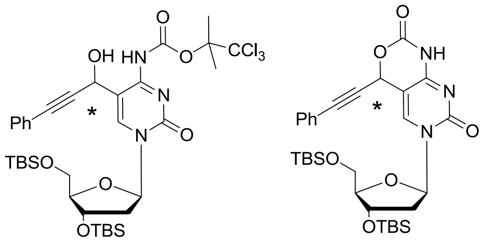

To a stirred solution of phenylacetylene (77.0 µL, 698 µmol, 4.0 equiv) in THF (1.50 mL) at −85 °C 1.89 ml of *n*-BuLi (2.5 M in *n*-hexane; 279 µL, 698 µmol, 4.0 equiv) was added dropwise. Subsequently the temperature was increased to −40 °C in the course of 1 hour and lowered to -78 °C. A solution of aldehyde **4** (120 mg, 175 µmol, 1.0 equiv) in THF (3.00 mL) was added dropwise and this mixture stirred for one hour. The reaction mixture was quenched with saturated aqueous NH_4Cl (5 mL), diluted with DCM (5 mL) and the aqueous layer was extracted with DCM (3 × 5 mL). The combined organic phases were washed with brine (10 mL), dried over $MgSO_4$ and the solvents were rotary evaporated. Purification by silica gel chromatography (Hex/EtOAc, 20:1 → 2:1 *v/v* and than DCM/MeOH, 30:1 → 15:1 *v/v*) yielded a mixture of diastereomeric alcohols **6c** with a ratio of 1.6:1 (58.0 mg, 73.5 µmol, 47% brsm) as a yellow oil and a mixture of diastereomers **7c** with a ratio of 1.4:1 (32.0 mg, 52.3 µmol, 34% brsm) as an orange solid. And 14.0 mg (2.04 µmol, 12%) of recovered starting material.

6c:

R_f: 0.18 (Hex/EtOAc = 4:1 *v/v*)

IR: (CCl_4) ν_{max} = 3390, 2929, 1770, 1664, 1562, 1255, 1148, 1028, 837, 781 cm^{-1}.

^{1}H-NMR: (300 MHz, $CDCl_3$) δ (ppm) 12.04 (bs, 2H), 8.01 (s, 2H, **H-6**, **H-6***), 7.49-7.42 (m, 4H, C**H** aromat., C**H*** aromat.), 7.37-7.28 (m, 6H, C**H** aromat., C**H*** aromat.), 6.23-6.15 (m, 2H, **H-1', H-1'***), 5.61 (s, 1H, C**H**-C≡C-Ph), 5.57 (s, 1H, C**H***-C≡C-Ph) 4.37-4.33 (m, 2H, **H-3', H-3'***), 4.03-3.99 (m, 2H, **H-4', H-4'***), 3.73-3.58 (m, 4H, **H-5', H-5'***), 2.50-2.40 (m, 2H, **H-2'**, **H-2'***), 2.06-1.99 (m, 2H, **H-2'**, **H-2'***), 1.96 (s, 12H, C(C**H$_3$**)$_2$, C(C**H$_3$**)$_2$*), 0.89 (s, 18H, Si-C(C**H$_3$**)$_3$, Si-C(C**H$_3$**)$_3$*), 0.85-0.84 (m, 18H, Si-C(C**H$_3$**)$_3$, Si-C(C**H$_3$**)$_3$*), 0.07-0.06 (m, 12H, Si-(C**H$_3$**)$_2$, Si-(C**H$_3$**)$_2$*), 0.00-(-0.04) (m, 12H, Si-(C**H$_3$**)$_2$, Si-(C**H$_3$**)$_2$*).

^{13}C-NMR: (100 MHz, $CDCl_3$) δ (ppm) 159.7, 147.2, 137.9, 131.9 131.8, 128.92, 128.88, 128.50, 128.47, 122.3, 113.1, 106.3, 89.1, 88.84, 88.75, 87.3, 87.2, 86.9, 86.2, 73.0 (72.9),

63.4 (63.3), 60.6 (60.4), 41.8 (41.6), 26.02 (26.0), 25.91, 25.85, 21.44 (21.42), 18.43 (18.42), 18.10 (18.09), -4.55, -4.73, -5.30, -5.42.

HR-MS: (ESI positive, MeOH), $[M+Na]^+$ calcd for $C_{35}H_{52}Cl_3N_3O_7Si_2Na$: 810.23071, found: 810.22997.

7c:

R$_f$: 0.24 (DCM/MeOH = 30:1 *v/v*).

IR: (CCl_4) ν_{max} = 2928, 2857, 1765, 1669, 1564, 1491, 1254, 1093, 1031, 837, 782, 603 cm^{-1}.

^{1}H-NMR: (300 MHz, $CDCl_3$) δ (ppm) 8.31 (s, 1H, **H-6**), 8.26 (s, 1H, **H-6***), 7.45 (m, 4H, C**H** aromat., C**H*** aromat.), 7.41-7.30 (m, 6H, C**H** aromat., C**H*** aromat.), 6.23-6.18 (m, 2H, **H-1'**, **H-1'***), 6.09 -6.07 (m, 2H, C**H**-C≡C-Ph, C**H***-C≡C-Ph), 4.35-4.32 (m, 2H, **H-3'**, **H-3'***), 4.07-4.04 (m, 2H, **H-4'**, **H-4'***), 3.88-3.80 (m, 2H, **H-5'**, **H-5'***), 3.76-3.71 (m, 2H, **H-5'**, **H-5'***), 2.69-2.59 (m, 2H, **H-2'**, **H-2'***), 2.08-1.99 (m, 2H, **H-2'**, **H-2'***), 0.88-0.87 (m, 18H, Si-C(C**H$_3$**)$_3$, Si-C(C**H$_3$**)$_3$*), 0.86-0.85 (m, 18H, Si-C(C**H$_3$**)$_3$, Si-C(C**H$_3$**)$_3$*), 0.08-0.05 (m, 12H, Si-(C**H$_3$**)$_2$, Si-(C**H$_3$**)$_2$*), 0.03-0.01 (m, 12H, Si-(C**H$_3$**)$_2$, Si-(C**H$_3$**)$_2$*).

^{13}C-NMR: (100 MHz, $CDCl_3$) δ (ppm) 158.8 (**C-4**), 155.0 (**C-2**), 148.8 (NH**C**OO), 140.1 (139.7) (**C-6**), 132.3 (132.1) (**C** aromat.), 130.0 (129.9) (**C** aromat.), 128.7 (**C** aromat.), 120.7 (**C** aromat.), 98.2 (**C-5**), 90.2 (90.0), 89.1 (88.8), 88.5 (88.2), 81.7 (81.5), 72.2 (**C-3'**), 66.5 (66.3) (**C**H-C≡C-Ph), 63.0 (62.9) (**C-5'**), 42.8 (42.6) (**C-2'**), 26.1 (26.0) Si-C(**C**H$_3$)$_3$, 26.0 (25.9) (Si-C(**C**H$_3$)$_3$), 18.5 (18.4) (Si-**C**(CH$_3$)$_3$), 18.1 (Si-**C**(CH$_3$)$_3$), -4.4 (-4. 5) (Si-**C**H$_3$), -4.76 (-4.78) (Si-**C**H$_3$), -5.3 (Si-**C**H$_3$), -5.35 (-5.37) (Si-**C**H$_3$).

HR-MS: (ESI positive, MeOH), $[M+Na]^+$ calcd for $C_{31}H_{45}N_3O_6Si_2Na$: 634.27391, found: 634.27421, $[2M+Na]^+$ calcd for $C_{62}H_{90}N_6O_{12}Si_4Na$: 1245.55860, found: 1245.55822.

6-((2*R*,4*S*,5*R*)-4-(*tert*-Butyldimethylsilyloxy)-5-((*tert*-butyldimethylsilyloxy)methyl)tetrahydrofuran-2-yl)-4-vinyl-1*H*-pyrimido[4,5-*d*][1,3]oxazine-2,7(4*H*,6*H*)-dione (7d)

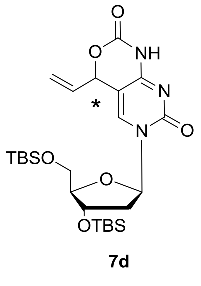

7d

To an ice-cooled solution of aldehyde **4** (182 mg, 265 µmol, 1.0 equiv) in THF (3.00 mL) vinylmagnesium bromide (0.7 M in THF; 757 µl, 530 µmol, 2.0 equiv) was added dropwise and the solution was stirred at 0 °C for 30 min. The reaction mixture was allowed to warm to room temperature whereby it was stirred for three hours. The reaction was quenched with saturated aqueous NH_4Cl (5 mL) and diluted with Et_2O (5 mL). After phase separation the aqueous layer was extracted with Et_2O (2 × 5 mL). The combined organic layers were washed with brine (10 mL), dried over $MgSO_4$ and the solvents were removed under reduced

pressure. The crude product was purified by column chromatography (DCM/MeOH, 30:1 → 15:1 *v/v*) to yield a mixture of diastereomers **7d** with a ratio of 2.3:1 (98.0 mg, 182 µmol, 69%) as a slight yellow oil.

7d:

R_f: 0.27 (DCM/MeOH = 30:1 *v/v*).

IR: (KBr) ν_{max} = 3428, 2954, 2858, 1759, 1663, 1567, 1384, 1254, 1093, 838, 780 cm^{-1}.

^{1}H-NMR: (400 MHz, DMSO) δ (ppm) 11.31 (bs, 2H, N**H,** N**H***), 7.84 (s, 1H, **H-6**), 7.80 (s, 1H, **H-6***), 6.09-5.94 (m, 4H, **H-1'**, **H-1'***, C**H**=CH_2, C**H***=CH_2), 5.84 (d, *J* = 6.0 Hz, 1H, C**H***-CH=CH_2), 5.80 (d, *J* = 6.1 Hz, 1H, C**H**-CH=CH_2), 5.41-5.34 (m, 4H, CH=C**H_2**, CH=C**H_2***), 4.34-4.30 (m, 2H, **H-3'**, **H-3'***), 3.93-3.89 (m, 2H, **H-4'**, **H-4'***), 3.77-3.65 (m, 4H, **H-5'**, **H-5'***), 2.34-2.27 (m, 2H, **H-2'**, **H-2'***), 2.16-2.08 (m, 2H, **H-2'**, **H-2'***), 0.87 (s, 18H, Si-C(C**H_3**)$_3$, Si-C(C**H_3**)$_3$*), 0.85 0.84 (m, 18H, Si-C(C**H_3**)$_3$, Si-C(C**H_3**)$_3$*), 0.07 (s, 12H, Si-(C**H_3**)$_2$, Si-(C**H_3**)$_2$*), 0.04 (s, 12H, Si-(C**H_3**)$_2$, Si-(C**H_3**)$_2$*).

^{13}C-NMR: (100 MHz, DMSO) δ (ppm) 159.4 (159.3) (**C-4**), 154.0 (**C-2**), 149.9 (149.8) (NH-**C**OO), 139.4 (139.0) (**C**H=CH_2), 134.1 (133.9) (**C-6**), 119.6 (119.4) (CH=**C**H_2), 98.8 (98.5) (**C-5**), 87.8 (87.7) (**C-4'**), 86.9 (86.7) (**C-1'**), 75.4, 75.3 (**C**H-CH=CH_2), 72.34 (72.30) (**C-3'**), 62.8, 62.7 (**C-5'**), 40.7 (40.5) (**C-2'**), 25.8 (25.7) (Si-C(**C**H_3)$_3$), 25.7 (Si-C(**C**H_3)$_3$), 18.0 (17.9) (Si-**C**(CH_3)$_3$), 17.7 (Si-**C**(CH_3)$_3$), -4.8 (Si-**C**H_3), -5.0 (Si-**C**H_3), -5.5 (Si-**C**H_3), -5.5 (Si-**C**H_3).

HR-MS: (ESI positive, DCM/MeOH), $[M+Na]^+$ calcd for $C_{25}H_{43}N_3O_6Si_2Na$: 560.25881, found: 560.25898, $[2M+Na]^+$ calcd for $C_{50}H_{86}N_6O_{12}Si_4Na$: 1097.52785 found: 1097.52679.

3',5'-Di(*tert*-butyldimethylsilyl)-N^4-(2,2,2-trichloro-*tert*-butyloxycarbonyl)-3,6-dihydro-5-formyl-6-methyl-2'-deoxycytidine (8a)

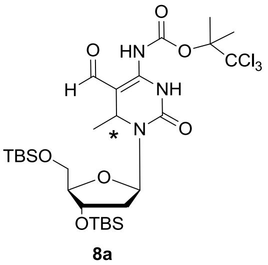

8a

To an ice-cooled solution of aldehyde **5** (80.0 mg, 89.8 µmol, 1.0 equiv) in THF (2.00 mL) methylmagnesium bromide (3.0 M in Et_2O; 60.0 µL, 180 µmol, 2.0 equiv) was added dropwise and the solution was stirred for three hours. The reaction mixture was then allowed to warm to room temperature whereby it was stirred for three hours. Subsequently it was cooled to 0 °C and an additional amount of methylmagnesium bromide (3.0 M in Et_2O; 30.0 µL, 90.0 µmol, 1.0 equiv) was added dropwise. The reaction mixture was stirred overnight at room temperature, then quenched with saturated aqueous NH_4Cl (5 mL) and diluted with DCM (5 mL). The phases were separated and the aqueous layer extracted with DCM (2 × 5 mL). The combined organic layers were washed with brine (10 mL), dried over $MgSO_4$ and the solvents were removed under reduced pressure. The crude product was

purified by column chromatography (Hex/EtOAc, 20:1 → 2:1 *v/v*) to yield a mixture of diastereomers **8a** (24.0 mg, 34.1 mmol, 38%) with a ratio of 2.4:1 as a yellow oil.

R_f: 0.23 (Hex/EtOAc = 10:1 *v/v*).

IR: (CCl_4) ν_{max} = 2929, 1705, 1598, 1387, 1254, 1146, 836, 785 cm^{-1}.

^{1}H-NMR: (300 MHz, $CDCl_3$) δ (ppm) 11.87 (bs, 2H, N**H**COO, N**H***COO), 9.59 (s, 1H, N**H**), 9.56 (s, 1H, N**H***), 9.07 (s, 1H, C**H**O), 9.06 (s, 1H, C**H**O*), 6.18 (dd, *J* = 8.6, 5.7 Hz, 1H, **H-1'**), 6.10-6.02 (m, 1H, **H-1'***), 4.62 (q, *J* = 6.6 Hz, 1H, **H-6***), 4.46 (q, *J* = 6.4 Hz, 1H, **H-6**), 4.40-4.34 (m, 2H, **H-3'**, **H-3'***), 3.86-3.82 (m, 1H, **H-4'**), 3.82-3.79 (m, 1H, **H-4'***), 3.74-3.70 (m, 4H, **H-5'**, **H-5'***), 2.22-2.15 (m, 1H, **H-2'***), 2.13-2.01 (m, 2H, **H-2'**, **H-2'***), 1.98-1.97 (m, 7H, **H-2',** C(C**H**$_3$), C(C**H**$_3$)*), 1.96 (s, 6H, C(C**H**$_3$), C(C**H**$_3$)*), 1.38 (d, *J* = 6.4 Hz, 3H, CH-C**H**$_3$), 1.33-1.28 (m, 3H, CH-C**H**$_3$*), 0.91 (s, 18H, Si-C(C**H**$_3$)$_3$, Si-C(C**H**$_3$)$_3$*), 0.89-0.88 (m, 18H, Si-C(C**H**$_3$)$_3$, Si-C(C**H**$_3$)$_3$*), 0.09-0.06 (m, 24H, 2xSi-(C**H**$_3$)$_2$, 2xSi-(C**H**$_3$)$_2$*).

^{13}C-NMR: (75 MHz, $CDCl_3$) δ (ppm) 186.5 (186.4) (**C**HO), 151.7 (NH**C**OO), 150.3 (**C-2**), 147.5 (**C-4**), 105.1 (**C**Cl$_3$), 96.3 (96.2) (**C-5**), 91.2 (**C**(CH$_3$)$_2$), 87.0 (86.4) (**C-4'**), 85.4 (85.41) (**C-1'**), 72.7 (72.3) (**C-3'**), 63.6 (63.3) (**C-5'**), 46.7 (46.3) (**C-6**), 38.9 (**C-2'**), 26.11 (26.09) (Si-C(**C**H$_3$)$_3$), 26.0 (25.9) (Si-C(**C**H$_3$)$_3$), 24.7 (CH-**C**H$_3$), 21.68 (21.66) (C(**C**H$_3$)$_2$), 21.64 (21.63) (C(**C**H$_3$)$_2$), 18.6 (Si-**C**(CH$_3$)$_3$), 18.2 (Si-**C**(CH$_3$)$_3$), -4.5 (Si-**C**H$_3$), -4.60 (-4.62) (Si-**C**H$_3$), -5.2 (Si-**C**H$_3$), -5.27 (-5.29) (Si-**C**H$_3$).

HR-MS: (ESI positive, $CHCl_3$/MeOH), $[M+H]^+$ calcd for $C_{28}H_{51}Cl_3N_3O_7Si_2$: 702.23256, found: 702.23296, $[M+Na]^+$ calcd for $C_{28}H_{50}Cl_3N_3O_7Si_2Na$: 724.21451, found: 724.21520.

3',5'-Di(*tert*-butyldimethylsilyl)-*N*^{4}-(2,2,2-trichloro-*tert*-butyloxycarbonyl)-3,6-dihydro-5-formyl-6-(trimethylsilylethynyl)-2'-deoxycytidine (8b)

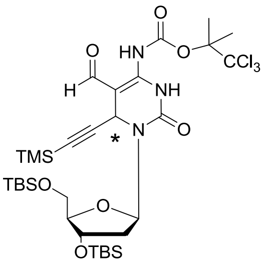

8b

To a stirred solution of trimethylsilylacetylene (51.1 µL, 359 µmol, 4.0 equiv) in THF (0.60 mL) at −40 °C, *n*-BuLi (2.5 M in *n*-hexane; 144 µL, 359 µmol, 4.0 equiv) was added dropwise. The temperature was increased to −20 °C whereby the reaction mixture was stirred for one hour. Afterwards the temperature was lowered again to −40 °C. A solution of aldehyde **5** (80.0 mg, 89.8 µmol, 1.0 equiv) in THF (1.50 mL) was added dropwise and the reaction mixture was stirred at −50 °C for three hours. The reaction mixture was quenched with saturated aqueous NH_4Cl (3 mL), diluted with DCM (5 mL) and the aqueous layer extracted with DCM (3 × 5 mL). The combined organic phases were washed with brine (10 mL), dried over $MgSO_4$ and the solvents were removed under reduced pressure. Purification by chromatography (Hex/EtOAc 30:1 → 15:1 *v/v*) afforded diastereomer **8b**

(42.9 mg, 54.6 µmol, 61%) as a yellow oil and diastereomer **8b*** (13.6 mg, 17.3 µmol, 19%) as a yellow oil. The total yield of the reaction is 80 % (56.5 mg, 91.7 µmol).

diastereomer **8b**

R_f: 0.68 (Hex/EtOAc = 5:1 *v/v*).

IR: (CCl_4) ν_{max} = 2926, 1715, 1666, 1603, 1463, 1252, 1140, 1030, 839, 777, 597 cm^{-1}.

^{1}H-NMR: (400 MHz, $CDCl_3$) δ (ppm) 11.86 (s, 1H, N**H**COO), 9.65 (s, 1H, N**H**), 9.13 (s, 1H, C**H**O), 5.87 (dd, *J* = 7.8, 5.7 Hz, 1H, **H-1'**), 5.21 (s, 1H, **H-6**), 4.38– 4.35 (m, 1H, **H-3'**), 3.92– 3.89 (m, 1H, **H-4'**), 3.74-3.63 (m, 2H, **H-5'**), 2.28-2.21 (m, 1H, **H-2'**), 2.14-2.09 (m, 1H, **H-2'**), 1.98 (s, 3H, C(C**H**$_3$)$_2$), 1.96 (s, 3H, C(C**H**$_3$)$_2$), 0.91 (s, 9H, Si-C(C**H$_3$**)$_3$), 0.89 (s, 9H, Si-C(C**H$_3$**)$_3$), 0.13 (s, 9H, Si-(C**H**$_3$)$_3$), 0.07 (m, 12H, 2 x Si-(C**H**$_3$)$_2$).

^{13}C-NMR: (100 MHz, $CDCl_3$) δ (ppm) 186.4 (**C**HO), 151.6 (NH**C**OO), 149.7 (**C-2**), 147.9 (**C-4**), 105.1 (**C**Cl$_3$), 103.2 (**C**≡C-TMS), 92.4 (**C-5**), 91.4 (**C**(CH$_3$)$_2$), 88.6 (C≡**C**-TMS), 87.7 (**C-4'**), 87.0 (**C-1'**), 73.0 (**C-3'**), 63.8 (**C-5'**), 43.5 (**C-6**), 39.5 (**C-2'**), 26.1 (Si-C(**C**H$_3$)$_3$), 25.9 (Si-C(**C**H$_3$)$_3$), 21.6 (C(**C**H$_3$)$_2$), 18.6 (Si-**C**(CH$_3$)$_3$), 18.1 (Si-**C**(CH$_3$)$_3$), -0.01 (Si-(**C**H$_3$)$_3$), -4.5 (Si-**C**H$_3$), -4.6 (Si-**C**H$_3$), -5.1 (Si-**C**H$_3$), -5.1 (Si-**C**H$_3$).

HR-MS: (ESI positive, DCM/MeOH), $[M+Na]^+$ calcd for $C_{32}H_{56}Cl_3N_3O_7Si_3Na$: 806.23838, found: 806.23863.

Optical rotation: $[\alpha]_D^{25}(\text{deg cm}^3\ \text{g}^{-1}\ \text{dm}^{-1}) = +6.0$ (c = 1.0, $CHCl_3$).

diastereomer **8b*:**

R_f: 0.60 (Hex/EtOAc = 5:1 *v/v*).

IR: (CCl_4) ν_{max} =3326, 2928, 1712, 1667, 1602, 1463, 1386, 1252, 1141, 1027, 839, 778 cm^{-1}.

^{1}H-NMR: (400 MHz, $CDCl_3$) δ (ppm) 11.84 (s, 1H, N**H***COO), 9.66 (s, 1H, N**H***), 9.10 (s, 1H, C**H**O*), 6.23 (dd, *J* = 8.3, 5.9 Hz, 1H, **H-1'***), 5.38 (s, 1H, **H-6***), 4.41-4.38 (m, 1H, **H-4'***), 3.85-3.67 (m, 3H, **H-3'***, **H-5'***), 2.58-2.51 (m, 1H, **H-2'***), 2.06-2.01 (m, 1H, **H-2'***), 1.99 (s, 3H, C(C**H**$_3$)$_2$*), 1.97 (s, 3H, C(C**H**$_3$)$_2$*), 0.93 (s, 9H, Si-C(C**H$_3$**)$_3$*), 0.90 (s, 9H, Si-C(C**H$_3$**)$_3$*), 0.13 (s, 9H, Si-(C**H**$_3$)$_3$*), 0.11 (s, 3H, Si-C**H$_3$***), 0.10 (s, 3H, Si-C**H$_3$***), 0.08 (m, 6H, Si-(C**H$_3$**)$_2$*).

^{13}C-NMR: (100 MHz, $CDCl_3$) δ (ppm) 186.5 (**C**HO), 151.7 (NH**C**OO), 149.5 (**C-2**), 148.0 (**C-4**), 105.1 (**C**Cl$_3$), 104.4 (**C**≡C-TMS), 92.5 (**C-5**), 91.4 (**C**(CH$_3$)$_2$), 89.1 (C≡**C**-TMS), 86.4 (**C-4'**), 84.4 (**C-1'**), 72.3 (**C-3'**), 63.3 (**C-5'**), 40.3 (**C-6**), 38.2 (**C-2'**), 26.2 (Si-C(**C**H$_3$)$_3$), 25.9 (Si-C(**C**H$_3$)$_3$), 21.7 (C(**C**H$_3$)$_2$), 18.7 (Si-**C**(CH$_3$)$_3$), 18.2 (Si-**C**(CH$_3$)$_3$), -0.1 (Si-(**C**H$_3$)$_3$), -4.4 (Si-**C**H$_3$), -4.6 (Si-**C**H$_3$), -5.1 (Si-**C**H$_3$), -5.2 (Si-**C**H$_3$).

HR-MS: (ESI positive, DCM/MeOH), $[M+Na]^+$ calcd for $C_{32}H_{56}Cl_3N_3O_7Si_3Na$: 806.23838, found: 806.23854.

Optical rotation: $[\alpha]_D^{22}(\text{deg cm}^3\ \text{g}^{-1}\ \text{dm}^{-1}) = +10.14$ (c = 0.46, $CHCl_3$).

3’,5’-Di(*tert*-butyldimethylsilyl)-N^4-(2,2,2-trichloro-*tert*-butyloxycarbonyl)-3,6-dihydro-5-formyl-6-(2-phenylethynyl)-2’-deoxycytidine (8c)

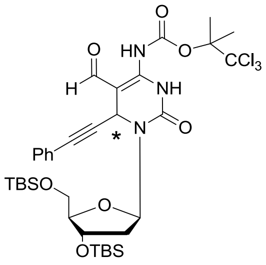

8c

To a stirred solution of phenylacetylene (49.3 µL, 449 µmol, 4.0 equiv) in THF (1.50 mL) at −78 °C n-BuLi (2.5 M in *n*-hexane; 180 µL, 449 µmol, 4.0 equiv) was added dropwise, whereby the reaction mixture was stirred for 80 min. Subsequently a solution of aldehyde **5** (100 mg, 112 µmol, 1.0 equiv) in THF (2.10 mL) was added dropwise. The temperature was increased to −50 °C, whereby the mixture was stirred for 140 min. The reaction mixture was quenched with saturated aqueous NH_4Cl (5 mL), diluted with DCM (5 mL) and the aqueous layer extracted with DCM (3 × 5 mL). The combined organic phases were washed with brine (10 mL), dried over $MgSO_4$ and the solvents were removed under reduced pressure. Purification by silica gel chromatography (Hex/EtOAc, 20:1 → 1:1 *v/v*) yielded a mixture of diastereomers **8c** with a ratio of 1.1:1 (63.0 mg, 79.8 µmol, 71%) as a yellow oil.

R_f: 0.26 (Hex/EtOAc = 10:1 *v/v*).

IR: (CCl_4) ν_{max} = 3323, 2929, 1710, 1601, 1470, 1389, 1256, 1208, 1141, 1028, 837, 784, 690 cm^{-1}.

^{1}H-NMR: (400 MHz, $CDCl_3$) δ (ppm) 11.88-11.87 (m, 2H, N**H**COO, N**H***COO), 9.75-9.74 (m, 2H, N**H**, N**H** *), 9.21 (s, 1H, C**H**O), 9.19 (s, 1H, C**HO***), 7.38-7.34 (m, 4H, C**H** aromat., C**H*** aromat.), 7.32-7.27 (m, 6H, C**H** aromat., C**H*** aromat.), 6.26 (dd, *J* = 7.8, 6.0 Hz, 1H, **H-1’***), 6.03 (dd, *J* = 7.9, 5.7 Hz, 1H, **H-1’**), 5.65 (s, 1H, **H-6***), 5.46 (s, 1H, **H-6**), 4.46-4.43 (m, 1H, **H-4’***), 4.39-4.38 (m, 1H, **H-3’**), 3.95-3.92 (m, 1H, **H-4’**), 3.86-3.82 (m, 2H, **H-5’**), 3.71-3.69 (m, 3H, **H-3’***, **H-5’***), 2.62-2.55 (m, 1H, **H-2’***), 2.27-2.20 (m, 1H, **H-2’**), 2.18-2.09 (m, 2H, **H-2’**, **H-2’***), 1.96-1.95 (m, 12H, C(C$\mathbf{H_3}$)$_2$, C(C$\mathbf{H_3}$)$_2$*), 0.92 (s, 6H, Si-C(C$\mathbf{H_3}$), Si-C(C$\mathbf{H_3}$)*), 0.89-0.88 (m, 24H, 2xSi-C(C$\mathbf{H_3}$), 2xSi-C(C$\mathbf{H_3}$)*), 0.86 (s, 6H, Si-C(C$\mathbf{H_3}$), Si-C(C$\mathbf{H_3}$)*), 0.12-0.11 (m, 6H, Si-C$\mathbf{H_3}$, Si-C$\mathbf{H_3}$*), 0.09-0.07 (m, 6H, Si-C$\mathbf{H_3}$, Si-C$\mathbf{H_3}$*), 0.05-0.04 (m, 6H, Si-C$\mathbf{H_3}$, Si-C$\mathbf{H_3}$*), 0.02 (s, 6H, Si-C$\mathbf{H_3}$, Si-C$\mathbf{H_3}$*).

^{13}C-NMR: (100 MHz, $CDCl_3$) δ (ppm) 186.4 (186.3) (**C**HO), 151.61 (151.58) (NH**C**OO), 150.0 (149.5) (**C-2**), 148.2 (148.0) (**C-4**), 131.9 (131.7) (**C** aromat.), 128.9 (128.7) (**C** aromat.), 128.5 (128.3) (**C** aromat.), 122.3 (122.0) (**C** aromat.), 105.0 (**C**Cl_3), 92.65 (92.62) (**C-5**), 91.4 (**C**(CH_3)$_2$), 88.2, 87.7, 87.4, 86.5, 86.4 , 84.4, 84.0, 83.6, 81.3, 72.8 (72.0) (**C-3’**), 63.7 (63.2) (**C-5’**), 42.9 (40.2) (**C-6**), 39.6 (38.6) (**C-2’**), 26.2 (26.1) (Si-C(**C**H_3)$_3$), 25.91 (25.88) (Si-C(**C**H_3)$_3$), 21.7 (21.6) (C(**C**H_3)$_2$), 21.64 (21.59) (C(**C**H_3)$_2$), 18.7 (18.1) (Si-**C**(CH_3)$_3$), 18.6 (18.1) (Si-**C**(CH_3)$_3$), -4.49 (-4.54) (Si-**C**H_3), -4.63 (-4.71) (Si-**C**H_3), -5.1 (-5.2) (Si-**C**H_3), -5.2 (-5.3) (Si-**C**H_3).

HR-MS: (ESI positive, DCM/MeOH), $[M+Na]^+$ calcd for $C_{35}H_{52}Cl_3N_3O_7Si_2Na$: 810.23016, found: 810.22984.

3',5'-Di(*tert*-butyldimethylsilyl)-*N*⁴-(2,2,2-trichloro-*tert*-butyloxycarbonyl)-3,6-dihydro-5-formyl-6-vinyl-2'-deoxycytidine (8d) and 3',5'-Di(*tert*-butyldimethylsilyl)-*N*⁴, *N*⁴-bis(2,2,2-trichloro-*tert*-butyloxycarbonyl)-3,6-dihydro-5-formyl-6-vinyl-2'-deoxycytidine (8e)

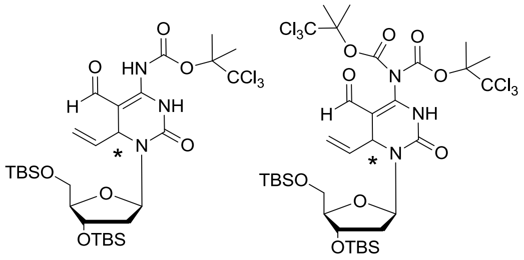

8d **8e**

To an ice-cooled solution of aldehyde **5** (100 mg, 112 µmol, 1.0 equiv) in THF (2.30 mL) vinylmagnesium bromide (0.7 M in THF; 320 µL, 225 µmol, 2.0 equiv) was added dropwise and the solution was stirred at 0 °C. After 135 min the reaction was quenched with saturated aqueous NH_4Cl (5 mL) and diluted with Et_2O (5 mL). The phases were separated and the aqueous layer was extracted with Et_2O (2 × 5 mL). The combined organic layers were washed with brine (5 mL), dried over $MgSO_4$ and the solvents were removed under reduced pressure. The crude product was purified by column chromatography (Hex/EtOAc, 20:1 → 1:1 *v/v*) to yield a mixture of diastereomers **8d** with a ratio of 2.6:1 (32.0 mg, 44.7 µmol, 37%) as a yellow oil and a mixture of diastereomers **8e** with a ratio of 5.7:1 (18.0 mg, 19.6 µmol, 17%) as a yellow oil.

8d:

R_f: 0.44 (Hex/EtOAc = 4:1 *v/v*).

IR: (CCl_4) ν_{max} = 3328, 2929, 2857, 1707, 1665, 1599, 1515, 1389, 1254, 1209, 1140, 1026, 836, 778 cm^{-1}.

^{1}H-NMR: (400 MHz, $CDCl_3$) δ (ppm) 11.94 (s, 1H, N**H**COO), 11.86 (s, 1H, N**H***COO), 9.58 (s, 2H, N**H**, N**H***), 9.07 (s, 1H, C**HO***), 9.03 (s, 1H, C**H**O), 6.23 (dd, *J* = 8.5, 5.8 Hz, 1H, **H-1'***), 6.09 (dd, *J* = 8.3, 5.6 Hz, 1H, **H-1'**), 6.04-5.91 (m, 1H, CH-C**H**=CH_2), 5.85-5.76 (m, 1H, CH-C**H***=CH_2), 5.16-5.12 (m, 2H, CH-CH=C**H**$_2$, CH-CH=C**H**$_2$*), 5.12-5.05 (m, 2H, CH-CH=C**H**$_2$, CH-CH=C**H**$_2$*), 5.04-5.02 (m, 1H, **H-6***), 4.90-4.89 (m, 1H, **H-6**), 4.37-4.30 (m, 2H, **H-3'**, **H-3'***), 3.86-3.84 (m, 1H, **H-4'**), 3.78-3.75 (m, 1H, **H-4'***), 3.73-3.72 (m, 2H, **H-5'***), 3.70-3.68 (m, 2H, **H-5'**), 2.19-2.10 (m, 2H, **H-2'**, **H-2'***), 2.03-1.99 (m, 1H, **H-2'**) 1.99-1.87 (m, 13H, **H-2'***, C(C**H**$_3$)$_2$, C(C**H**$_3$)$_2$*), 0.91-0.90 (m, 18H, Si-C(C**H**$_3$)$_3$, Si-C(C**H**$_3$)$_3$*), 0.88 (s, 18H, Si-C(C**H**$_3$)$_3$, Si-C(C**H**$_3$)$_3$*), 0.08 (s, 6H, Si-(C**H**$_3$)$_2$*), 0.07-0.05 (m, 18H, Si-(C**H**$_3$)$_2$*, 2xSi-(C**H**$_3$)$_2$).

^{13}C-NMR: (100 MHz, $CDCl_3$) δ (ppm) 186.9 (186.8) (**C**HO), 151.7 (NH**C**OO), 150.3 (**C-2**), 148.0 (147.8) (**C-4**), 138.2 (137.8) (**C**H=CH_2), 115.3 (114.3) (CH=**C**H$_2$), 105.1 (**C**Cl$_3$), 93.2 (**C-5**), 91.3 (**C**(CH$_3$)$_2$), 86.2 (**C-4'**), 84.6 (**C-1'**), 72.5 (72.1) (**C-3'**), 63.4 (**C-5'**), 52.3 (51.1) (**C-6**), 39.1 (38.7) (**C-2'**), 26.14 (26.10) (Si-C(**C**H$_3$)$_3$), 21.7 (21.6) (C(**C**H$_3$)$_2$), 18.6

(Si-**C**(CH_3)$_3$), 18.2 (18.1) (Si-**C**(CH_3)$_3$), -4.5 (Si-**C**H_3), -4.6 (Si-**C**H_3), -5.1 (Si-**C**H_3), -5.2 (Si-**C**H_3).

HR-MS: (ESI positive, $CHCl_3$/MeOH), $[M+Na]^+$ calcd for $C_{29}H_{50}Cl_3N_3O_7Si_2Na$: 736.21451, found: 736.21428.

8e:

R_f: 0.34 (Hex/EtOAc = 3:1 *v/v*).

IR: (CCl_4) ν_{max} = 2929, 2857, 1816, 1654, 1463, 1389, 1257, 1138, 837, 789 cm^{-1}.

^{1}H-NMR: (400 MHz, $CDCl_3$) δ (ppm) 9.61 (s, 1H, C**H**O), 9.59 (s, 1H, C**HO***), 6.08-5.96 (m, 2H, **H-1’** , **H-1’***), 5.87-5.74 (m, 2H, C**H**═CH$_2$, C**H***═CH$_2$), 5.26-5.15 (m, 2H, CH═C**H$_2$**, CH═C**H$_2$***), 5.13-5.02 (m, 4H, CH═C**H$_2$**, C**H**-CH═CH$_2$, CH═C**H$_2$***, C**H***-CH═CH$_2$), 4.42-4.23 (m, 2H, **H-3’**, **H-3’***), 3.86-3.74 (m, 2H, **H-4’**, **H-4’***), 3.74-3.60 (m, 2H, **H-5’**, **H-5’***), 3.54-3.50 (m, 2H, **H-5’**, **H-5’***), 2.15-2.08 (m, 2H, **H-2’**, **H-2’***), 2.03-1.85 (m, 26H, **H-2’**, **H-2’***, 2xC(C**H$_3$**)$_2$, 2xC(C**H$_3$**)$_2$*****), 0.91-0.89 (m, 36H, 2xSi-C(C**H$_3$**)$_3$, 2xSi-C(C**H$_3$**)$_3$*****), 0.11-0.06 (m, 24H, 2xSi-(C**H$_3$**)$_2$, 2xSi-(C**H$_3$**)$_2$*****).

^{13}C-NMR: (100 MHz, $CDCl_3$) δ (ppm) 183.9 (**C**HO), 153.5 (C-2), 149.7 (N**C**OO) 142.8 (C-4), 135.6 (**C**H═CH$_2$), 116.4 (CH═**C**H$_2$), 111.9 (**C-5**), 104.9 (CCl_3), 92.5 (**C**(CH_3)$_2$), 86.9 (**C-4’**), 85.4 (**C-1’**), 72.5 (72.3)(**C-3’**), 63.5 (**C-5’**), 50.9 (C-6), 38.0 (**C-2’**), 26.2 (26.1) (Si-C(**C**H$_3$)$_3$), 25.97 (25.90) (Si-C(**C**H$_3$)$_3$), 21.5 (21.4) (C(**C**H$_3$)$_2$), 18.6 (18.2), -4.5 (-4.6), -5.2.

HR-MS: (ESI positive, $CHCl_3$/MeOH), $[M+Na]^+$ calcd for $C_{34}H_{55}Cl_6N_3O_9Si_2Na$: 938,15002, found: 938,15004.

3’,5’-Di(*tert*-butyldimethylsilyl)-3,6-dihydro-5-formyl-6-methyl-2’-deoxycytidine (9a)

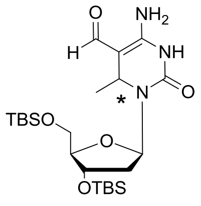

9a

10% Cd–Pb couple (80.0 mg, 711 µmol cadmium, 5.0 equiv) was added in one portion to a vigorously stirred mixture of diastereomers **8a** (100 mg, 142 µmol, 1.0 equiv) in THF (2.20 mL) and aqueous NH_4OAc (1.0 M, 2.20 mL). After stirring for three and a half hours another portion of 10% Cd–Pb (80.0 mg, 712 µmol, 5.0 equiv) was added. The reaction mixture was stirred at room temperature overnight, and then the solids were filtered and rinsed with H_2O and DCM. The phases were separated and the aqueous layer was extracted with DCM (2 × 5 mL). The combined organic layers were washed with brine (5 mL), dried over $MgSO_4$ and the solvents were removed under reduced pressure. The crude product was purified by column chromatography (DCM/MeOH, 60:1 → 15:1 *v/v*) to yield a mixture of diastereomers **9a** with a ratio of 2.4:1 (28.5 mg, 56.0 µmol, 40%) as a pale yellow waxy solid.

R_f: 0.32 (DCM/MeOH = 15:1 *v/v*).

IR: (CCl_4) ν_{max} = 2929, 2857, 1698, 1655, 1541, 1382, 1338, 1255, 1081, 835, 777, 667 cm^{-1}.

^{1}H-NMR: (300 MHz, $CDCl_3$) δ (ppm) 9.64 (s, 1H, N**H**), 9.55 (s, 1H, N**H***), 8.91 (s, 2H, C**H**O, C**H**O*), 6.20-6.13 (m, 1H, **H-1'**), 6.13-6.07 (m, 1H, **H-1'***), 4.55 (q, *J* = 6.4 Hz, 1H, **H-6***), 4.40-4.33 (m, 3H, **H-6, H-3'**, **H-3'***), 3.83-3.79 (m, 2H, **H-4'**, **H-4'***), 3.73-3.65 (m, 4H, **H-5'**, **H-5'***), 2.22-2.02 (m, 2H, **H-2'**, **H-2'***), 1.97-1.84 (m, 2H, **H-2'**, **H-2'***), 1.34 (d, *J* = 6.4 Hz, 3H, CH-C**H**$_3$), 1.29-1.23 (m, 3H, CH-C**H**$_3$*), 0.91 (s, 18H, Si-C(C**H$_3$**)$_3$, Si-C(C**H$_3$**)$_3$*), 0.89-0.88 (m, 18H, Si-C(C**H$_3$**)$_3$, Si-C(C**H$_3$**)$_3$*), 0.08 (s, 12H, Si-(C**H$_3$**)$_2$, Si-(C**H$_3$**)$_2$*), 0.07 (s, 12H, Si-(C**H$_3$**)$_2$, Si-(C**H$_3$**)$_2$*).

^{13}C-NMR: (100 MHz, $CDCl_3$) δ (ppm) 182.9 (182.7) (**C**HO), 153.6 (153.5), 153.3, 92.8 (**C-5**), 86.8 (86.3) (**C-4'**), 84.9 (84.7) (**C-1'**), 72.8 (72.3) (**C-3'**), 63.6 (63.3) (**C-5'**), 47.2 (46.5) (**C-6**), 38.5 (38.1) (**C-2'**), 26.1 (Si-C(**C**H$_3$)$_3$), 25.93 (25.90) (Si-C(**C**H$_3$)$_3$), 25.7 (25.3) (CH-**C**H$_3$,), 18.5 (Si-**C**(CH$_3$)$_3$), 18.2 (18.1) (Si-**C**(CH$_3$)$_3$), -4.5 (Si-**C**H$_3$), -4.59 (-4.62) (Si-**C**H$_3$), -5.2 (Si-**C**H$_3$), -5.3 (-5.4) (Si-**C**H$_3$).

HR-MS: (ESI positive, $CHCl_3$/MeOH), $[M+H]^+$ calcd for $C_{23}H_{46}N_3O_5Si_2$: 500.29705, found: 500.29709, $[M+Na]^+$ calcd for $C_{23}H_{45}N_3O_5Si_2Na$: 522.27900, found: 522.27887.

3',5'-Di(*tert*-butyldimethylsilyl)-3,6-dihydro-5-formyl-6-(trimethylsilylethynyl)-2'-deoxycytidine (9b)

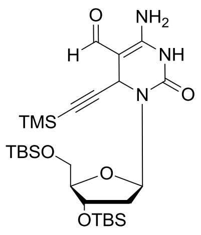

9b

10% Cd–Pb couple (44.0 mg, 391 µmol cadmium, 2.9 equiv) was added in one portion to a vigorously stirred solution of diastereomer **8b** (106 mg, 134 µmol, 1.0 equiv) in THF (2.00 mL) and aqueous NH_4OAc (1.0 M, 2.00 mL). After stirring for three and a half hours the solids were filtered and rinsed with H_2O and DCM. The phases were separated and the aqueous layer was extracted with DCM (2 × 10 mL). The combined organic layers were washed with brine (5 mL), dried over $MgSO_4$ and the solvents were removed under reduced pressure. The crude product was purified by column chromatography (DCM/MeOH, 60:1 → 15:1 *v/v*) to yield diastereomer **9b** (60.6 mg, 104 µmol, 77%) as a yellow waxy solid.

R_f: 0.36 (DCM/MeOH = 15:1 *v/v*).

IR: (KBr) ν_{max} = 2929, 2858, 2166, 1706, 1543, 1463, 1362, 1252, 1081, 778, 667, 569 cm^{-1}.

^{1}H-NMR: (400 MHz, $CDCl_3$) δ (ppm) 9.40 (s, 1H, NH), 8.96 (s, 1H, C**H**O), 5.95 (dd, *J* = 8.4, 5.6 Hz, 1H, **H-1'**), 5.04 (s, 1H, **H-6**), 4.39-4.37 (m, 1H, **H-4'**), 3.90-3.87 (m, 1H, **H-3'**), 3.75 (dd, *J* = 10.8, 4.4 Hz, 1H, **H-5'**), 3.64 (dd, *J* = 10.8, 5.7 Hz, 1H, **H-5'**), 2.27-2.21 (m, 1H, **H-2'**), 1.99 (ddd, *J* = 12.9, 5.7, 2.2 Hz, 1H, **H-2'**), 0.91 (s, 9H, Si-C(C**H$_3$**)$_3$), 0.88 (s, 9H, Si-C(C**H$_3$**)$_3$), 0.11 (s, 9H, Si-(**C**H$_3$)$_3$), 0.09-0.08 (m, 6H, Si-(C**H$_3$**)$_2$), 0.07 (s, 6H, Si-(C**H$_3$**)$_2$).

^{13}C-NMR: (100 MHz, $CDCl_3$) δ (ppm) 182.9 (**C**HO), 153.4, 152.8, 104.7 (**C**≡C-TMS), 89.3 (C≡**C**-TMS), 87.4 (**C-4'**), 86.2 (**C-1'**), 73.0 (**C-3'**), 64.0 (**C-5'**), 43.7 (**C-6**), 38.2 (**C-2'**), 26.1 (Si-C(**C**H_3)$_3$), 25.9 (Si-C(**C**H_3)$_3$), 18.6 (Si-**C**(CH_3)$_3$), 18.1 (Si-**C**(CH_3)$_3$), 0.1 (Si-(**C**H_3)$_3$), -4.6 (Si-**C**H_3), -4.6 (Si-**C**H_3), -5.1 (Si-**C**H_3). **C-5** is not observed.

HR-MS: (ESI positive, MeOH), $[M+Na]^+$ calcd for $C_{27}H_{51}N_3O_5Si_3Na$: 604.30287, found: 604.30347.

Optical rotation: $[\alpha]_D^{25}(\deg\ cm^3\ g^{-1}\ dm^{-1}) = +18.8$ (c = 1, $CHCl_3$).

3',5'-Di(*tert*-butyldimethylsilyl)-3,6-dihydro-5-formyl-6-(2-phenylethynyl)-2'-deoxycytidine (9c)

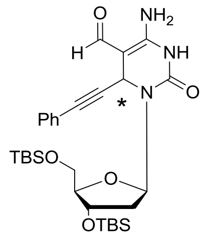

9c

10% Cd–Pb couple (121 mg, 1.08 mmol cadmium, 5.0 equiv) was added in one portion to a vigorously stirred mixture of diastereomers **8c** (170 mg, 215 µmol, 1.0 equiv) in THF (3.00 mL) and aqueous NH_4OAc (1.0 M, 3.00 mL). After stirring for three hours the solids were filtered and rinsed with H_2O and DCM. The phases were separated and the aqueous layer was extracted with DCM (2 × 10 mL). The combined organic layers were washed with brine (5 mL), dried over $MgSO_4$ and the solvents were removed under reduced pressure. The crude product was purified by column chromatography (DCM/MeOH, 60:1 → 15:1 *v/v*) to yield a mixture of diastereomers **9c** with a ratio of 1:1 (55.0 mg, 93.9 µmol, 44%) as a yellow oil.

R_f: 0.32 (DCM/MeOH = 15:1 *v/v*).

IR: (CCl_4) ν_{max} = 3308, 2928, 2857, 1700, 1659, 1541, 1362, 1254, 1097, 834, 779, 690 cm^{-1}.

^{1}H-NMR: (300 MHz, $CDCl_3$) δ (ppm) 9.64-9.59 (m, 2H, N**H**, N**H***), 9.04 (bs, 2H, C**H**O, C**H**O*), 7.37-7.35 (m, 4H, C**H** aromat., C**H*** aromat.), 7.30-7.21 (m, 6H, C**H** aromat., C**H*** aromat.), 6.23 (dd, *J* = 6.9, 6.9 Hz, 1H, **H-1'**), 6.17-6.04 (m, 1H, **H-1'***), 5.54 (s, 1H, **H-6**), 5.30 (s, 1H, **H-6***), 4.45-4.40 (m, 2H), 3.93-3.89 (m, 1H), 3.85-3.77 (m, 3H), 3.75-3.72 (m, 1H), 3.70-3.60 (m, 2H), 2.67-2.58 (m, 1H), 2.31-2.22 (m, 1H), 2.14-1.96 (m, 2H, m, 2H, **H-2'**, **H-2'***), 0.93 (s, 9H, Si-C(C**H$_3$**)$_3$,), 0.88 (s, 9H, Si-C(C**H$_3$**)$_3$*), 0.87-0.86 (m, 18H, Si-C(C**H$_3$**)$_3$), Si-C(C**H$_3$**)$_3$*), 0.12 (s, 6H, Si-(C**H$_3$**)$_2$), 0.08 (s, 6H, Si-(C**H$_3$**)$_2$*), 0.04 (s, 6H, Si-(C**H$_3$**)$_2$), 0.00 (s, 6H, Si-(C**H$_3$**)$_2$*).

^{13}C-NMR: (100 MHz, $CDCl_3$) δ (ppm) 183.0 (182.7) (**C**HO), 153.8 (153.6) , 153.3 (152.5), 131.9 (131.8) (**C** aromat.), 128.5 (**C** aromat.), 128.4 (128.3) (**C** aromat.), 128.3 (**C** aromat.), 122.7 (122.6) (**C** aromat.), 89.4 (89.3), 88.8, 87.3, 86.4, 85.7, 84.2, 83.2, 83.0, 72.9 (72.2) (**C-3'**), 63.9 (63.3) (**C-5'**), 43.1 (41.0) (**C-6'**), 38.4 (38.0) (**C-2'**), 26.2 (26.1) (Si-C(**C**H_3)$_3$),

25.9 (Si-C(**C**H_3)$_3$), 18.6 (18.5) (Si-**C**(CH_3)$_3$), 18.13 (18.10) (Si-**C**(CH_3)$_3$), -4.5 (-4.56) (Si-**C**H_3), -4.57 (-4.67) (Si-**C**H_3), -5.12 (-5.23) (Si-**C**H_3), -5.31 (-5.32) (Si-**C**H_3).

HR-MS: (ESI positive, $CHCl_3$/MeOH), $[M+H]^+$ calcd for $C_{30}H_{48}N_3O_5Si_2$: 586.31270, found: 586.31308, $[M+Na]^+$ calcd for $C_{30}H_{47}N_3O_5Si_2Na$: 608.29465, found: 608.29493.

3',5'-Di(*tert*-butyldimethylsilyl)-3,6-dihydro-5-formyl-6-vinyl-2'-deoxycytidine (9d)

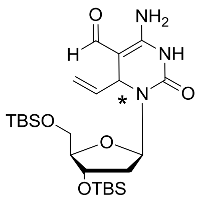

9d

10% Cd–Pb couple (19.7 mg, 175 µmol cadmium, 5.0 equiv) was added in one portion to a vigorously stirred mixture of diastereomers **8d** (25.0 mg, 34.9 µmol, 1.0 equiv) in THF (1.10 mL) and aqueous NH_4OAc (1.0 M, 1.10 mL). The reaction mixture was stirred at room temperature overnight, and then the solids were filtered and rinsed with H_2O and DCM. The phases were separated and the aqueous layer was extracted with DCM (2 × 5 mL). The combined organic layers were washed with brine (5 mL), dried over $MgSO_4$ and the solvents were removed under reduced pressure. The crude product was purified by column chromatography (DCM/MeOH, 30:1 *v/v*) to yield a mixture of the diastereomers **9d** with a ratio of 2.6:1 (11.0 mg, 21.5 µmol, 61%) as a yellow waxy solid.

R_f: 0.47 (DCM/MeOH = 15:1 *v/v*).

IR: (CCl_4) ν_{max} = 3343, 2928, 2857, 1698, 1655, 1541, 1362, 1255, 1096, 836, 778 cm^{-1}.

^{1}H-NMR: (300 MHz, $CDCl_3$) δ (ppm) 9.42 (s, 2H, N**H**, N**H** *), 8.91 (s, 1H, C**HO***), 8.86 (s, 1H, C**H**O), 6.22 (dd, *J* = 8.7, 5.6 Hz, 1H, **H-1'***), 6.12 (dd, *J* = 8.7, 5.6 Hz, 1H, **H-1'**), 5.98 (ddd, *J* = 17.1, 10.1, 4.7 Hz, 1H, CH-C**H**═CH_2), 5.78 (ddd, *J* = 17.0, 10.1, 5.5 Hz, 1H, CH-C**H***═CH_2), 5.11-4.97 (m, 4H, CH-CH═C**H**$_2$, CH-CH═C**H**$_2$*), 4.93 (d, *J* = 5.4 Hz, 1H, **H-6***), 4.76 (d, *J* = 4.7 Hz, 1H, **H-6**), 4.36-4.33 (m, 2H, **H-3'**, **H-3'***), 3.85-3.81 (m, 1H, **H-4'**), 3.78-3.76 (m, 1H, **H-4'***), 3.72- 3.67 (m, 4H, **H-5'**, **H-5'***), 2.23-2.09 (m, 2H, **H-2'**, **H-2'***), 1.97-1.84 (m, 2H, **H-2'**, **H-2'***), 0.91-0.88 (m, 36H, 2xSi-C(C**H$_3$**)$_3$, 2xSi-C(C**H$_3$**)$_3$*), 0.08-0.06 (m, 24H, 2xSi-(C**H$_3$**)$_2$, 2xSi-(C**H$_3$**)$_2$*).

^{13}C-NMR: (75 MHz, $CDCl_3$) δ (ppm) 183.4 (**C**HO), 153.8, 153.2, 139.5 (**C**H═CH_2), 114.7 (CH═**C**H_2), 87.0 (**C-4'**), 85.3 (**C-1'**), 72.4 (**C-3'**), 63.5 (**C-5'**), 52.8 (**C-6**), 38.2 (**C-2'**), 26.14 (26.11) (Si-C(**C**H_3)$_3$), 25.92 (25.90) (Si-C(**C**H_3)$_3$), 18.60 (18.55) (Si-**C**(CH_3)$_3$), 18.15 (18.13) (Si-**C**(CH_3)$_3$), -4.5 (Si-**C**H_3), -4.6 (Si-**C**H_3), -5.2 (Si-**C**H_3), -5.3 (Si-**C**H_3). **C-5** is not observed.

HR-MS: (ESI positive, MeOH), $[M+H]^+$ calcd for $C_{24}H_{46}N_3O_5Si_2$: 512.29705, found: 512.29711, $[M+Na]^+$ calcd for $C_{24}H_{45}N_3O_5Si_2Na$: 534.27900, found: 534.27848.

NMR Spectra:

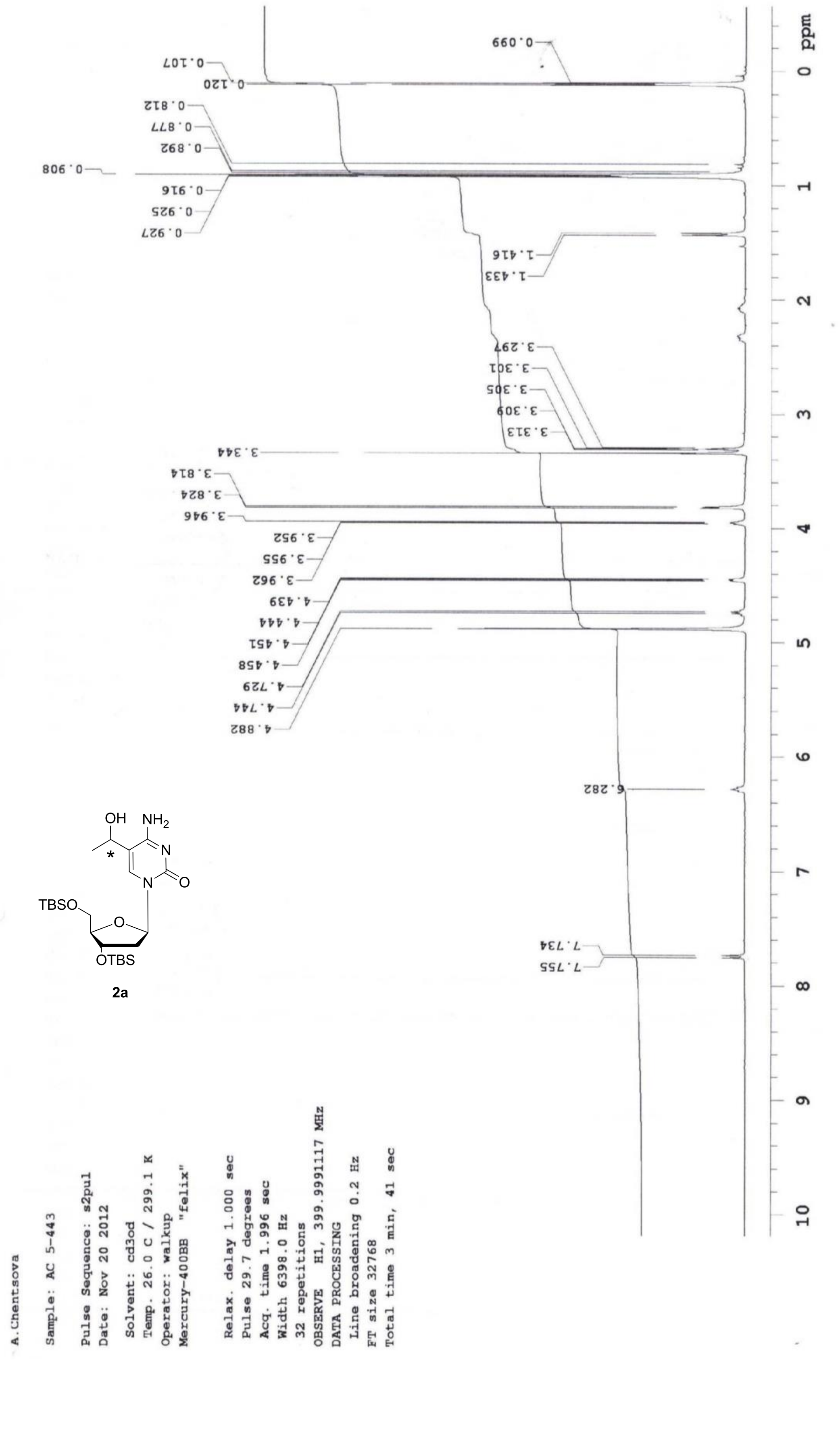
A.Chentsova
Sample: AC 5-443
Pulse Sequence: s2pul
Date: Nov 20 2012
Solvent: cd3od
Temp. 26.0 C / 299.1 K
Operator: walkup
Mercury-400BB "felix"
Relax. delay 1.000 sec
Pulse 29.7 degrees
Acq. time 1.996 sec
Width 6398.0 Hz
32 repetitions
OBSERVE H1, 399.9991117 MHz
DATA PROCESSING
Line broadening 0.2 Hz
FT size 32768
Total time 3 min, 41 sec
ppm
OH
NH2
TBSO
OTBS
2a

A.Chentsova

Sample: AC 5-443

Pulse Sequence: APT
Date: Nov 20 2012

Solvent: cd3od
Temp. 26.0 C / 299.1 K
Operator: walkup
Mercury-400BB "felix"

Relax. delay 1.000 sec
1st pulse 90.0 degrees
2nd pulse 135.0 degrees
Acq. time 1.000 sec
Width 24154.6 Hz
1024 repetitions
OBSERVE C13, 100.5797244 MHz
DECOUPLE H1, 400.0010689 MHz
Power 38 dB
on during acquisition
WALTZ-16 modulated
DATA PROCESSING
Line broadening 1.0 Hz
FT size 65536
Total time 1 hr, 15 min, 20 sec

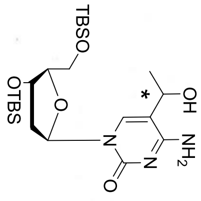

2a

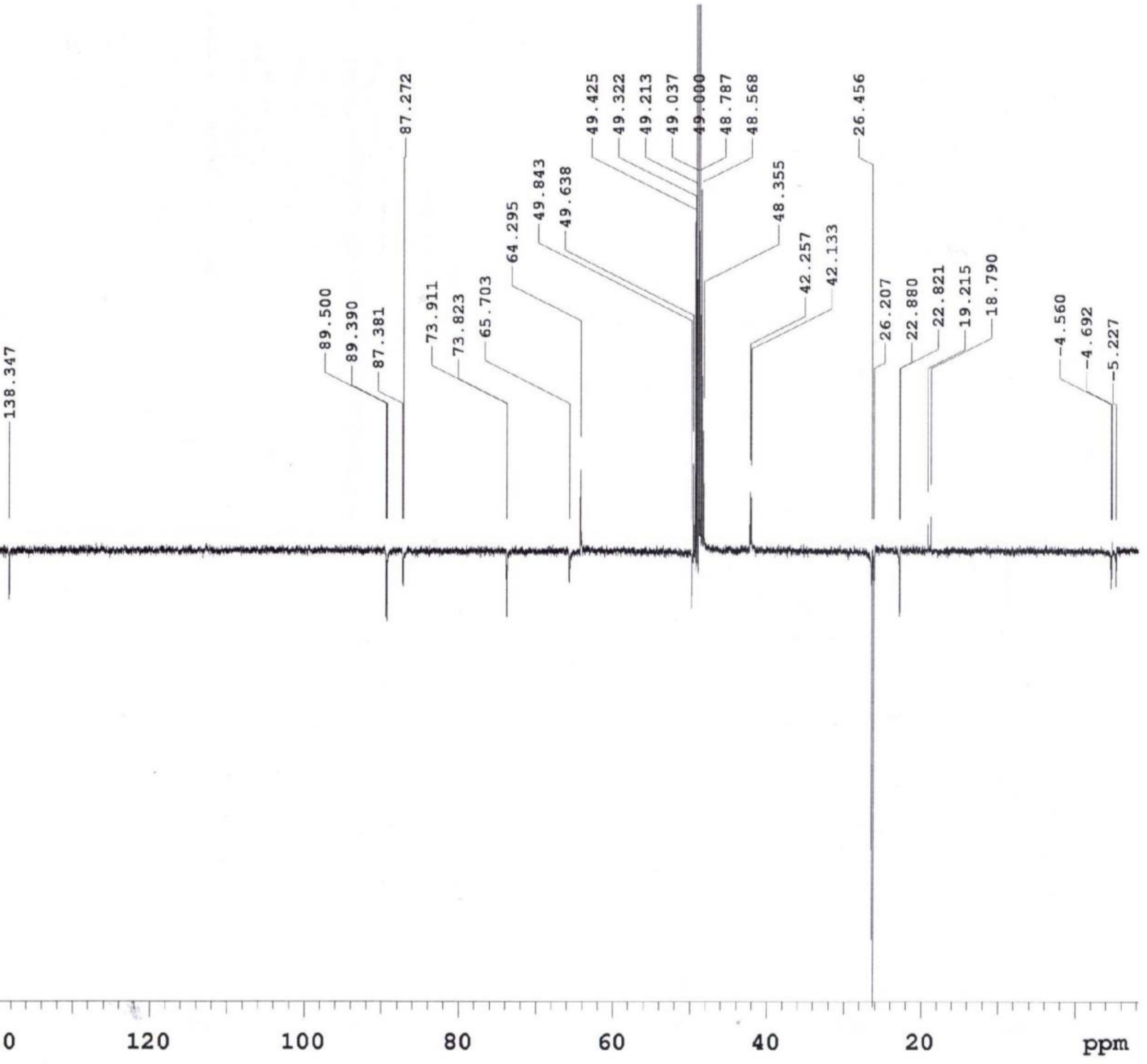

A. Chentsova

Sample: AC5-471

Pulse Sequence: s2pul

Solvent: cd3od
Temp. 26.0 C / 299.1 K
Operator: vnmr1
Mercury-300BB "jutta"

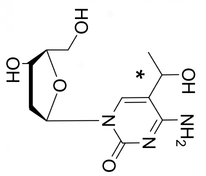

3a

Relax. delay 1.000 sec
Pulse 29.7 degrees
Acq. time 1.998 sec
Width 4800.8 Hz
28 repetitions
OBSERVE H1, 300.0828730 MHz
DATA PROCESSING
Line broadening 0.2 Hz
FT size 32768
Total time 1 min, 47 sec

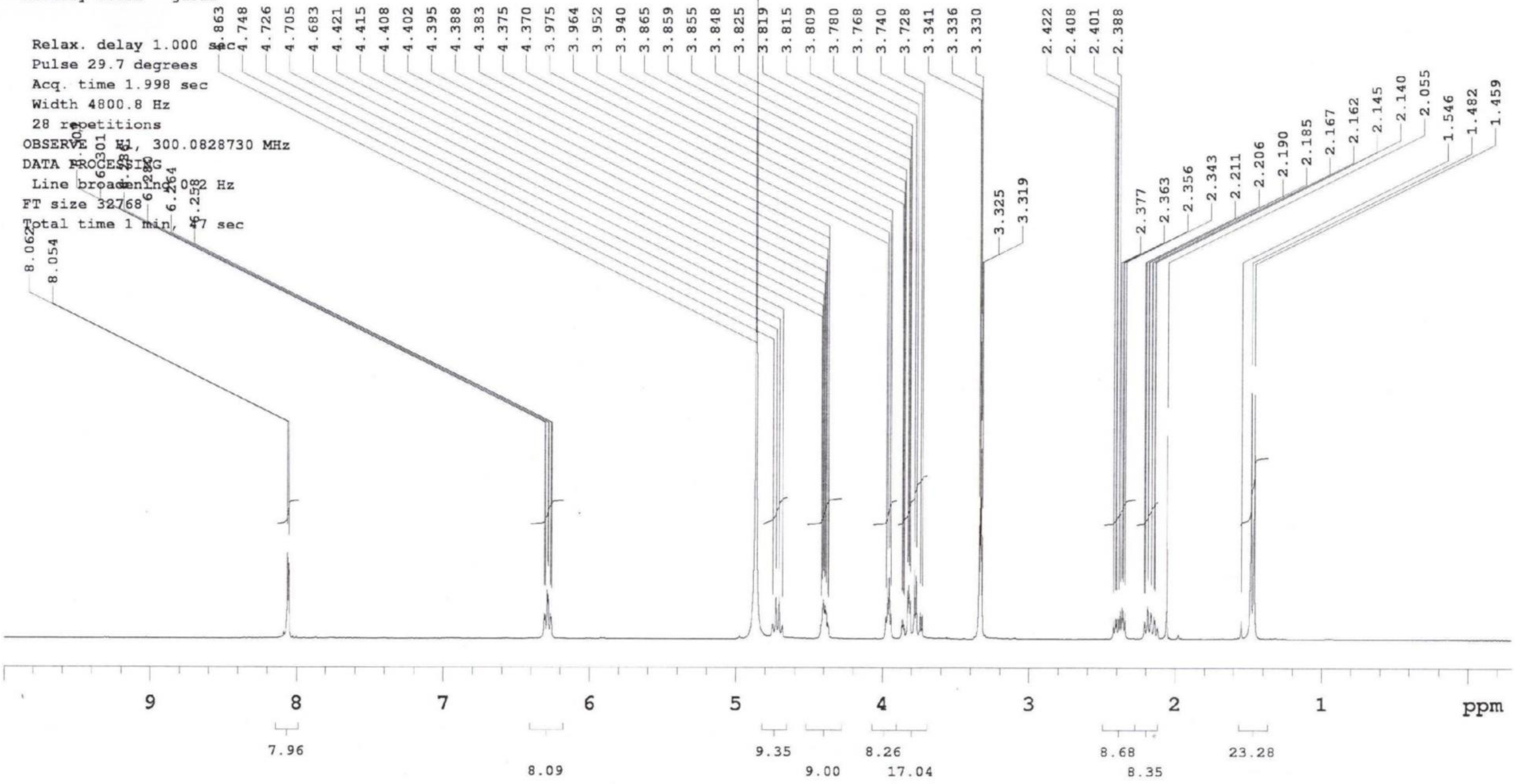

A.Chentsova

Sample: AC 5-471

Pulse Sequence: APT
Date: Dec 3 2012

Solvent: cd3od
Temp. 26.0 C / 299.1 K
Operator: walkup
Mercury-400BB "felix"

Relax. delay 1.000 sec
1st pulse 90.0 degrees
2nd pulse 135.0 degrees
Acq. time 1.000 sec
Width 24154.6 Hz
5000 repetitions
OBSERVE C13, 100.5797134 MHz
DECOUPLE H1, 400.0010689 MHz
Power 38 dB
on during acquisition
WALTZ-16 modulated
DATA PROCESSING
Line broadening 1.0 Hz
FT size 65536
Total time 3 hr, 7 min, 20 sec

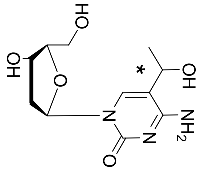

3a

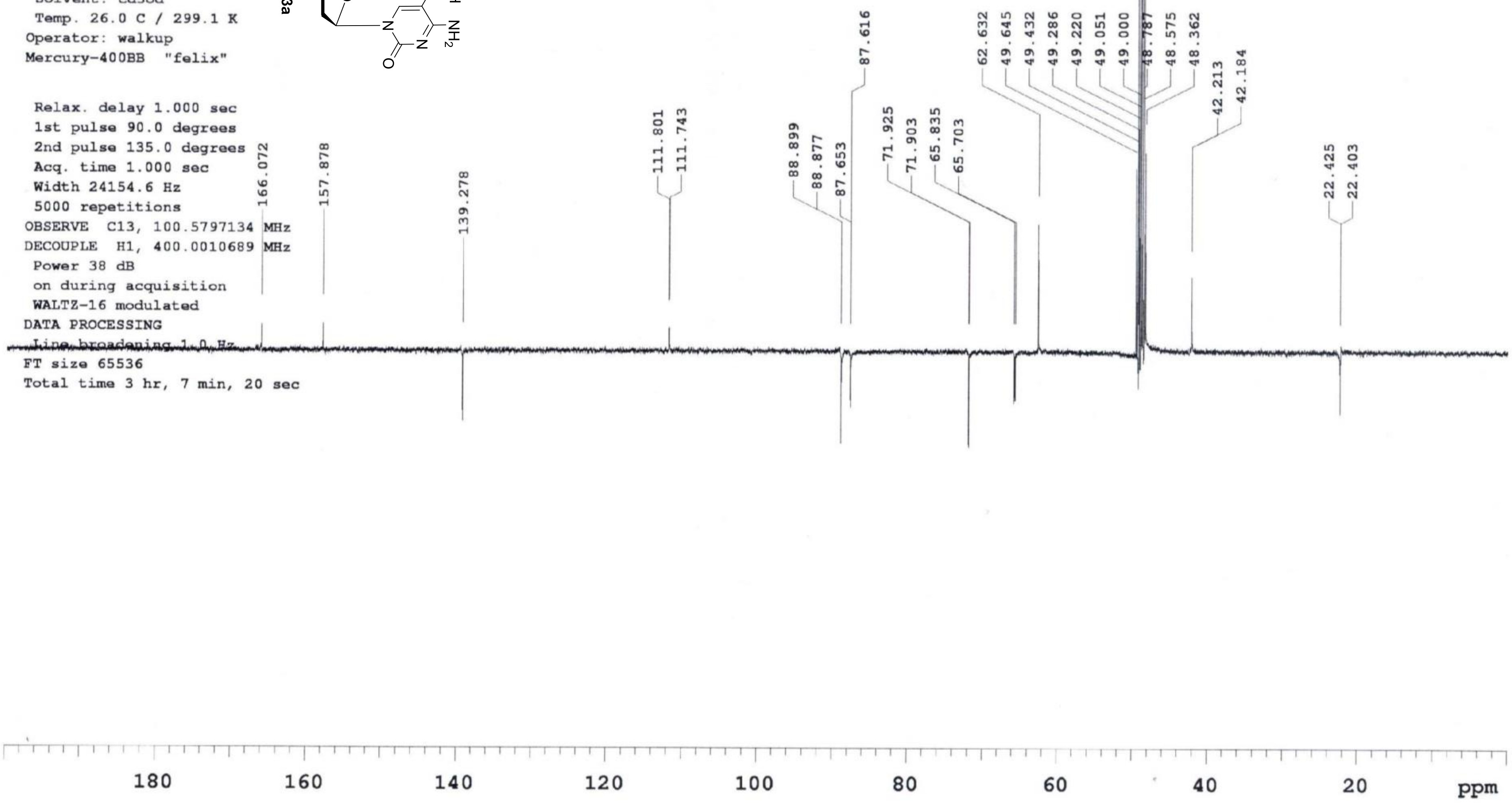

A.Chentsova

Sample: AC5-28AM

Pulse Sequence: s2pul

Solvent: cd3od
Temp. 26.0 C / 299.1 K
Operator: vnmr1
Mercury-300BB "jutta"

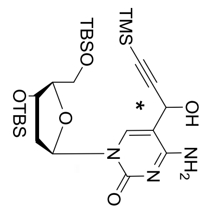

2b

Relax. delay 1.000 sec
Pulse 29.7 degrees
Acq. time 1.998 sec
Width 4800.8 Hz
28 repetitions
OBSERVE H1, 300.0828807 MHz
DATA PROCESSING
Line broadening 0.2 Hz
FT size 32768
Total time 3 min, 35 sec

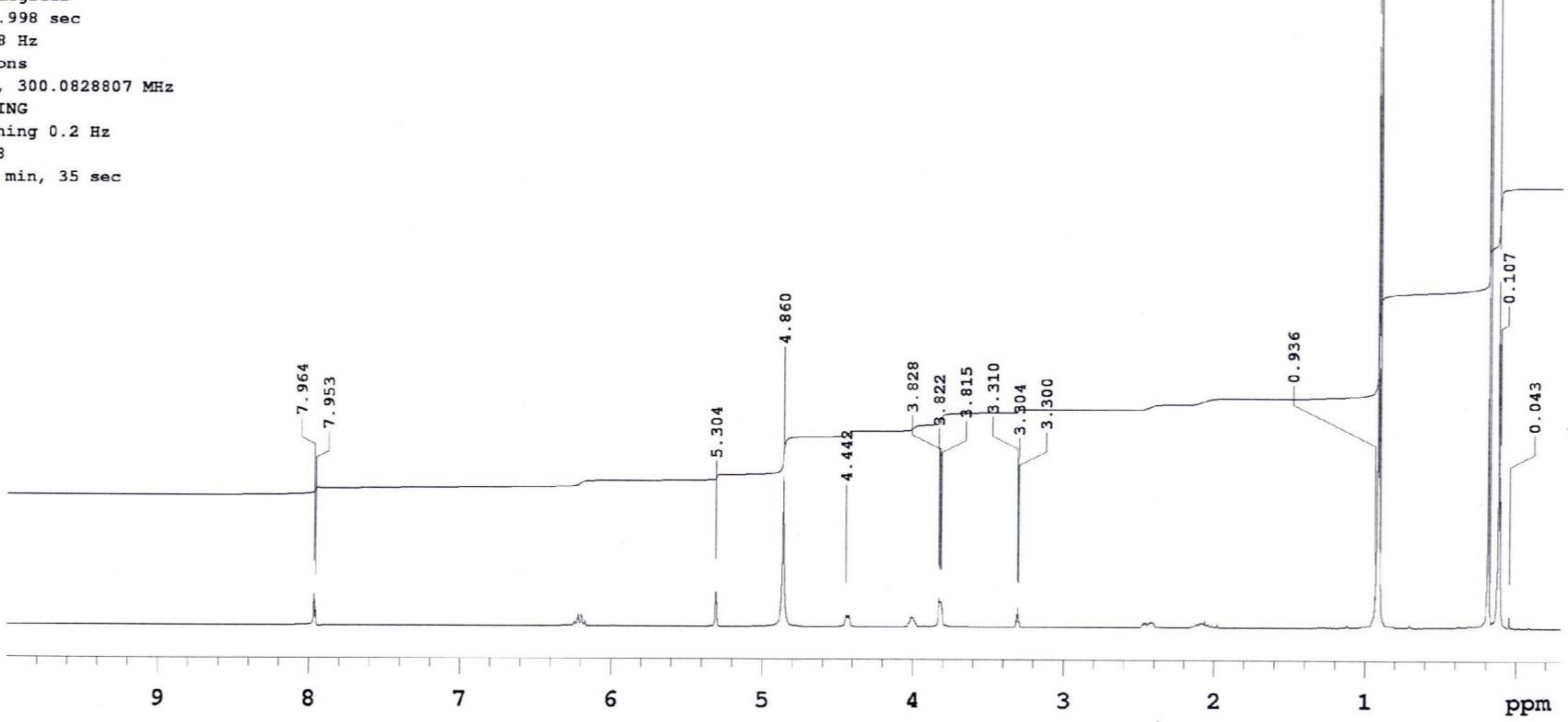

E. Kapourani

Sample: EK-24C

Pulse Sequence: s2pul
Date: Aug 28 2012

Solvent: cdcl3
Temp. 26.0 C / 299.1 K
Operator: walkup
Mercury-400BB "felix"

Relax. delay 1.000 sec
Pulse 45.0 degrees
Acq. time 1.300 sec
Width 24154.6 Hz
2000 repetitions
OBSERVE C13, 100.5794365 MHz
DECOUPLE H1, 399.9994929 MHz
Power 38 dB
continuously on
WALTZ-16 modulated
DATA PROCESSING
Line broadening 1.0 Hz
FT size 65536
Total time 1 hr, 21 min, 50 sec

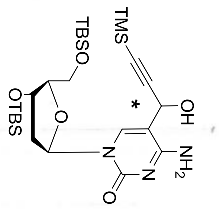

2b

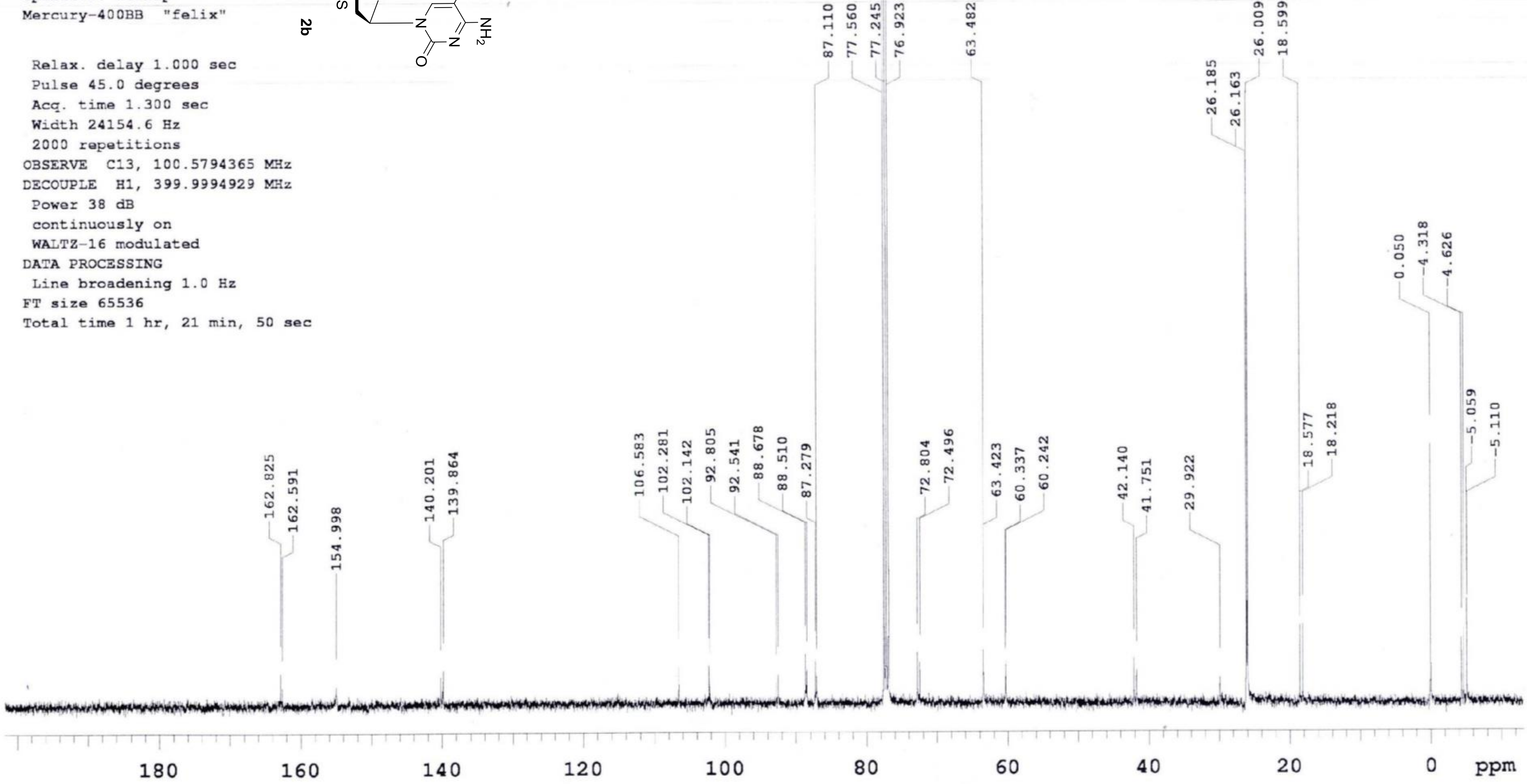

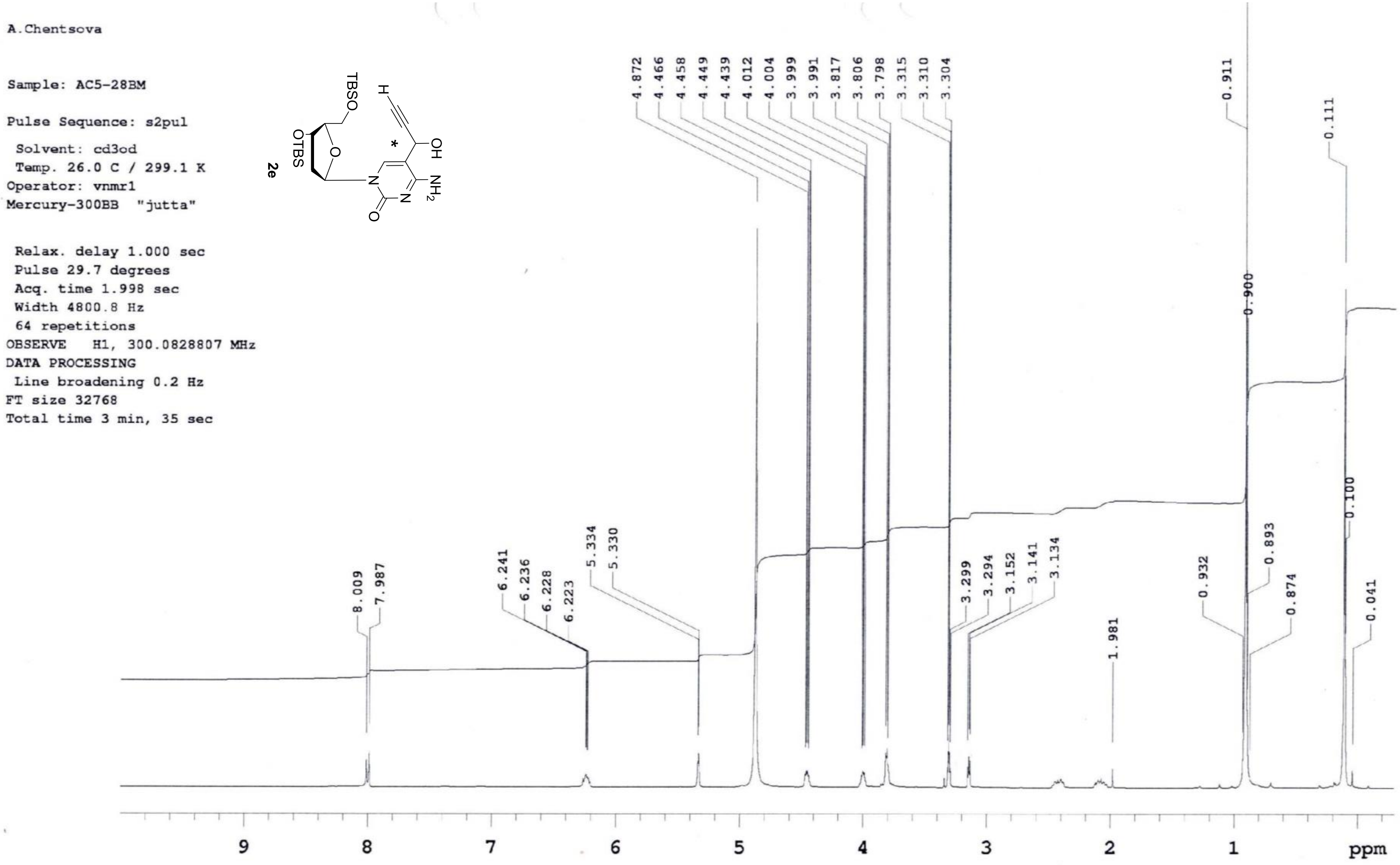
A.Chentsova
Sample: AC5-28BM
Pulse Sequence: s2pul
Solvent: cd3od
Temp. 26.0 C / 299.1 K
Operator: vnmr1
Mercury-300BB "jutta"
Relax. delay 1.000 sec
Pulse 29.7 degrees
Acq. time 1.998 sec
Width 4800.8 Hz
64 repetitions
OBSERVE H1, 300.0828807 MHz
DATA PROCESSING
Line broadening 0.2 Hz
FT size 32768
Total time 3 min, 35 sec
2e
ppm

A. Chentsova

Sample: AC5-28BM

Pulse Sequence: APT
Date: Oct 26 2012

Solvent: cd3od
Temp. 26.0 C / 299.1 K
Operator: walkup
Mercury-400BB "felix"

Relax. delay 1.000 sec
1st pulse 90.0 degrees
2nd pulse 135.0 degrees
Acq. time 1.000 sec
Width 24154.6 Hz
848 repetitions
OBSERVE C13, 100.5797274 MHz
DECOUPLE H1, 400.0010689 MHz
Power 38 dB
on during acquisition
WALTZ-16 modulated
DATA PROCESSING
Line broadening 1.0 Hz
FT size 65536
Total time 1 hr, 15 min, 20 sec

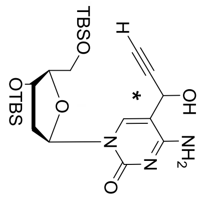

2e

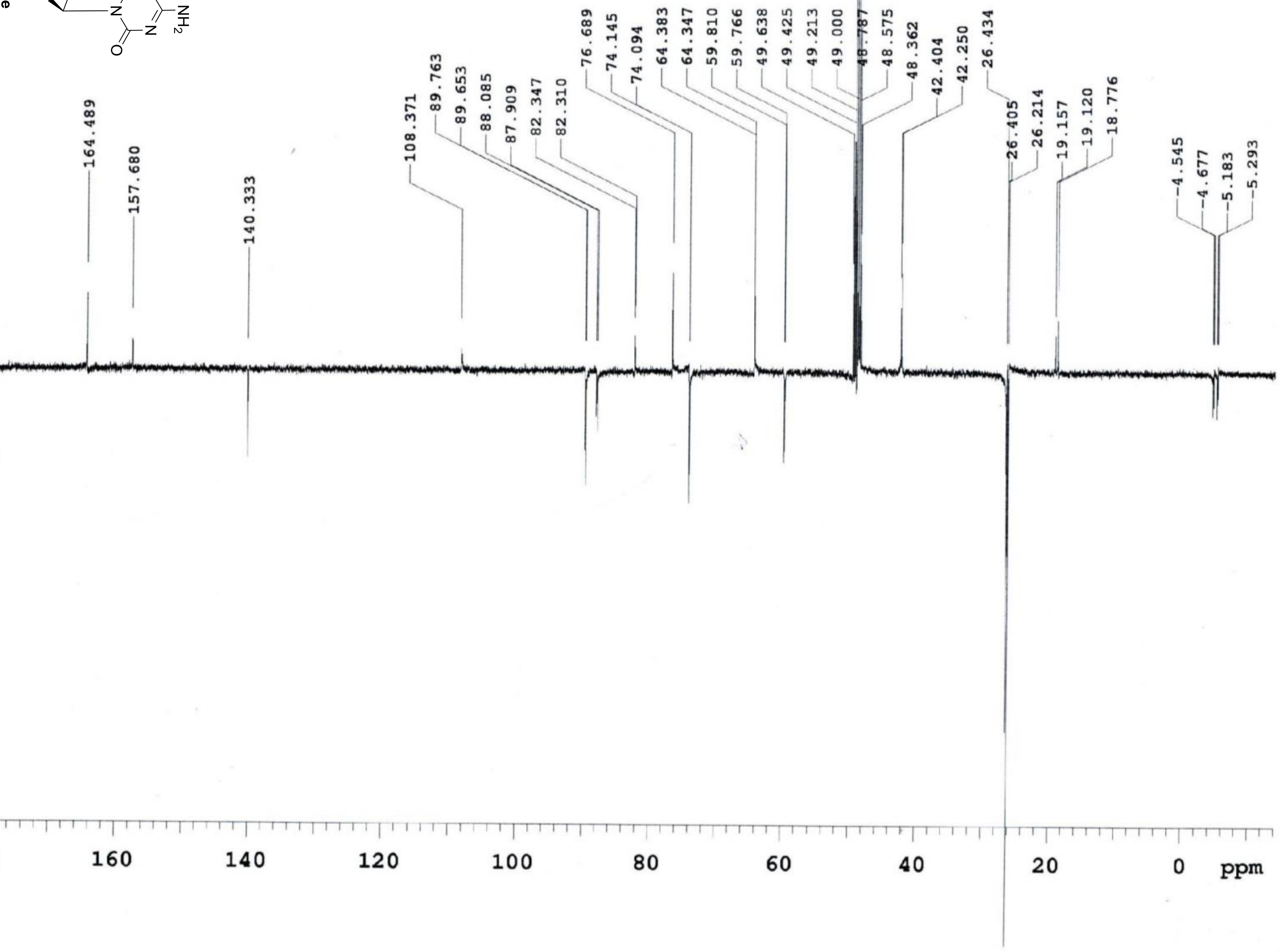

Sample: AC 5-H1

Pulse Sequence: s2pul
Date: Apr 17 2013

Solvent: cd3od
Temp. 26.0 C / 299.1 K
Operator: walkup
Mercury-400BB "felix"

Relax. delay 1.000 sec
Pulse 29.7 degrees
Acq. time 1.996 sec
Width 7199.4 Hz
32 repetitions
OBSERVE H1, 399.9991123 MHz
DATA PROCESSING
Line broadening 0.2 Hz
FT size 32768
Total time 1 min, 52 sec

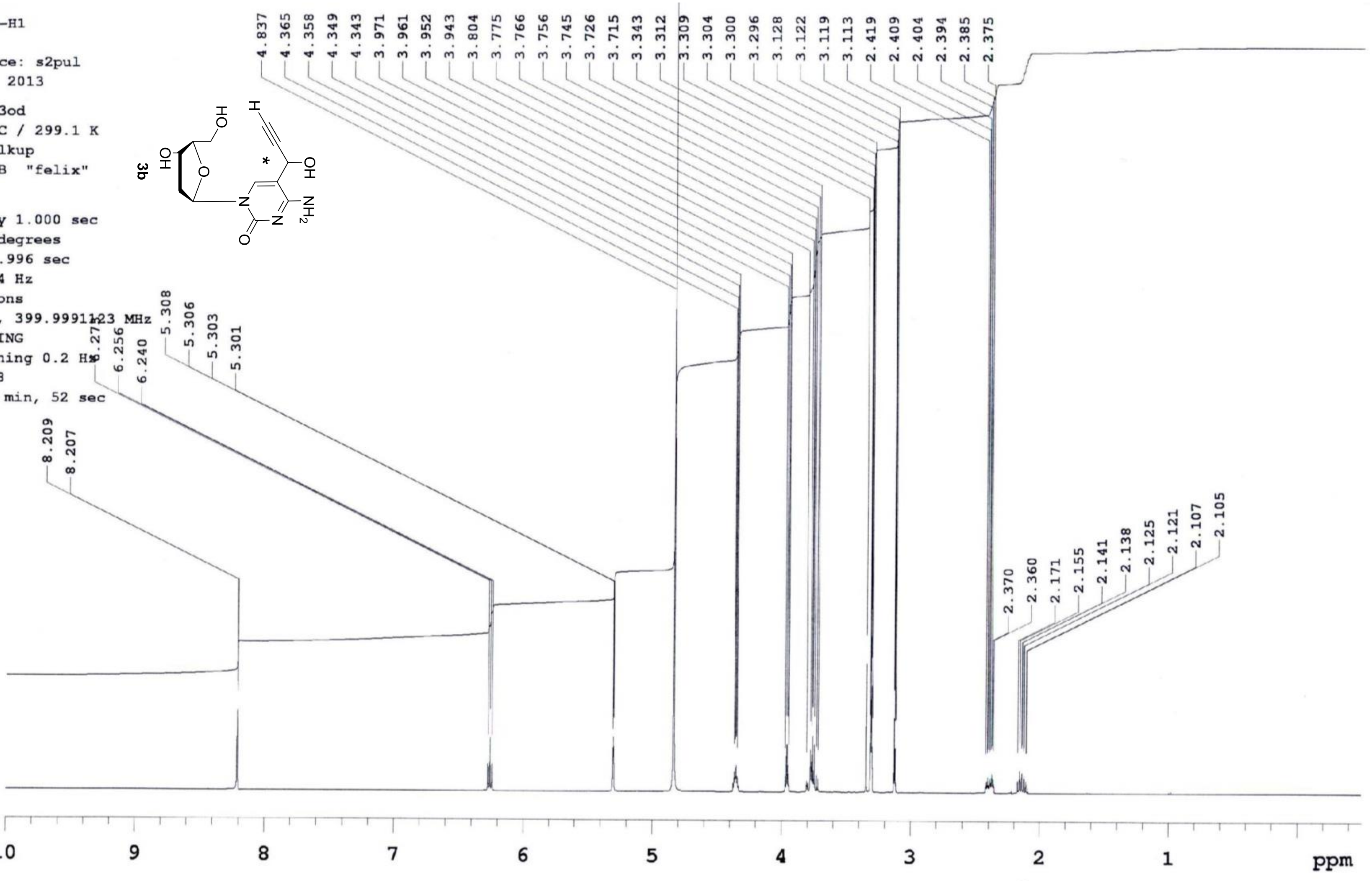

A. Chentsova

Sample: AC5-H1

Pulse Sequence: s2pul

Solvent: cd3od
Temp. 26.0 C / 299.1 K
Operator: vnmr1
Mercury-300BB "jutta"

Relax. delay 1.000 sec
Pulse 45.0 degrees
Acq. time 1.301 sec
Width 18115.9 Hz
736 repetitions
OBSERVE C13, 75.4557935 MHz
DECOUPLE H1, 300.0843291 MHz
Power 37 dB
continuously on
WALTZ-16 modulated
DATA PROCESSING
Line broadening 0.5 Hz
FT size 65536
Total time 1 hr, 24 min, 1 sec

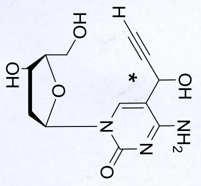

3b

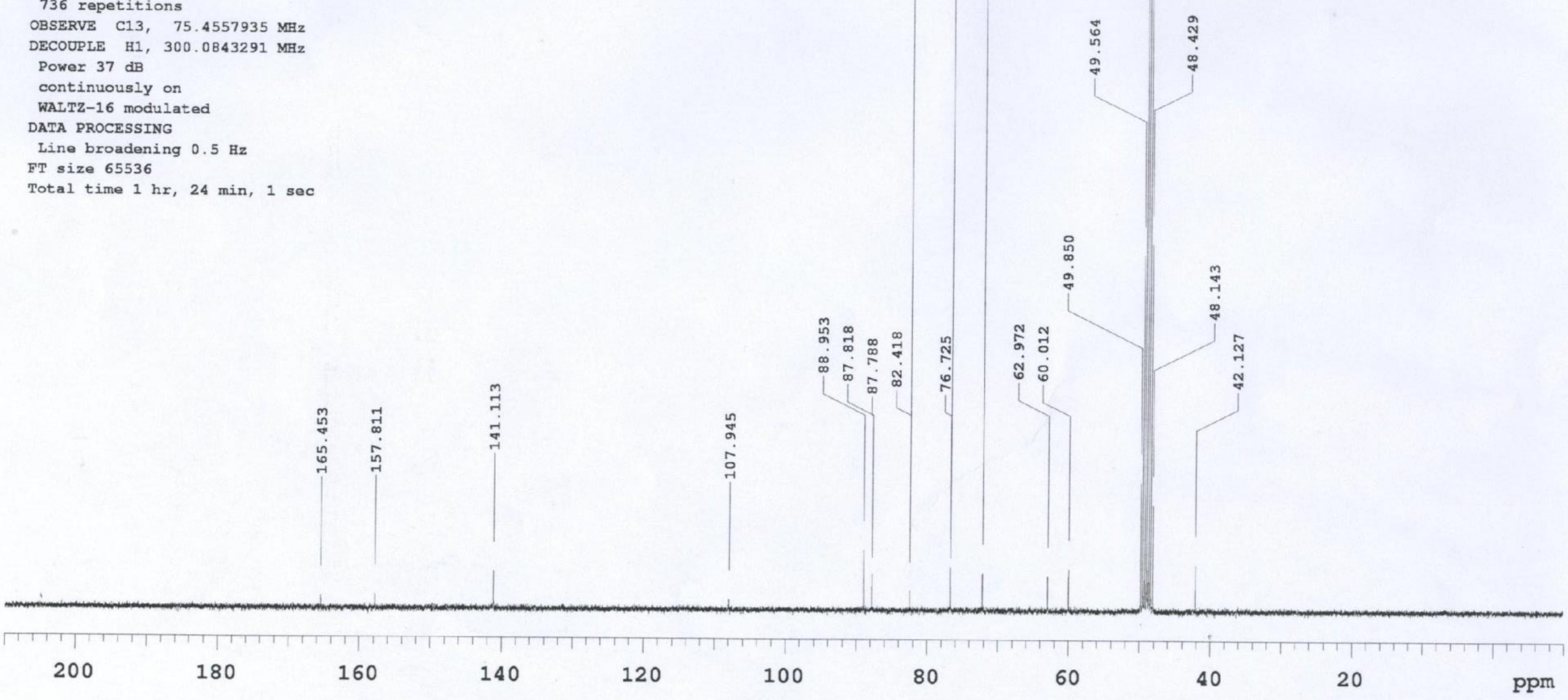

A. Chentsova

Sample: AC5-454

Pulse Sequence: s2pul

Solvent: cd3od
Temp. 26.0 C / 299.1 K
Operator: vnmr1
Mercury-300BB "jutta"

Relax. delay 1.000 sec
Pulse 29.7 degrees
Acq. time 1.998 sec
Width 4800.8 Hz
20 repetitions
OBSERVE H1, 300.0828754 MHz
DATA PROCESSING
Line broadening 0.1 Hz
FT size 32768
Total time 1 min, 47 sec

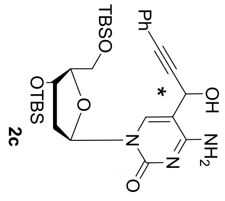

2c

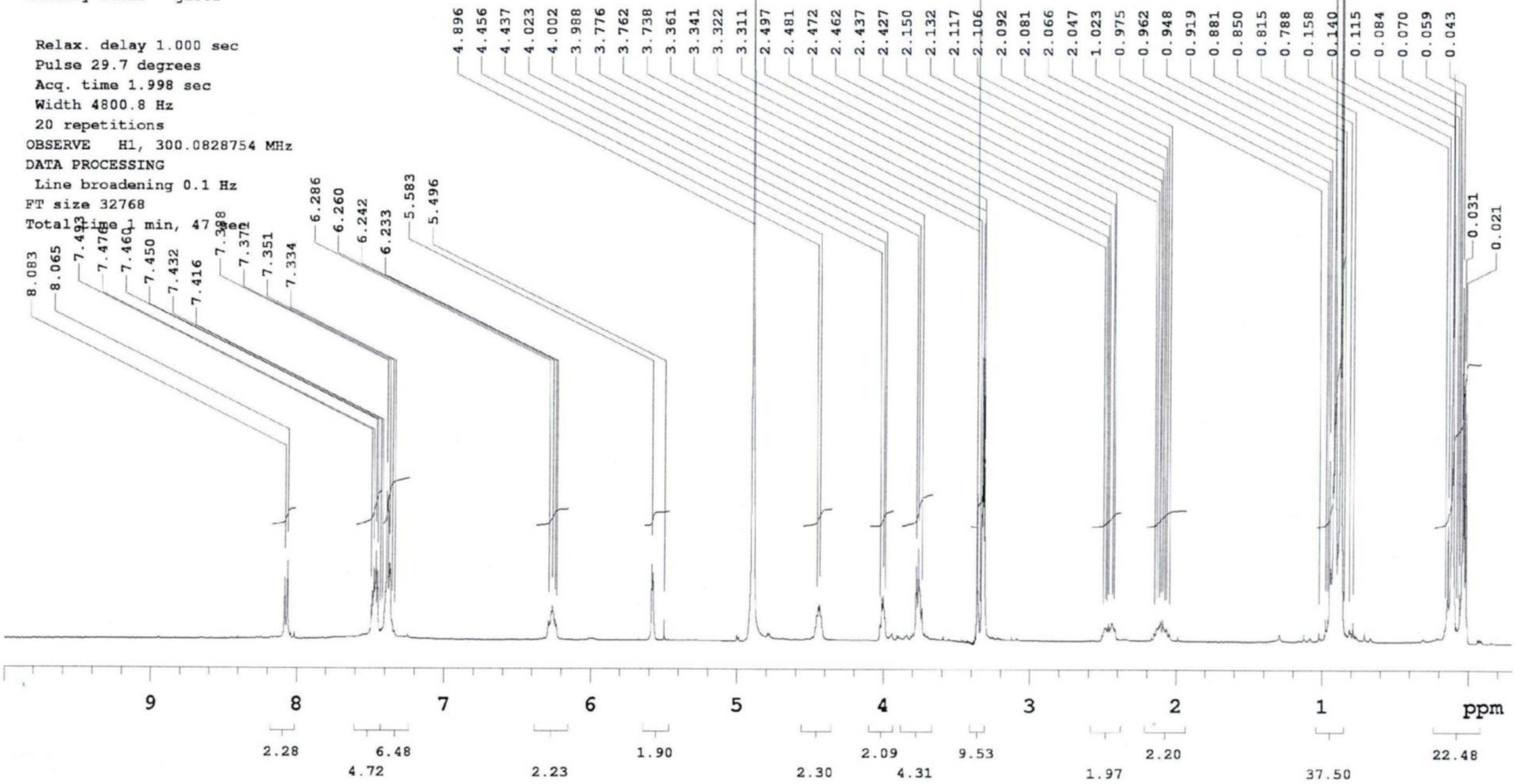

A. Chentsova

Sample: AC-5-454

Pulse Sequence: s2pul

Solvent: cd3od
Temp. 26.0 C / 299.1 K
Operator: vnmr1
Mercury-300BB "jutta"

Relax. delay 1.000 sec
Pulse 45.0 degrees
Acq. time 1.301 sec
Width 18115.9 Hz
1168 repetitions
OBSERVE C13, 75.4557996 MHz
DECOUPLE H1, 300.0843291 MHz
Power 37 dB
continuously on
WALTZ-16 modulated
DATA PROCESSING
Line broadening 0.5 Hz
FT size 65536
Total time 1 hr, 24 min, 1 sec

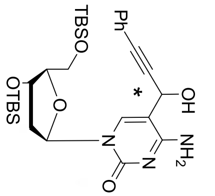

2c

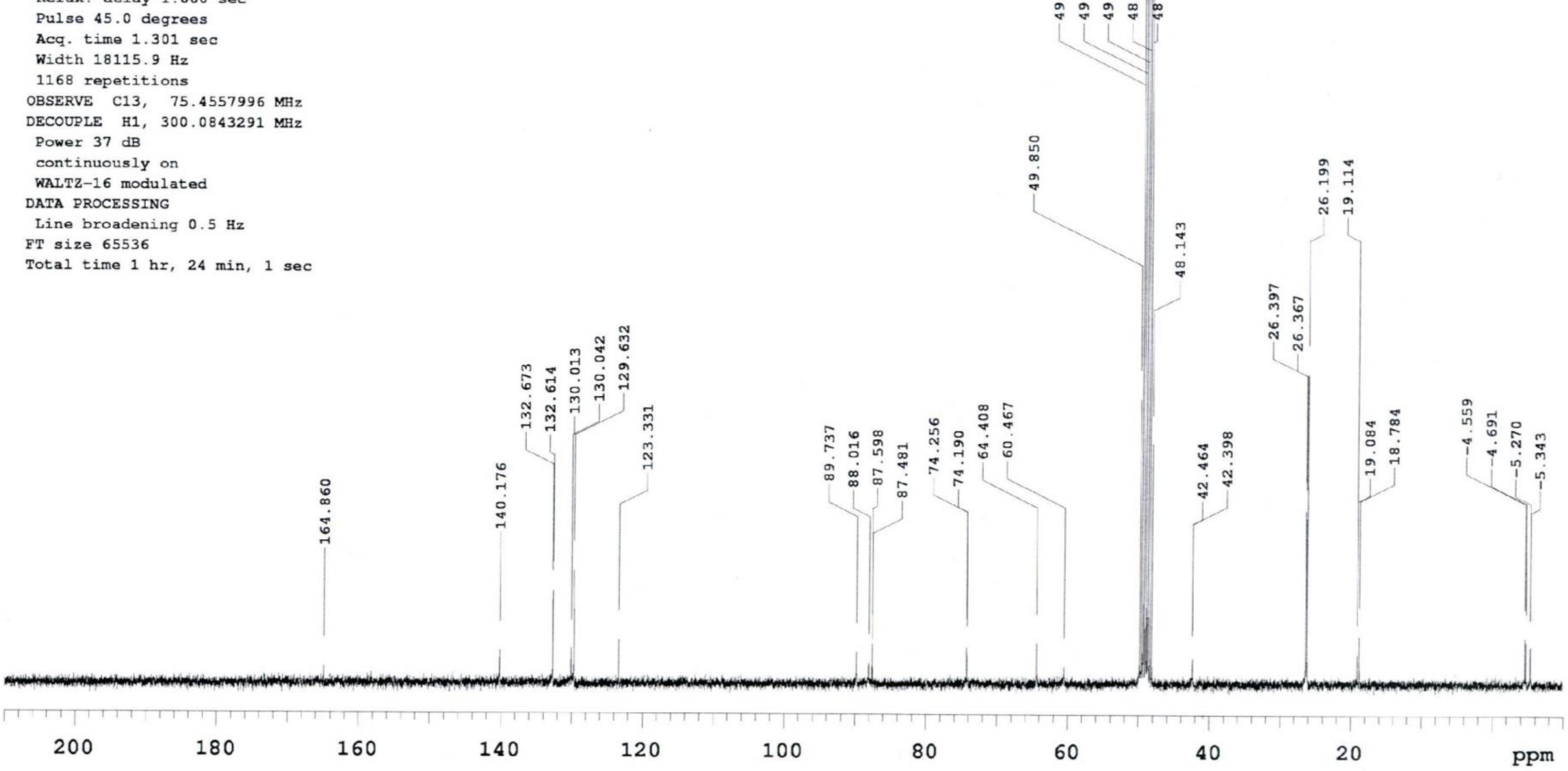

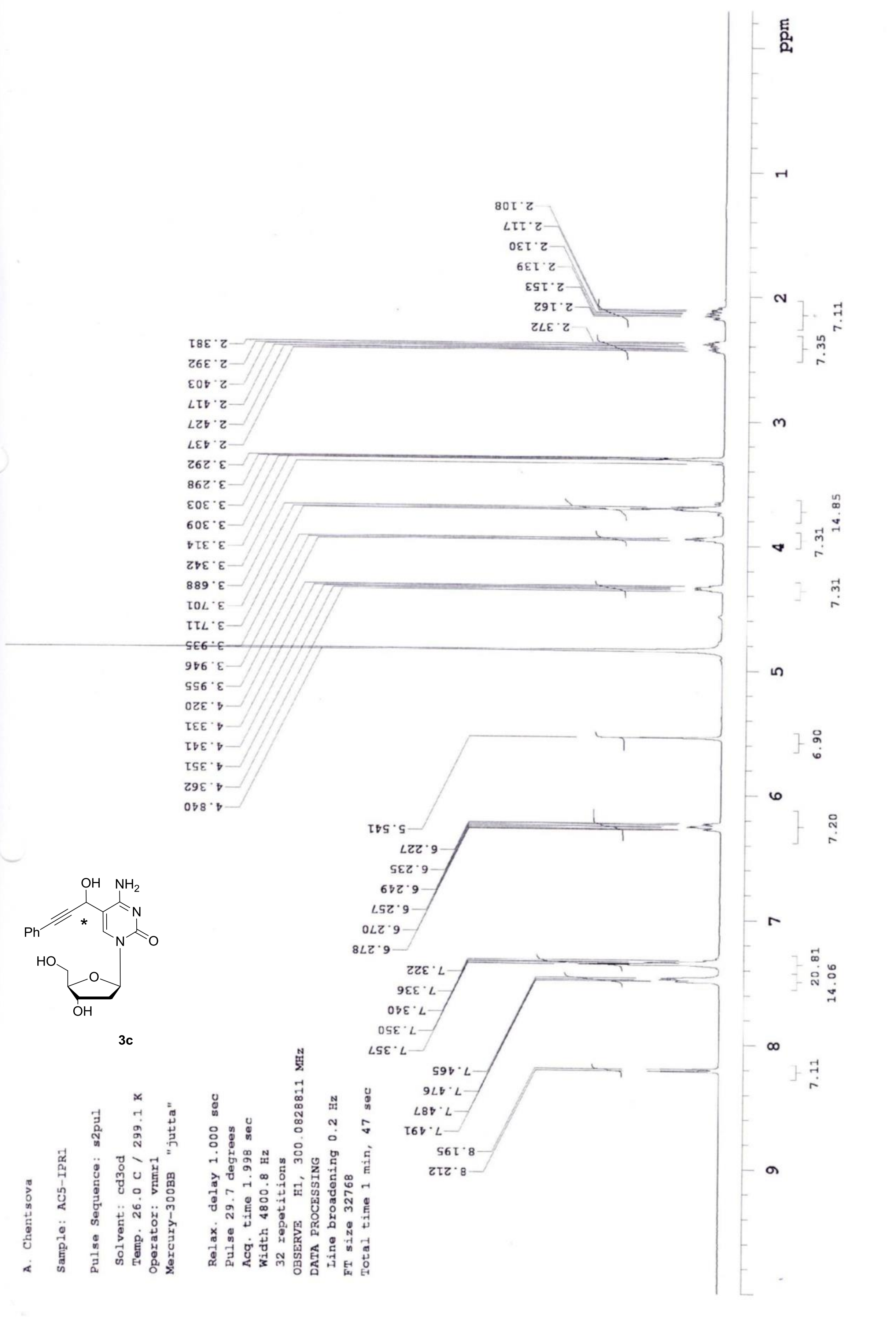

A. Chentsova
Sample: AC5-IPR1
Pulse Sequence: s2pul
Solvent: cd3od
Temp. 26.0 C / 299.1 K
Operator: vnmr1
Mercury-300BB "jutta"
Relax. delay 1.000 sec
Pulse 29.7 degrees
Acq. time 1.998 sec
Width 4800.8 Hz
32 repetitions
OBSERVE H1, 300.0828811 MHz
DATA PROCESSING
Line broadening 0.2 Hz
FT size 32768
Total time 1 min, 47 sec
3c
ppm

Chentsova

Sample: AC 5-IPR1

Pulse Sequence: s2pul
Date: Apr 16 2013

Solvent: cd3od
Temp. 26.0 C / 299.1 K
Operator: walkup
Mercury-400BB "felix"

Relax. delay 1.000 sec
Pulse 45.0 degrees
Acq. time 1.300 sec
Width 24154.6 Hz
512 repetitions
OBSERVE C13, 100.5797156 MHz
DECOUPLE H1, 400.0010689 MHz
Power 38 dB
continuously on
WALTZ-16 modulated
DATA PROCESSING
Line broadening 0.5 Hz
FT size 65536
Total time 1 hr, 25 min, 53 sec

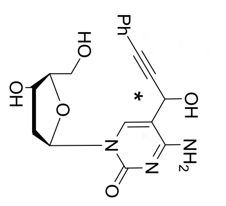

3c

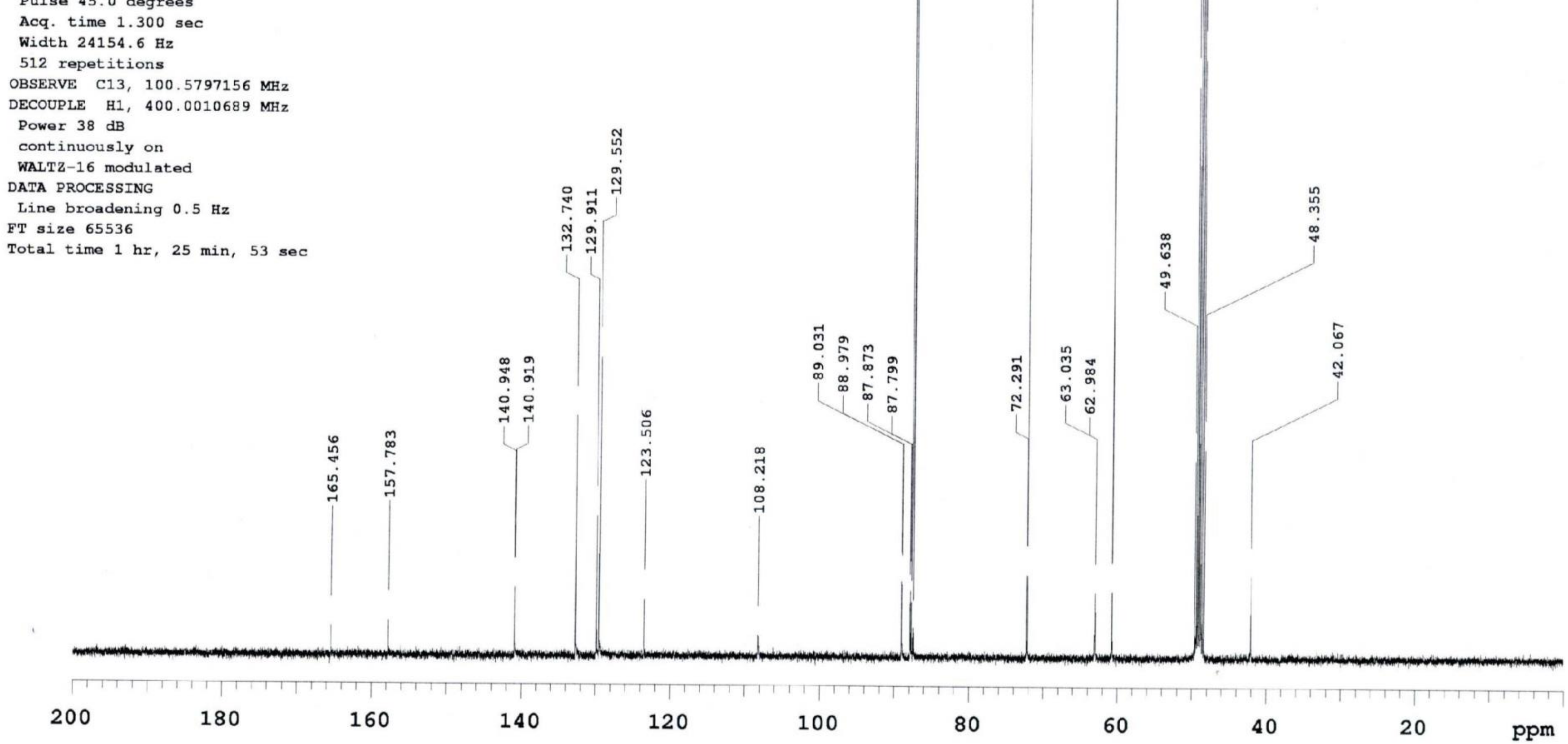

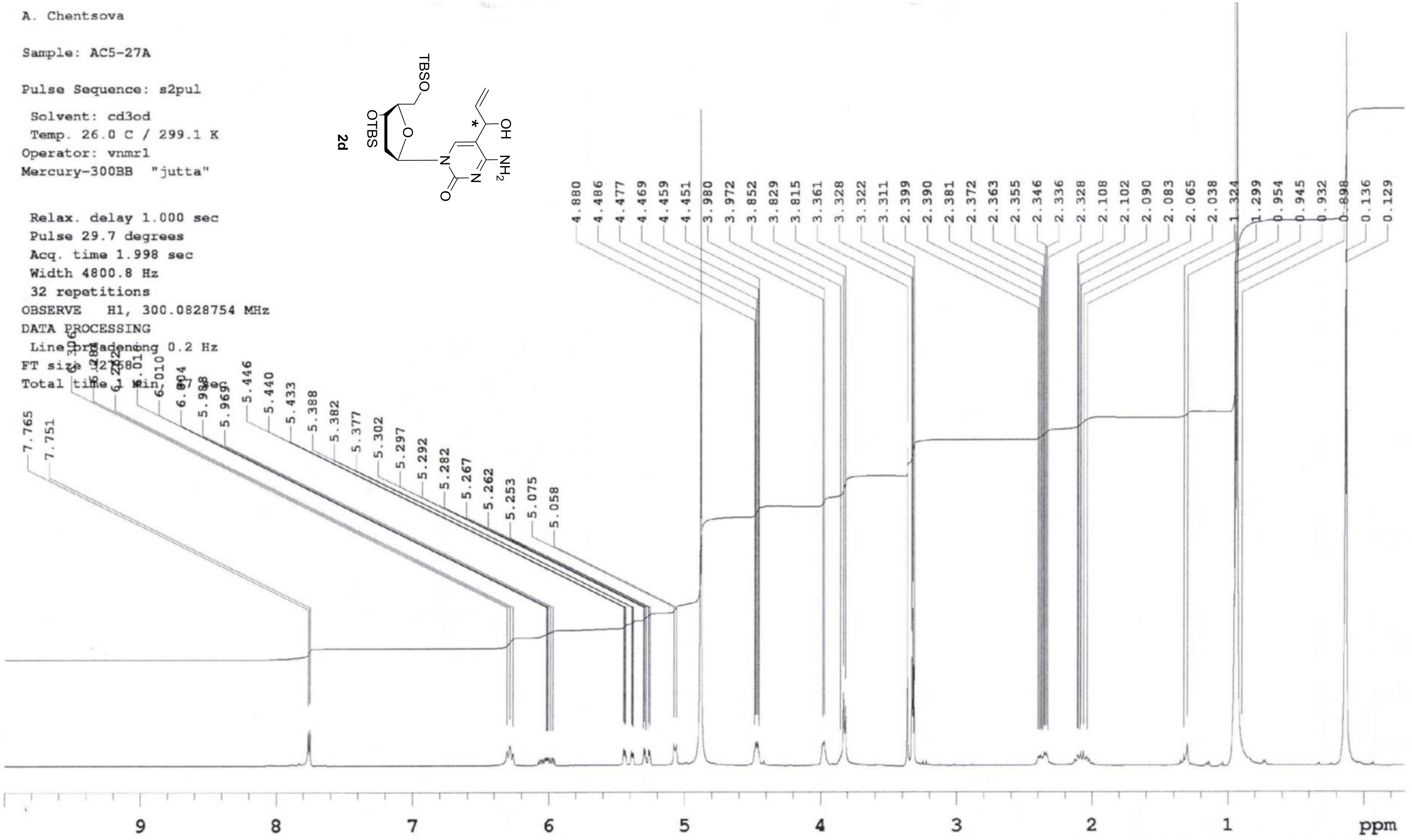
A. Chentsova
Sample: AC5-27A
Pulse Sequence: s2pul
Solvent: cd3od
Temp. 26.0 C / 299.1 K
Operator: vnmr1
Mercury-300BB "jutta"
Relax. delay 1.000 sec
Pulse 29.7 degrees
Acq. time 1.998 sec
Width 4800.8 Hz
32 repetitions
OBSERVE H1, 300.0828754 MHz
DATA PROCESSING
Line broadening 0.2 Hz
FT size 32768
Total time 1 min, 7 sec
TBSO
OTBS
OH
NH2
N
O
2d
7.765
7.751
5.969
5.988
6.004
6.010
5.446
5.440
5.433
5.388
5.382
5.377
5.302
5.297
5.292
5.282
5.267
5.262
5.253
5.075
5.058
4.880
4.486
4.477
4.469
4.459
4.451
3.980
3.972
3.852
3.829
3.815
3.361
3.328
3.322
3.311
2.399
2.390
2.381
2.372
2.363
2.355
2.346
2.336
2.328
2.108
2.102
2.090
2.083
2.065
2.038
1.324
1.299
0.954
0.945
0.932
0.898
0.136
0.129
9
8
7
6
5
4
3
2
1
ppm

Attached proton test experiment

Sample: AC5-27A

Pulse Sequence: APT

Solvent: cd3od
Temp. 26.0 C / 299.1 K
Operator: vnmr1
Mercury-300BB "jutta"

Relax. delay 1.000 sec
1st pulse 90.0 degrees
2nd pulse 135.0 degrees
Acq. time 1.000 sec
Width 18115.9 Hz
50000 repetitions
OBSERVE C13, 75.4557969 MHz
DECOUPLE H1, 300.0843291 MHz
Power 37 dB
on during acquisition
WALTZ-16 modulated
DATA PROCESSING
Line broadening 1.0 Hz
FT size 65536
Total time 28 hr, 56 min, 53 sec

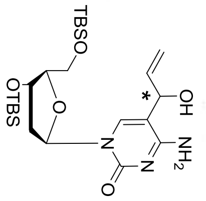

2d

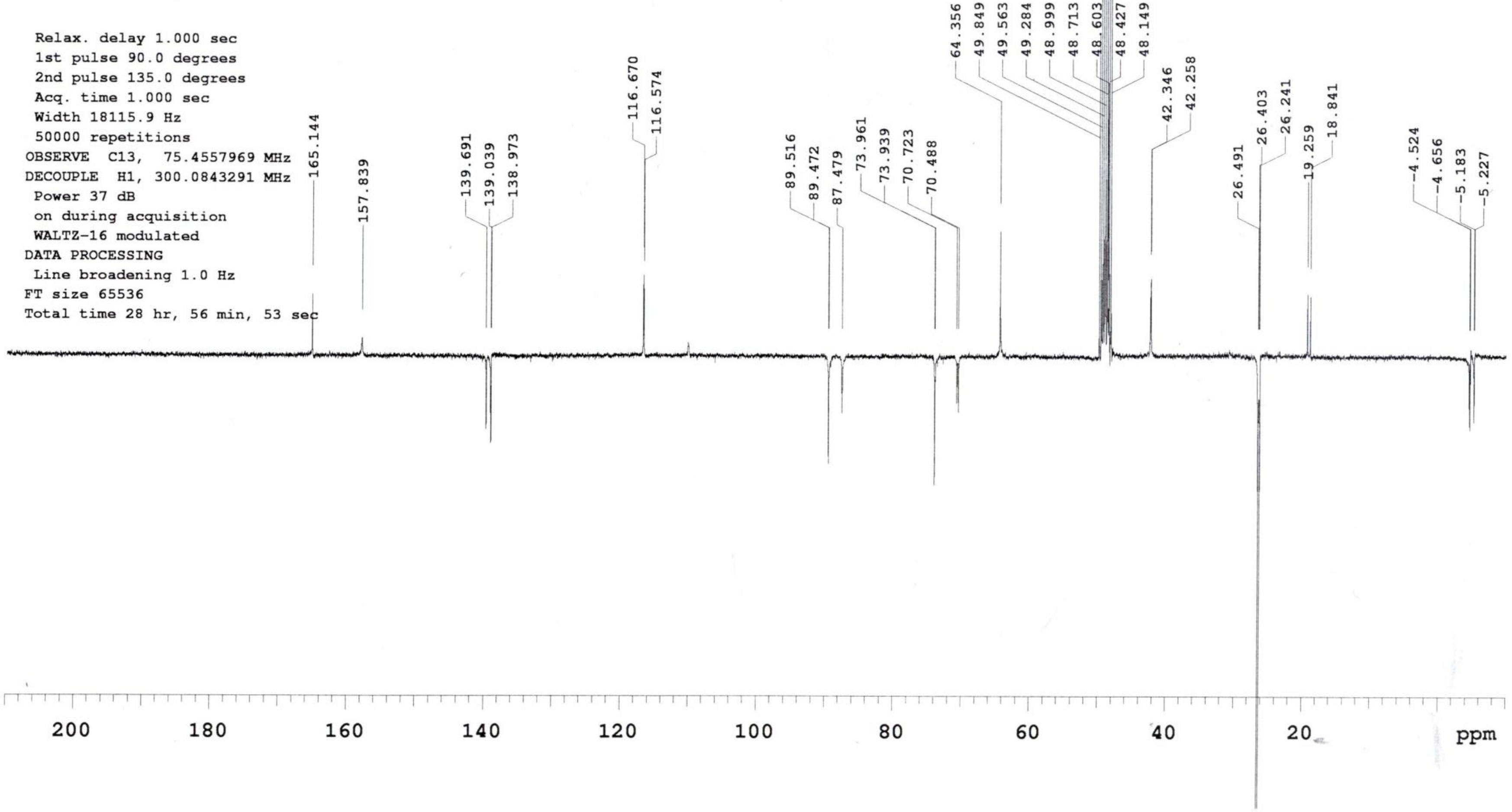

A. Chentsova

Sample: AC5-JN

Pulse Sequence: s2pul
Date: Apr 8 2013

Solvent: cd3od
Temp. 26.0 C / 299.1 K
Operator: walkup
Mercury-400BB "felix"

Relax. delay 1.000 sec
Pulse 29.7 degrees
Acq. time 1.996 sec
Width 6398.0 Hz
32 repetitions
OBSERVE H1, 399.9901123 MHz
DATA PROCESSING
Line broadening 0.1 Hz
FT size 32768
Total time 1 min, 50 sec

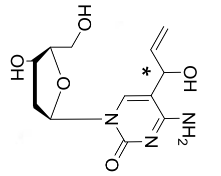

3d

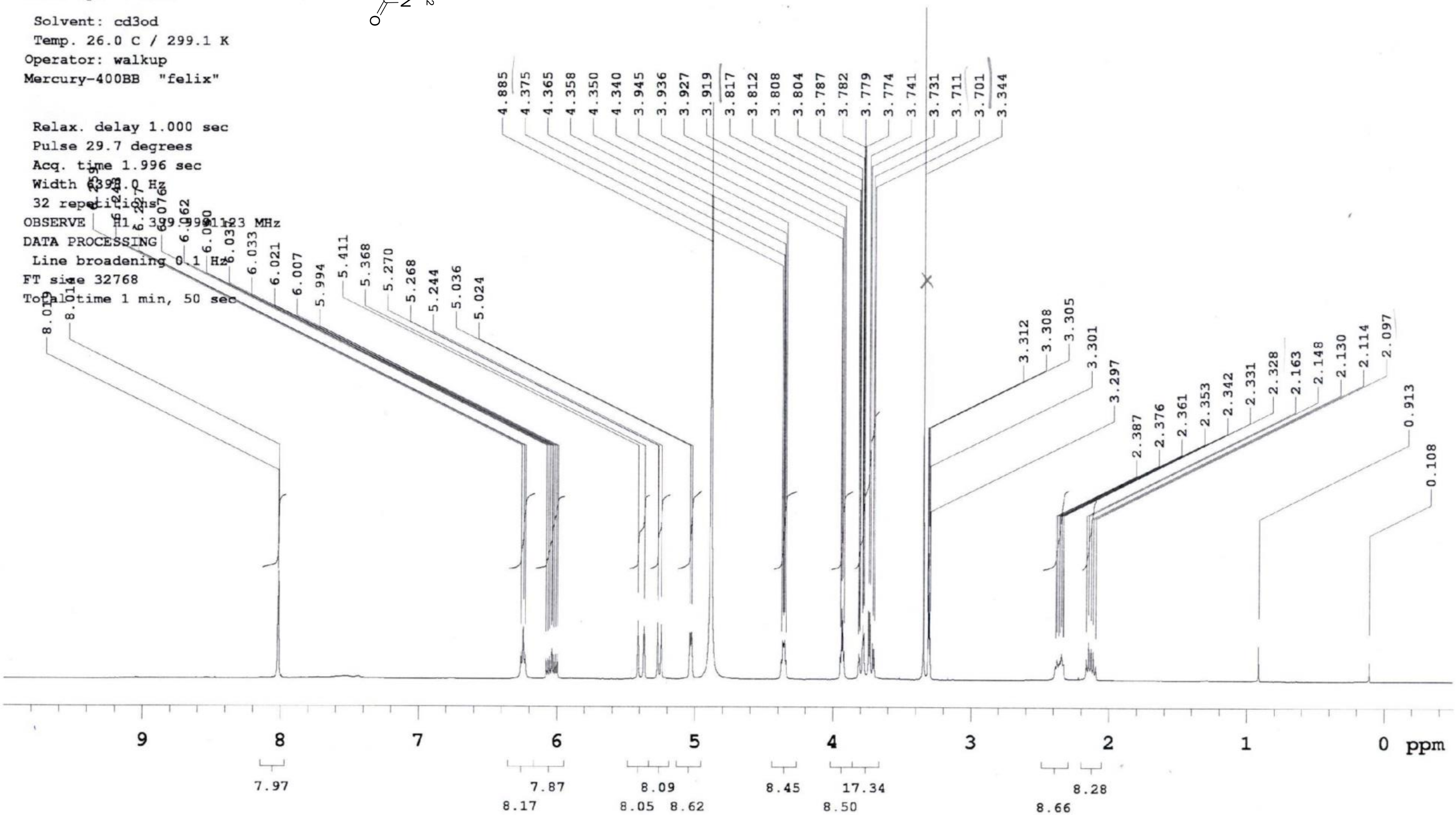

Chentsova

Sample: AC 5-IN

Pulse Sequence: s2pul

Solvent: cd3od
Temp. 26.0 C / 299.1 K
Operator: vnmr1
Mercury-300BB "jutta"

Relax. delay 1.000 sec
Pulse 45.0 degrees
Acq. time 1.301 sec
Width 18115.9 Hz
528 repetitions
OBSERVE C13, 75.4557963 MHz
DECOUPLE H1, 300.0843291 MHz
Power 37 dB
continuously on
WALTZ-16 modulated
DATA PROCESSING
Line broadening 0.5 Hz
FT size 65536
Total time 42 min, 2 sec

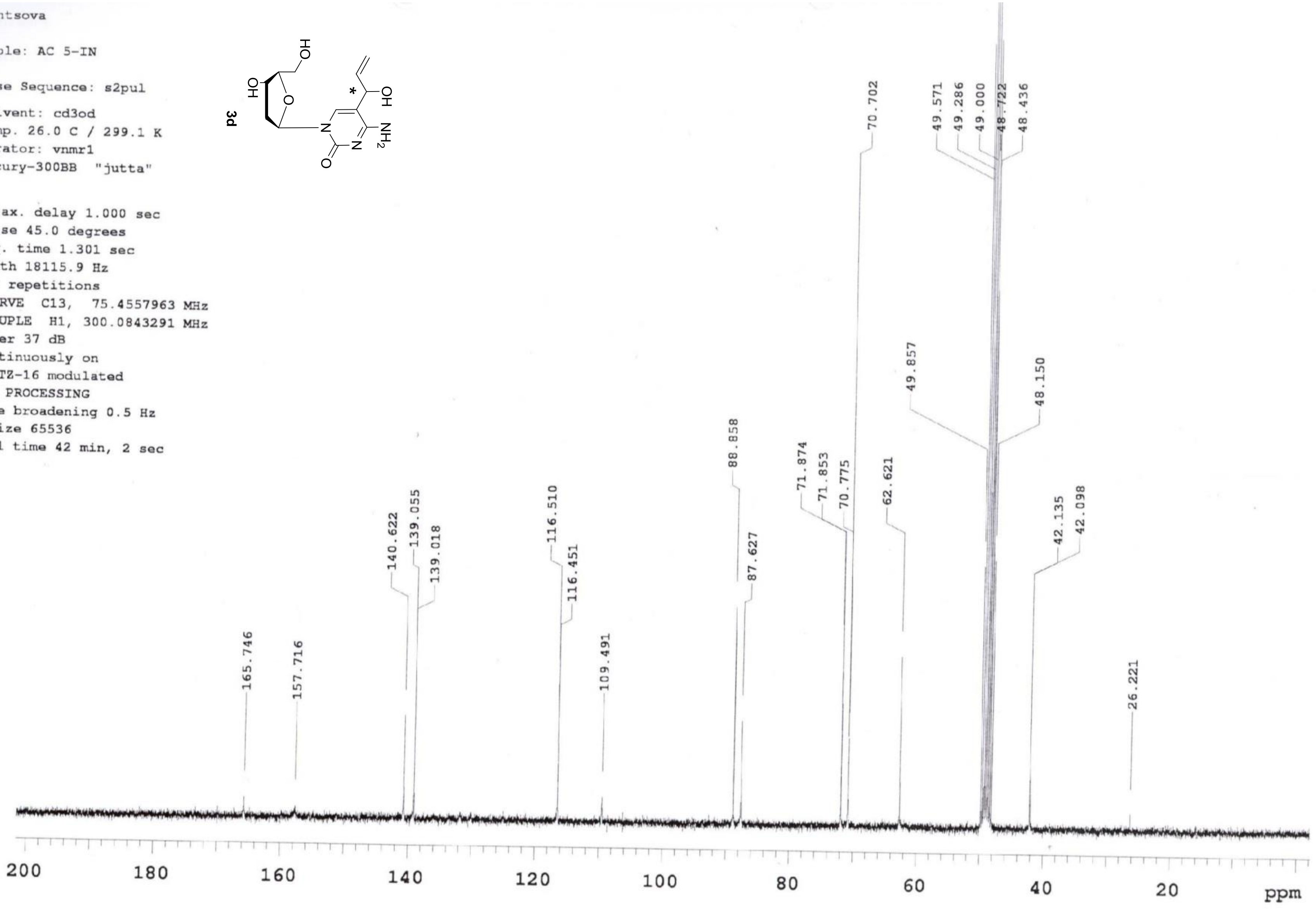

A. Chentsova

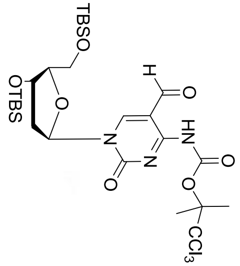

4

Sample: AC5-13B

Pulse Sequence: s2pul
Date: Aug 8 2012

Solvent: cdcl3
Temp. 26.0 C / 299.1 K
Operator: walkup
Mercury-400BB "felix"

Relax. delay 1.000 sec
Pulse 45.0 degrees
Acq. time 1.996 sec
Width 6398.0 Hz
32 repetitions
OBSERVE H1, 399.9975361 MHz
DATA PROCESSING
Line broadening 0.2 Hz
FT size 32768
Total time 1 min, 50 sec

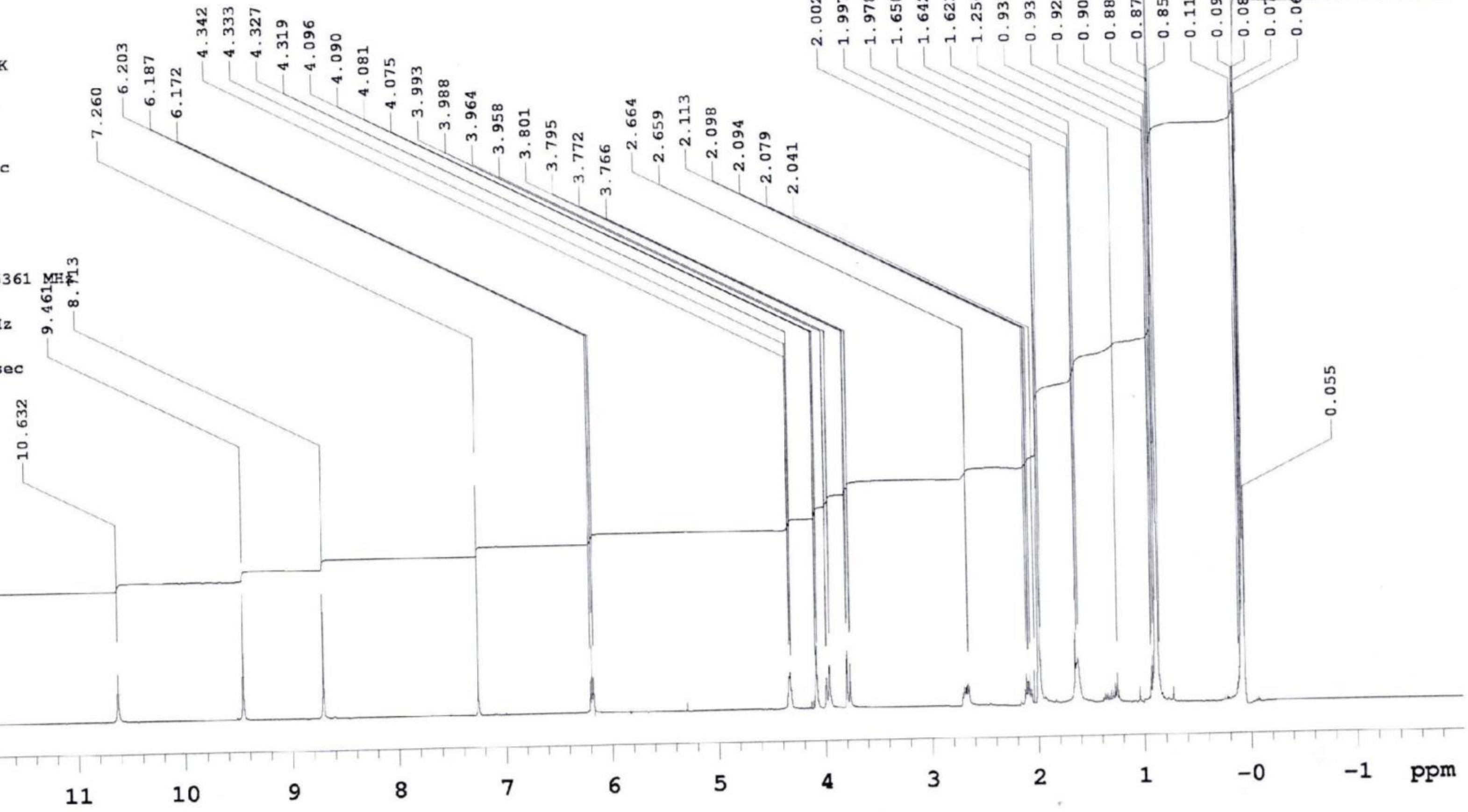

A. Chentsova

Sample: AC6-MP9

Pulse Sequence: s2pul

Solvent: cdcl3
Temp. 26.0 C / 299.1 K
Operator: vnmr1
Mercury-300BB "jutta"

Relax. delay 1.000 sec
Pulse 45.0 degrees
Acq. time 1.301 sec
Width 18115.9 Hz
1232 repetitions
OBSERVE C13, 75.4555928 MHz
DECOUPLE H1, 300.0831467 MHz
Power 37 dB
continuously on
WALTZ-16 modulated
DATA PROCESSING
Line broadening 1.0 Hz
FT size 65536
Total time 1 hr, 24 min, 1 sec

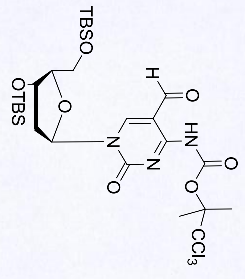

4

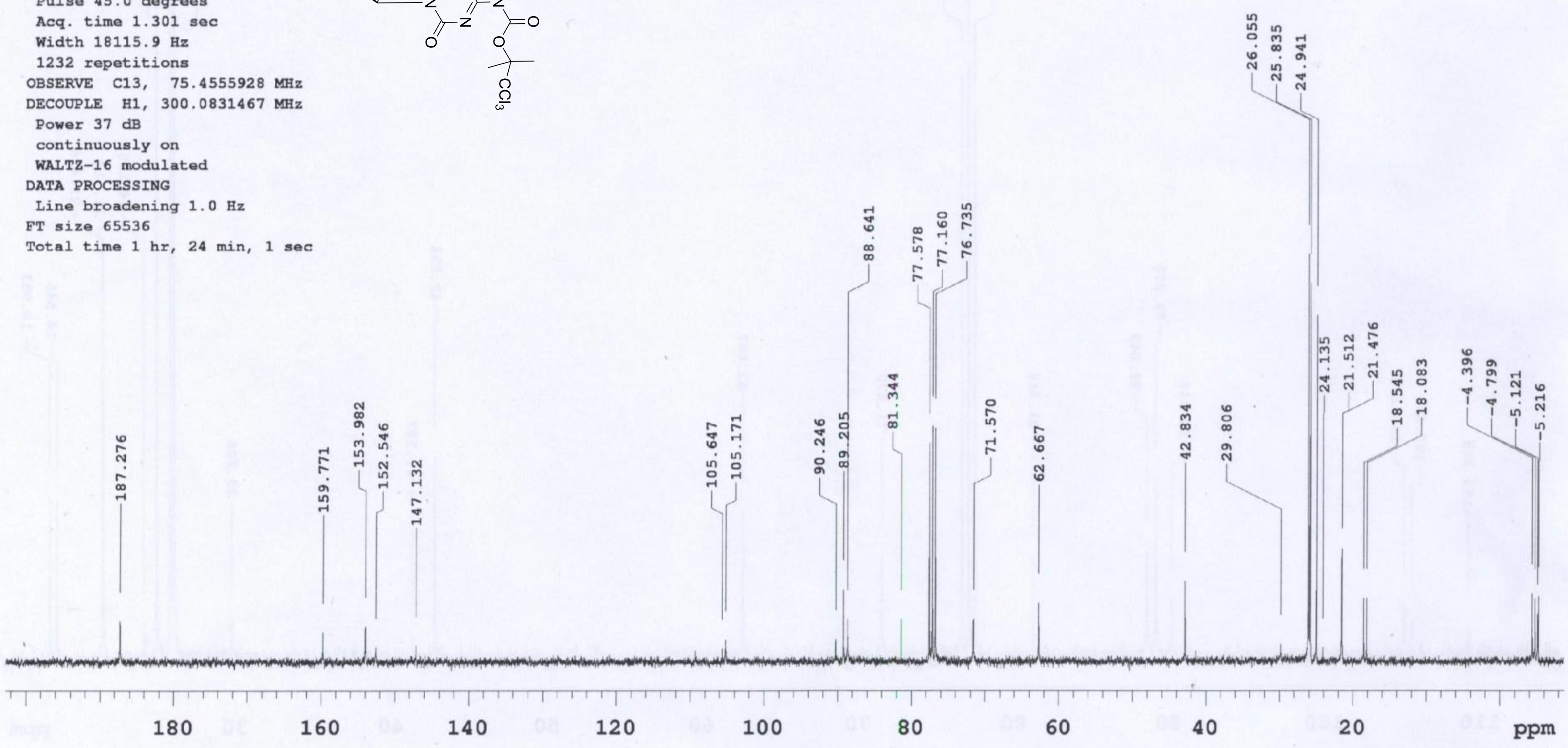

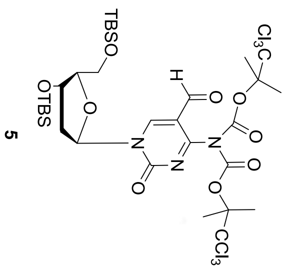

TBSO
OTBS
H
O
N
CCl3
Cl3C

5

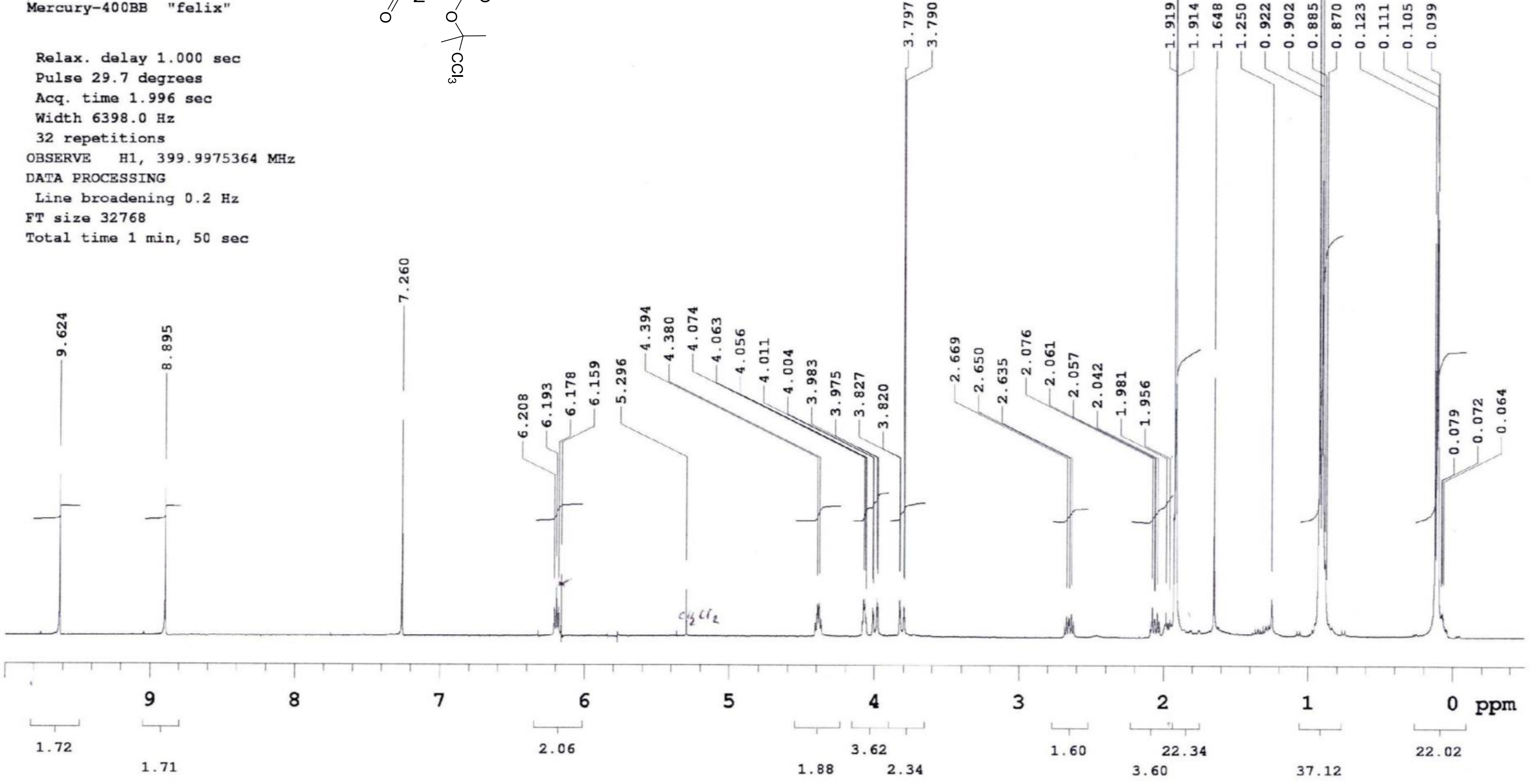

A. Chentsova
Sample: AC5-UP
Pulse Sequence: s2pul
Date: May 16 2012
Solvent: cdcl3
Temp. 26.0 C / 299.1 K
Operator: walkup
Mercury-400BB "felix"
Relax. delay 1.000 sec
Pulse 29.7 degrees
Acq. time 1.996 sec
Width 6398.0 Hz
32 repetitions
OBSERVE H1, 399.9975364 MHz
DATA PROCESSING
Line broadening 0.2 Hz
FT size 32768
Total time 1 min, 50 sec
9.624
8.895
7.260
6.208
6.193
6.178
6.159
5.296
4.394
4.380
4.074
4.063
4.056
4.011
4.004
3.983
3.975
3.827
3.820
3.797
3.790
2.669
2.650
2.635
2.076
2.061
2.057
2.042
1.981
1.956
1.919
1.914
1.648
1.250
0.922
0.902
0.885
0.870
0.123
0.111
0.105
0.099
0.079
0.072
0.064
9
8
7
6
5
4
3
2
1
0 ppm
1.72
1.71
2.06
1.88
3.62
2.34
1.60
3.60
22.34
37.12
22.02

Chentsova

Sample: AC 5-13A3

Pulse Sequence: APT
Date: May 10 2012

Solvent: cdcl3
Temp. 26.0 C / 299.1 K
Operator: walkup
Mercury-400BB "felix"

Relax. delay 1.000 sec
1st pulse 90.0 degrees
2nd pulse 135.0 degrees
Acq. time 1.000 sec
Width 24154.6 Hz
768 repetitions
OBSERVE C13, 100.5734443 MHz
DECOUPLE H1, 399.9994929 MHz
Power 38 dB
on during acquisition
WALTZ-16 modulated
DATA PROCESSING
Line b[illegible]
FT size 65536
Total time 1 hr, 15 min, 20 sec

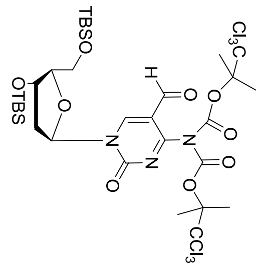

5

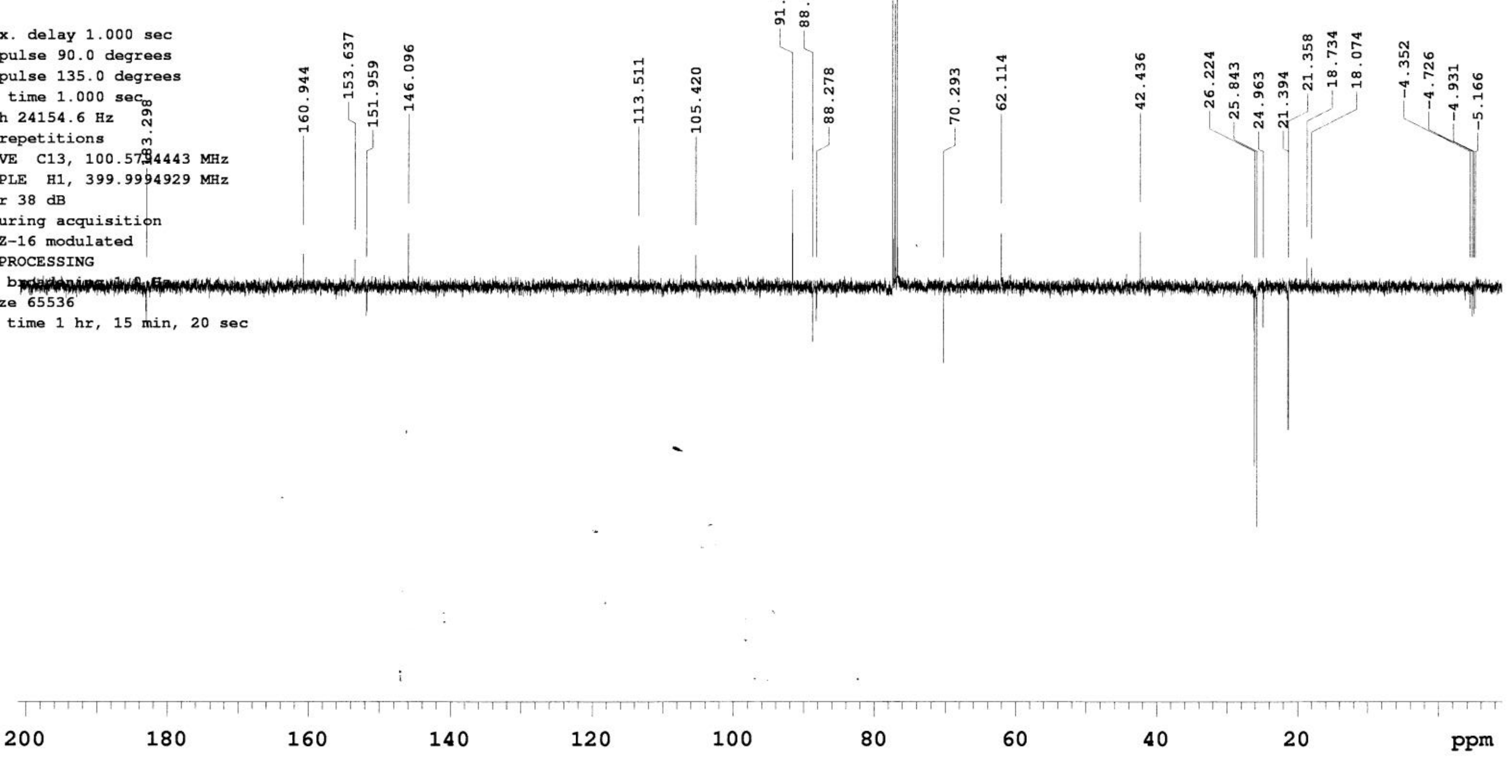

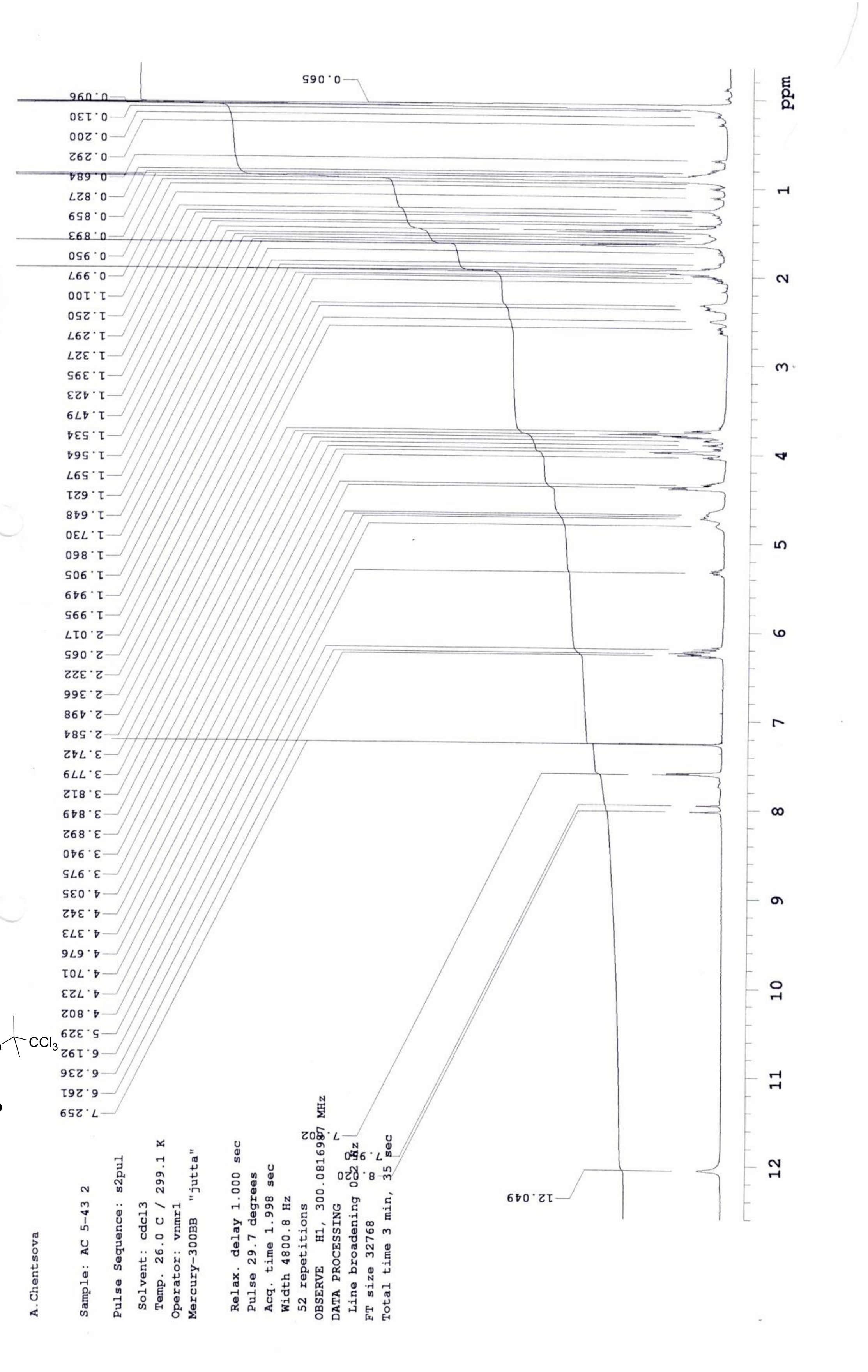
A.Chentsova
Sample: AC 5-43 2
Pulse Sequence: s2pul
Solvent: cdcl3
Temp. 26.0 C / 299.1 K
Operator: vnmr1
Mercury-300BB "jutta"
Relax. delay 1.000 sec
Pulse 29.7 degrees
Acq. time 1.998 sec
Width 4800.8 Hz
52 repetitions
OBSERVE H1, 300.0816997 MHz
DATA PROCESSING
Line broadening 0.2 Hz
FT size 32768
Total time 3 min, 35 sec
0.065
0.096
0.130
0.200
0.292
0.684
0.827
0.859
0.893
0.950
0.997
1.100
1.250
1.297
1.327
1.395
1.423
1.479
1.534
1.564
1.597
1.621
1.648
1.730
1.860
1.905
1.949
1.995
2.017
2.065
2.322
2.366
2.498
2.584
3.742
3.779
3.812
3.849
3.892
3.940
3.975
4.035
4.342
4.373
4.676
4.701
4.723
4.802
5.329
6.192
6.236
6.261
7.259
12.049
ppm
1
2
3
4
5
6
7
8
9
10
11
12

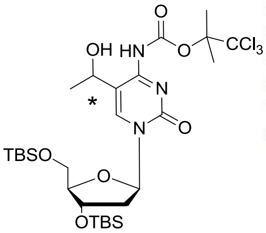
O
OH
HN
O
CCl_3
*
N
N
O
TBSO
O
OTBS

6a

A.Chentsova

Sample: AC5-434

Pulse Sequence: s2pul

Solvent: cdcl3
Temp. 26.0 C / 299.1 K
Operator: vnmr1
Mercury-300BB "jutta"

Relax. delay 1.000 sec
Pulse 29.7 degrees
Acq. time 1.998 sec
Width 4800.8 Hz
32 repetitions
OBSERVE H1, 300.0816988 MHz
DATA PROCESSING
Line broadening 0.1 Hz
FT size 32768
Total time 1 min, 47 sec

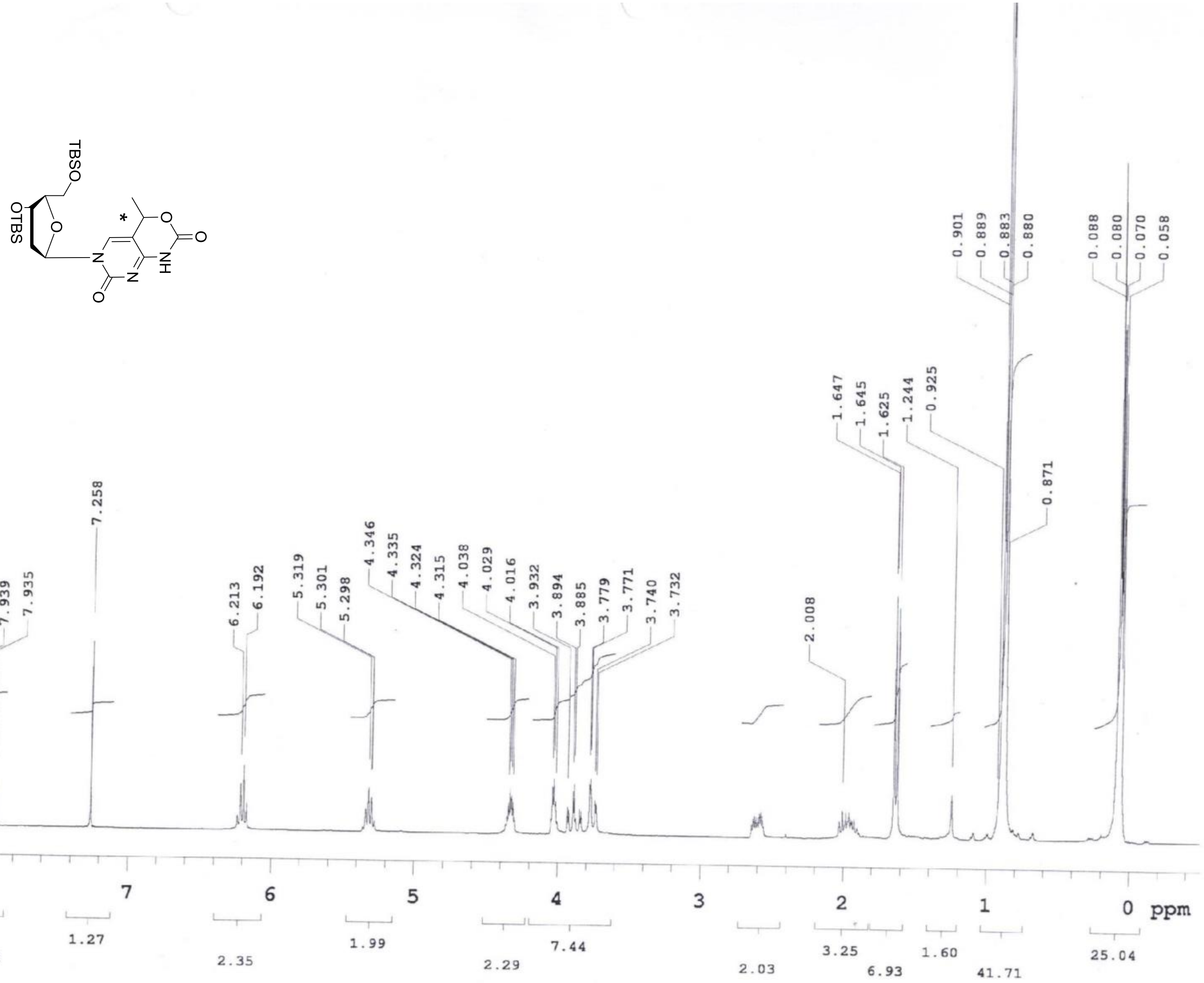

Std Carbon experiment
A.Chentsova

Sample: AC5-434

Pulse Sequence: s2pul

Solvent: cdcl3
Temp. 26.0 C / 299.1 K
Operator: vnmr1
Mercury-300BB "jutta"

Relax. delay 1.000 sec
Pulse 45.0 degrees
Acq. time 1.301 sec
Width 18115.9 Hz
2000 repetitions
OBSERVE C13, 75.4555916 MHz
DECOUPLE H1, 300.0831467 MHz
Power 37 dB
continuously on
WALTZ-16 modulated
DATA PROCESSING
Line broadening 1.0 Hz
FT size 65536
Total time 1 hr, 24 min, 1 sec

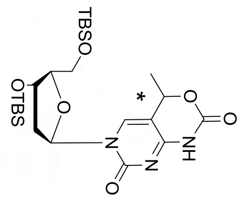

7a

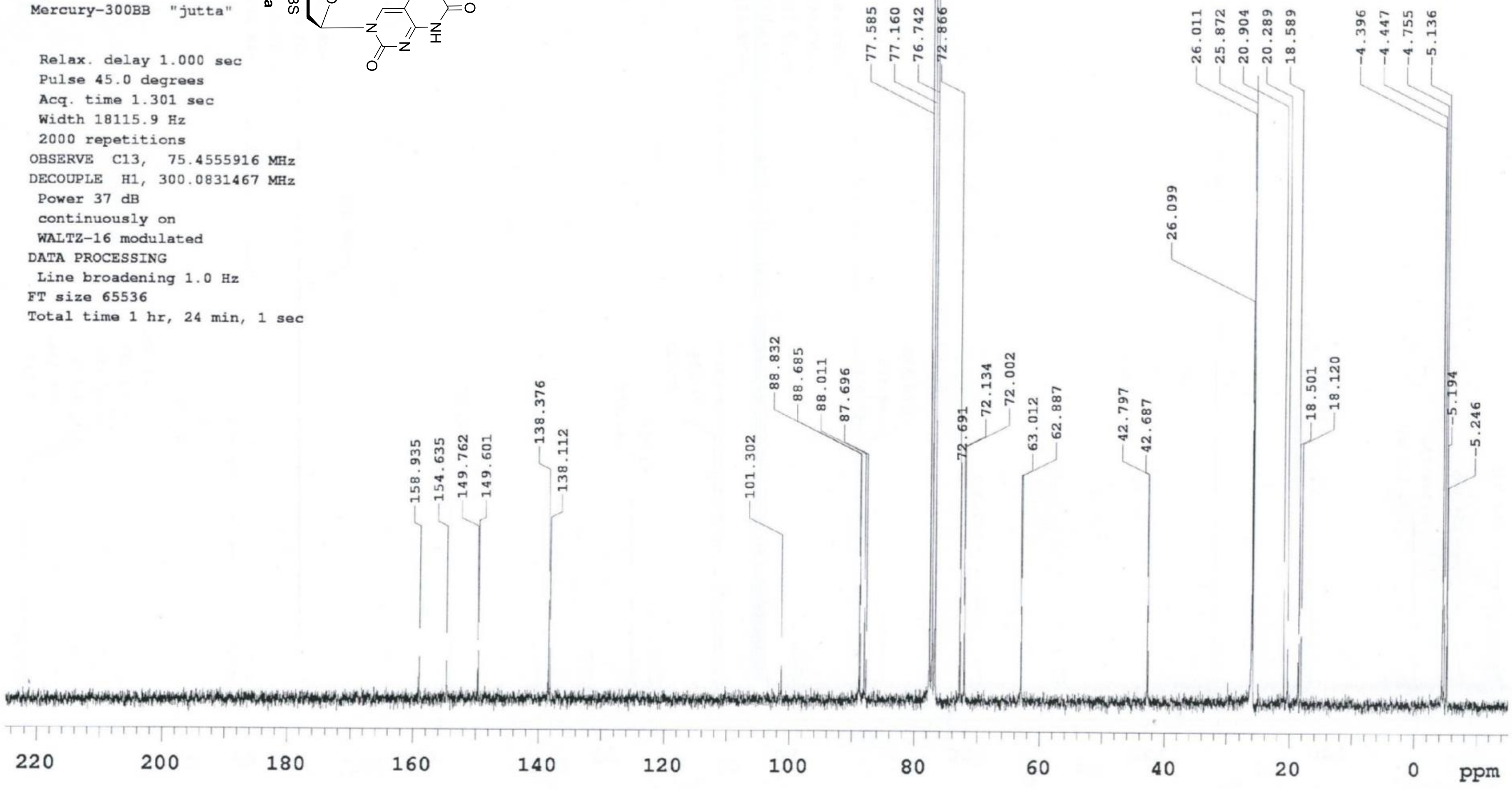

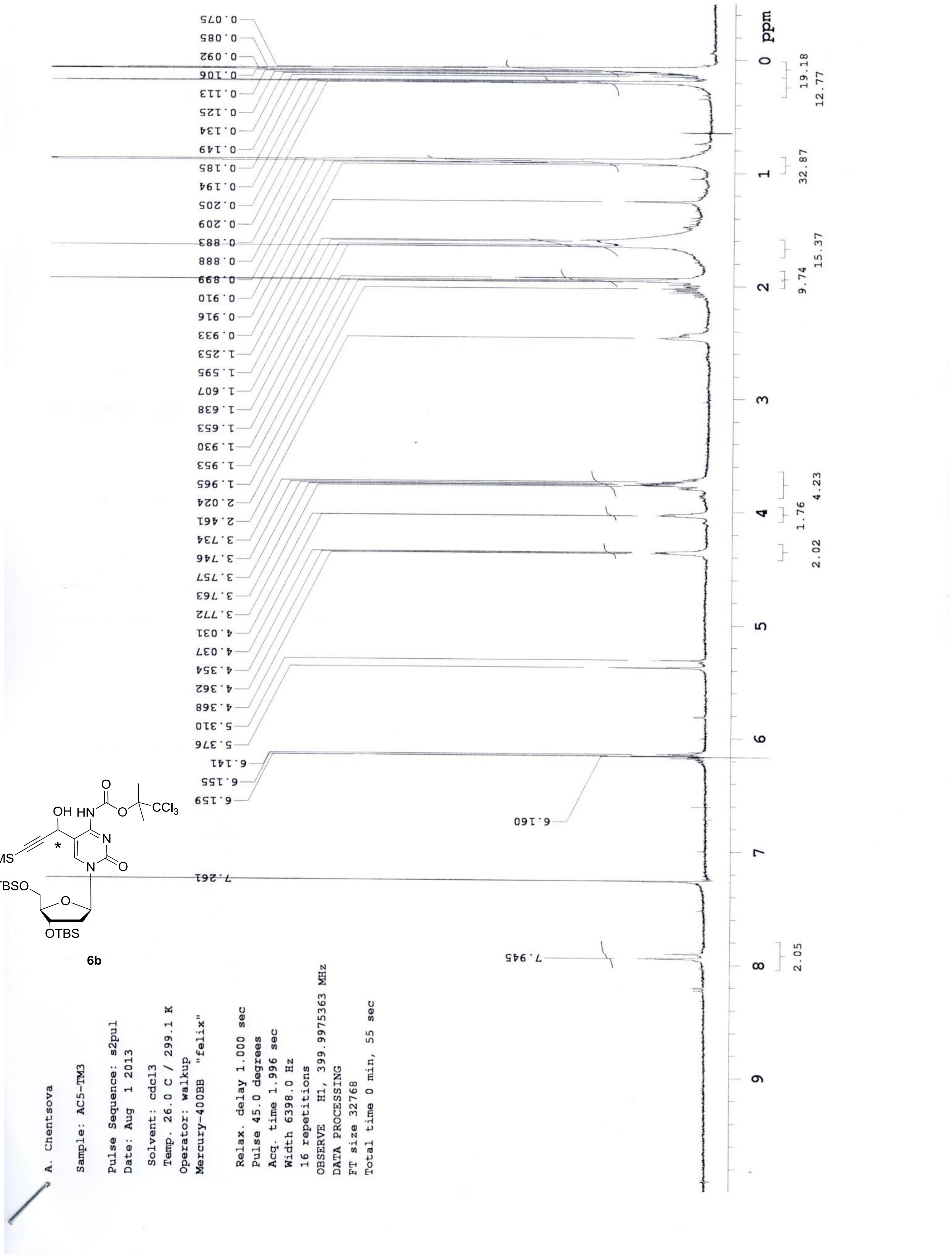
A. Chentsova
Sample: AC5-TM3
Pulse Sequence: s2pul
Date: Aug 1 2013
Solvent: cdcl3
Temp. 26.0 C / 299.1 K
Operator: walkup
Mercury-400BB "felix"
Relax. delay 1.000 sec
Pulse 45.0 degrees
Acq. time 1.996 sec
Width 6398.0 Hz
16 repetitions
OBSERVE H1, 399.9975363 MHz
DATA PROCESSING
FT size 32768
Total time 0 min, 55 sec
0.075
0.085
0.092
0.106
0.113
0.125
0.134
0.149
0.185
0.194
0.205
0.209
0.883
0.888
0.899
0.910
0.916
0.933
1.253
1.595
1.607
1.638
1.653
1.930
1.953
1.965
2.024
2.461
3.734
3.746
3.757
3.763
3.772
4.031
4.037
4.354
4.362
4.368
5.310
5.376
6.141
6.155
6.159
6.160
7.261
7.945
19.18
12.77
32.87
9.74
15.37
2.02
1.76
4.23
2.05
ppm
TMS
OH
HN
O
CCl3
N
TBSO
OTBS

6b

Chentsova

Sample: AC 5-26A

Pulse Sequence: APT
Date: Sep 6 2012

Solvent: cdcl3
Temp. 26.0 C / 299.1 K
Operator: walkup
Mercury-400BB "felix"

Relax. delay 1.000 sec
1st pulse 90.0 degrees
2nd pulse 135.0 degrees
Acq. time 1.000 sec
Width 24154.6 Hz
2784 repetitions
OBSERVE C13, 100.5794443 MHz
DECOUPLE H1, 399.9994929 MHz
Power 38 dB
on during acquisition
WALTZ-16 modulated
DATA PROCESSING
Line broadening 1.0 Hz
FT size 65536
Total time 3 hr, 7 min, 20 sec

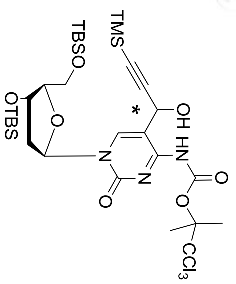

6b

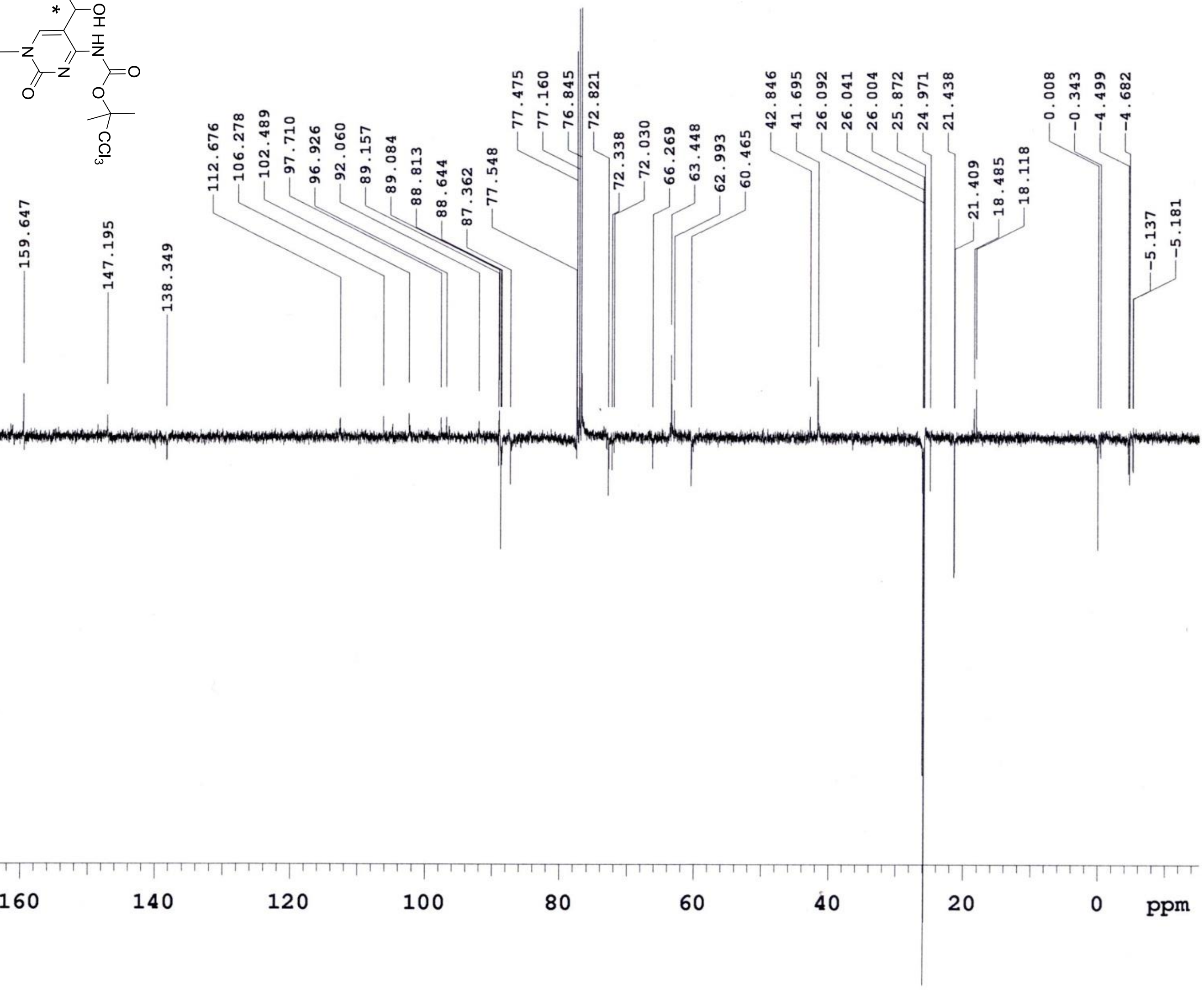

[illegible]entsova

Sample: AC5-Li3

Pulse Sequence: s2pul

Solvent: cdcl3
Temp. 26.0 C / 299.1 K
Operator: vnmr1
Mercury-300BB "jutta"

Relax. delay 1.000 sec
Pulse 29.7 degrees
Acq. time 1.998 sec
Width 4800.8 Hz
24 repetitions
OBSERVE H1, 300.0816984 MHz
DATA PROCESSING
Line broadening 0.2 Hz
FT size 32768
Total time 3 min, 35 sec

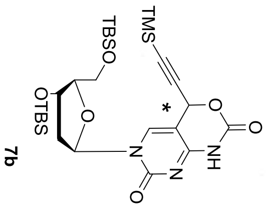

7b

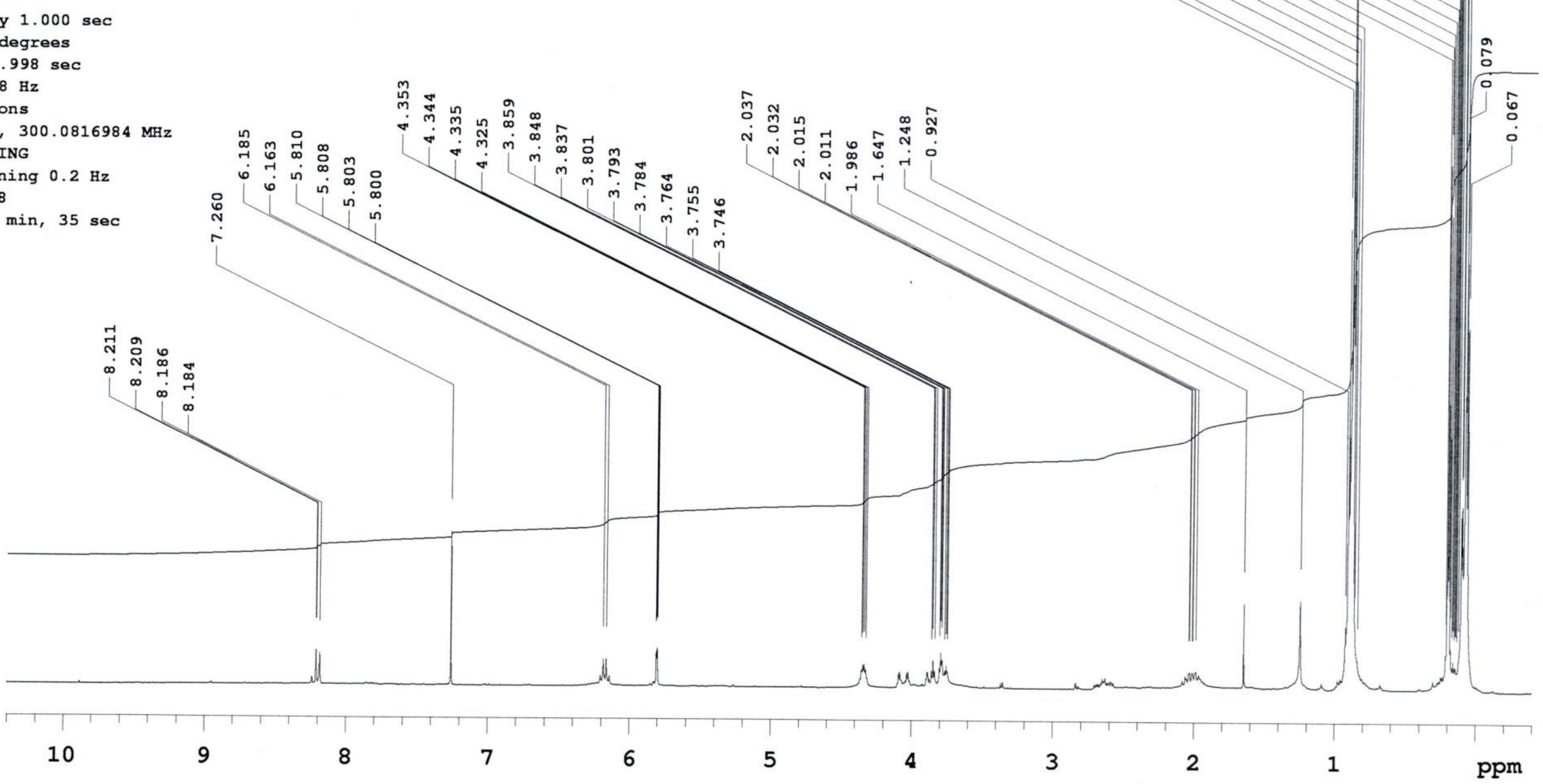

Std Carbon experiment

Sample: AC5-L3

Pulse Sequence: s2pul

Solvent: cdcl3
Temp. 26.0 C / 299.1 K
Operator: vnmr1
Mercury-300BB "jutta"

Relax. delay 1.000 sec
Pulse 45.0 degrees
Acq. time 1.301 sec
Width 18115.9 Hz
8000 repetitions
OBSERVE C13, 75.4555916 MHz
DECOUPLE H1, 300.0831467 MHz
Power 37 dB
continuously on
WALTZ-16 modulated
DATA PROCESSING
Line broadening 1.0 Hz
FT size 65536
Total time 5 hr, 23 min, 28 sec

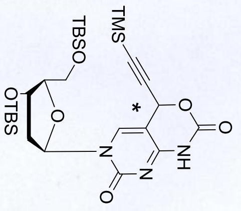

7b

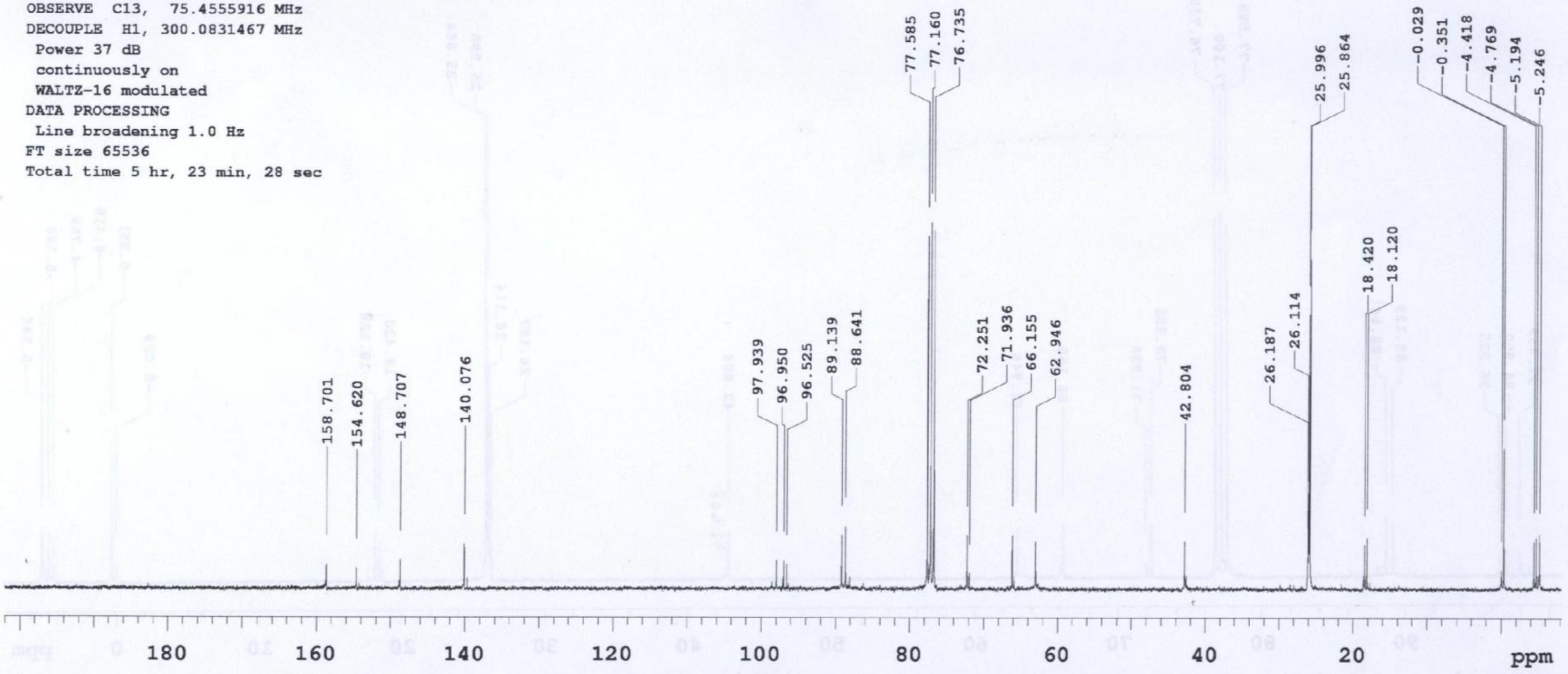

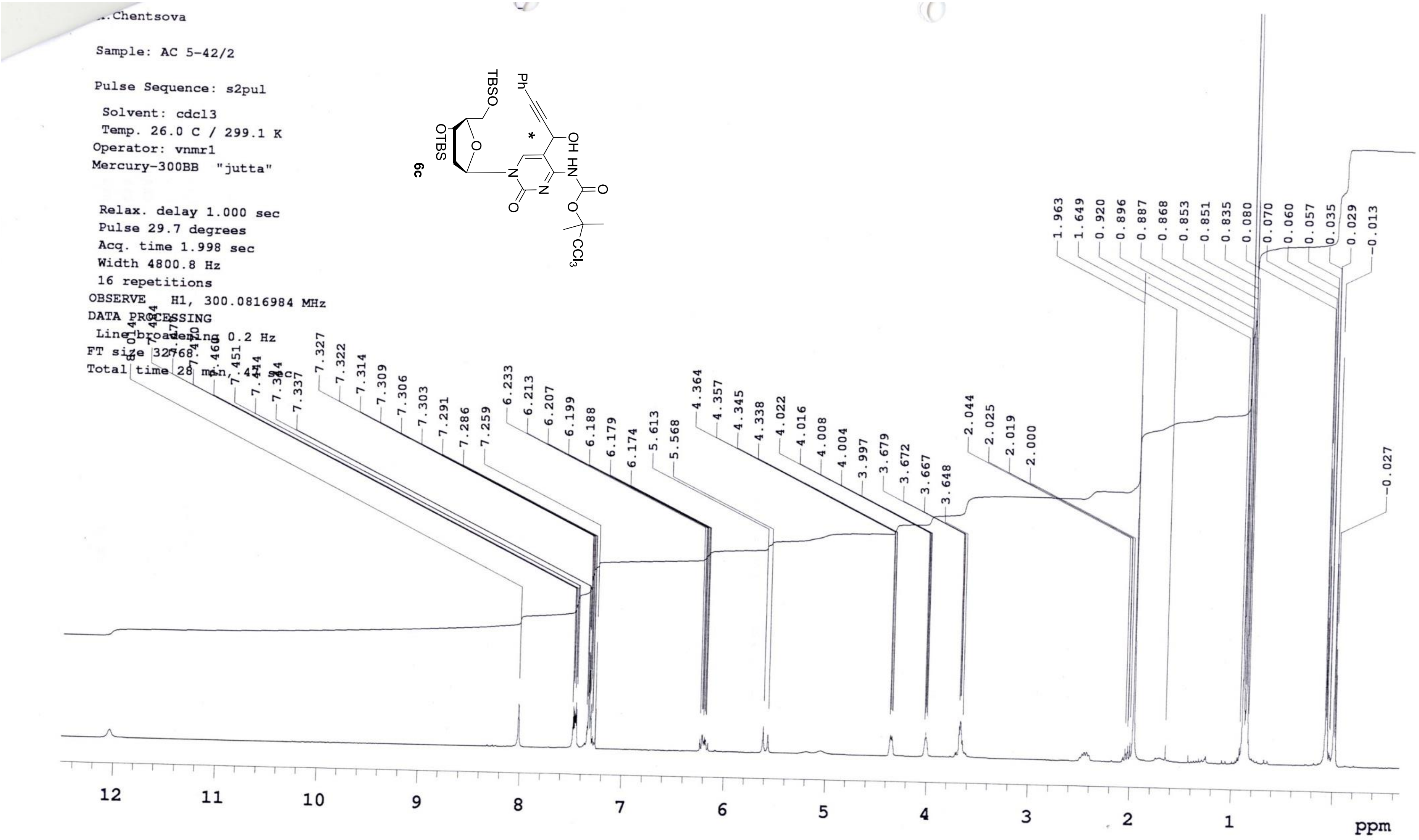
..Chentsova
Sample: AC 5-42/2
Pulse Sequence: s2pul
Solvent: cdcl3
Temp. 26.0 C / 299.1 K
Operator: vnmr1
Mercury-300BB "jutta"
Relax. delay 1.000 sec
Pulse 29.7 degrees
Acq. time 1.998 sec
Width 4800.8 Hz
16 repetitions
OBSERVE H1, 300.0816984 MHz
DATA PROCESSING
Line broadening 0.2 Hz
FT size 32768
Total time 28 min, 44 sec
7.337
7.327
7.322
7.314
7.309
7.306
7.303
7.291
7.286
7.259
6.233
6.213
6.207
6.199
6.188
6.179
6.174
5.613
5.568
4.364
4.357
4.345
4.338
4.022
4.016
4.008
4.004
3.997
3.679
3.672
3.667
3.648
2.044
2.025
2.019
2.000
1.963
1.649
0.920
0.896
0.887
0.868
0.853
0.851
0.835
0.080
0.070
0.060
0.057
0.035
0.029
-0.013
-0.027
12
11
10
9
8
7
6
5
4
3
2
1
ppm
TBSO
OTBS
Ph
OH
HN
N
O
CCl3
*
6c

A.Chentsova

Sample: AC 5-422

Pulse Sequence: s2pul
Date: Jan 14 2013

Solvent: cdcl3
Temp. 26.0 C / 299.1 K
Operator: walkup
Mercury-400BB "felix"

Relax. delay 1.000 sec
Pulse 45.0 degrees
Acq. time 1.300 sec
Width 24154.6 Hz
1024 repetitions
OBSERVE C13, 100.5794466 MHz
DECOUPLE H1, 399.9994929 MHz
Power 38 dB
continuously on
WALTZ-16 modulated
DATA PROCESSING
Line broadening 0.5 Hz
FT size 65536
Total time 1 hr, 25 min, 53 sec

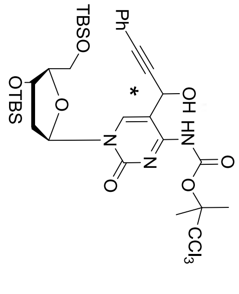

6c

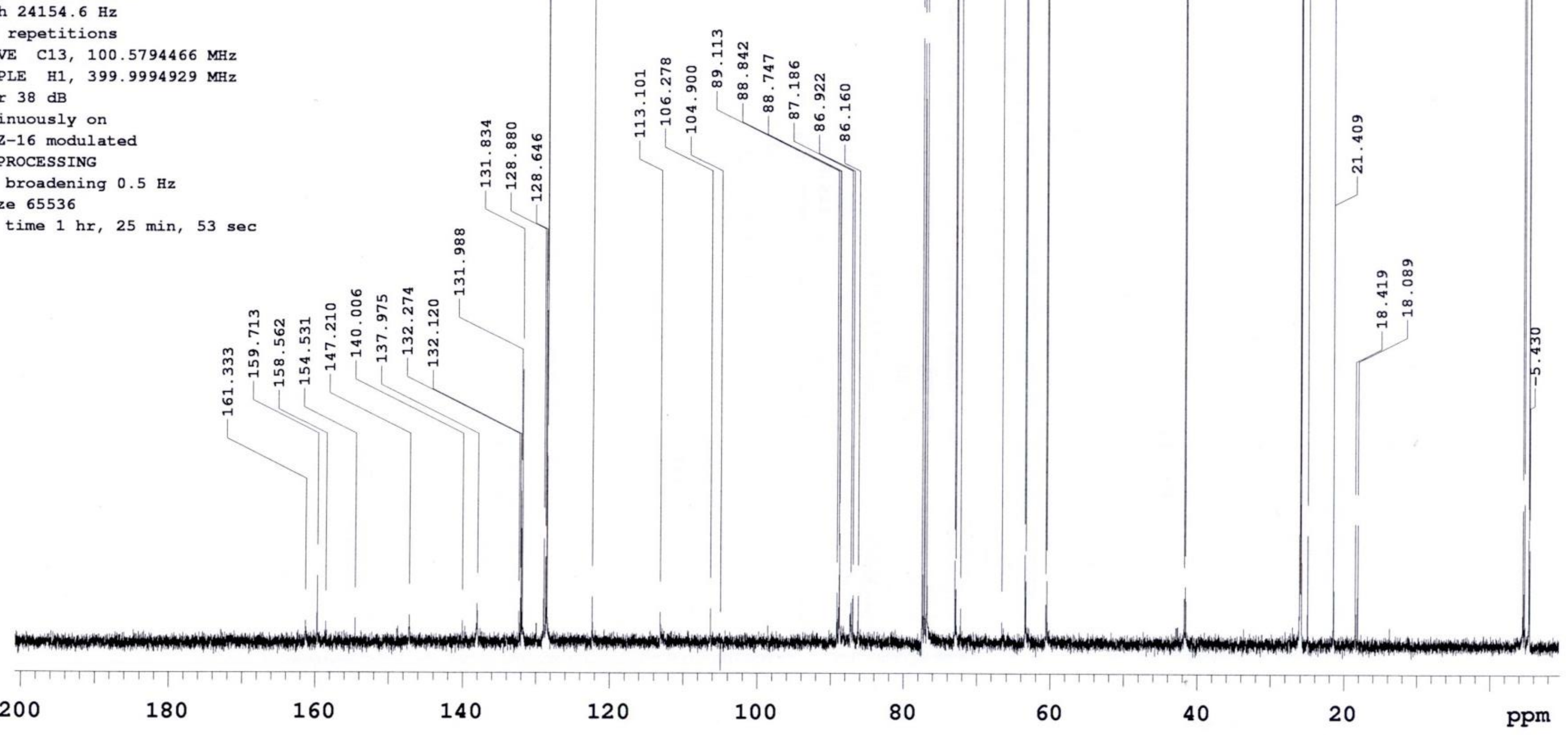

Chentsova

Sample: AC5-424A

Pulse Sequence: s2pul

Solvent: cdcl3
Temp. 26.0 C / 299.1 K
Operator: vnmr1
Mercury-300BB "jutta"

Relax. delay 1.000 sec
Pulse 29.7 degrees
Acq. time 1.998 sec
Width 4800.8 Hz
16 repetitions
OBSERVE H1, 300.0816981 MHz
DATA PROCESSING
Line broadening 0.2 Hz
FT size 32768
Total time 1 min, 47 sec

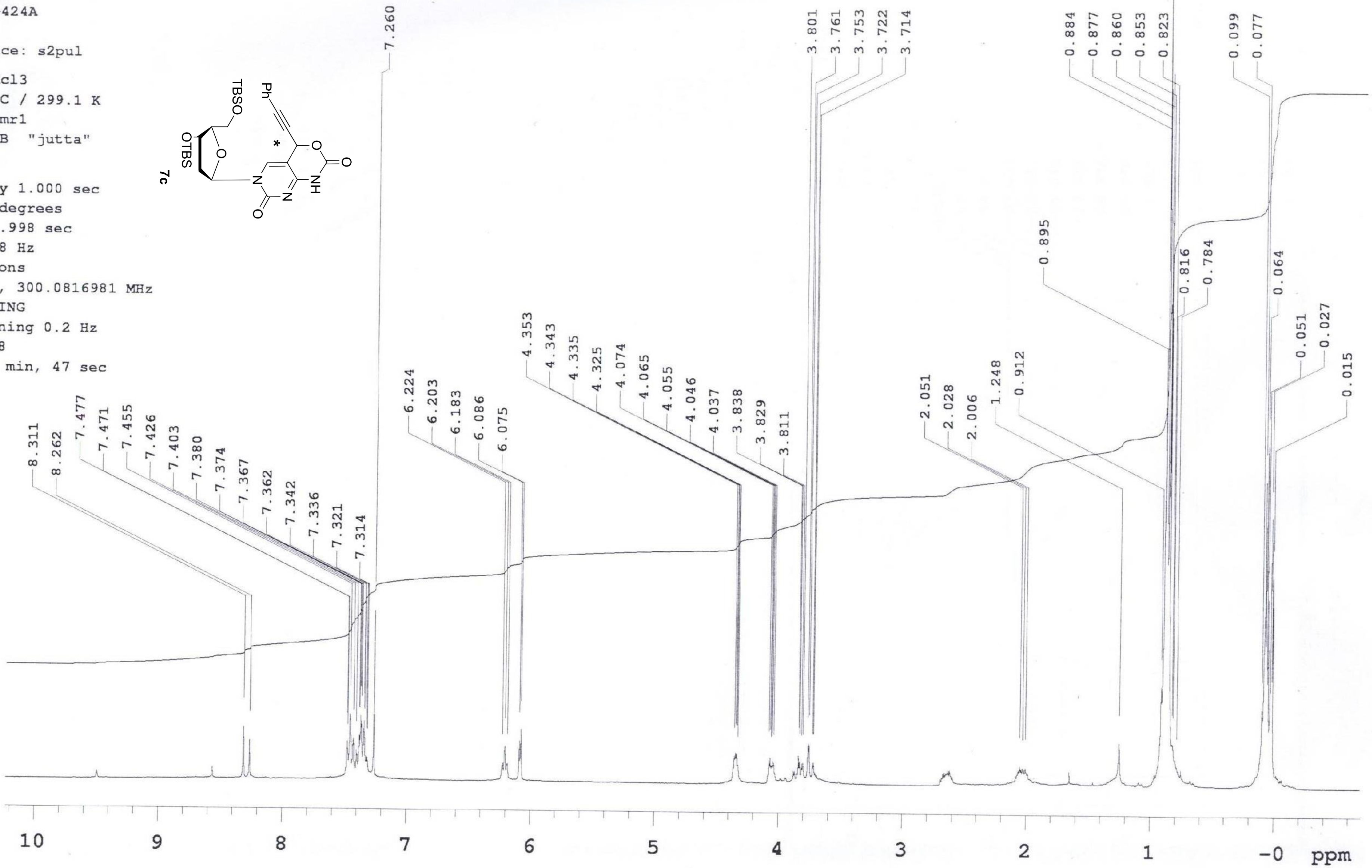

A. Chentsova

Sample: AC5-424A

Pulse Sequence: s2pul
Date: Apr 9 2013

Solvent: cdcl3
Temp. 26.0 C / 299.1 K
Operator: walkup
Mercury-400BB "felix"

Relax. delay 1.000 sec
Pulse 45.0 degrees
Acq. time 1.300 sec
Width 24154.6 Hz
912 repetitions
OBSERVE C13, 100.5794466 MHz
DECOUPLE H1, 399.9994929 MHz
Power 38 dB
continuously on
WALTZ-16 modulated
DATA PROCESSING
Line broadening 0.5 Hz
FT size 65536
Total time 42 min, 58 sec

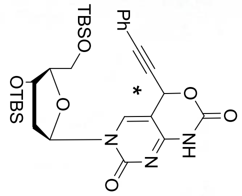

7c

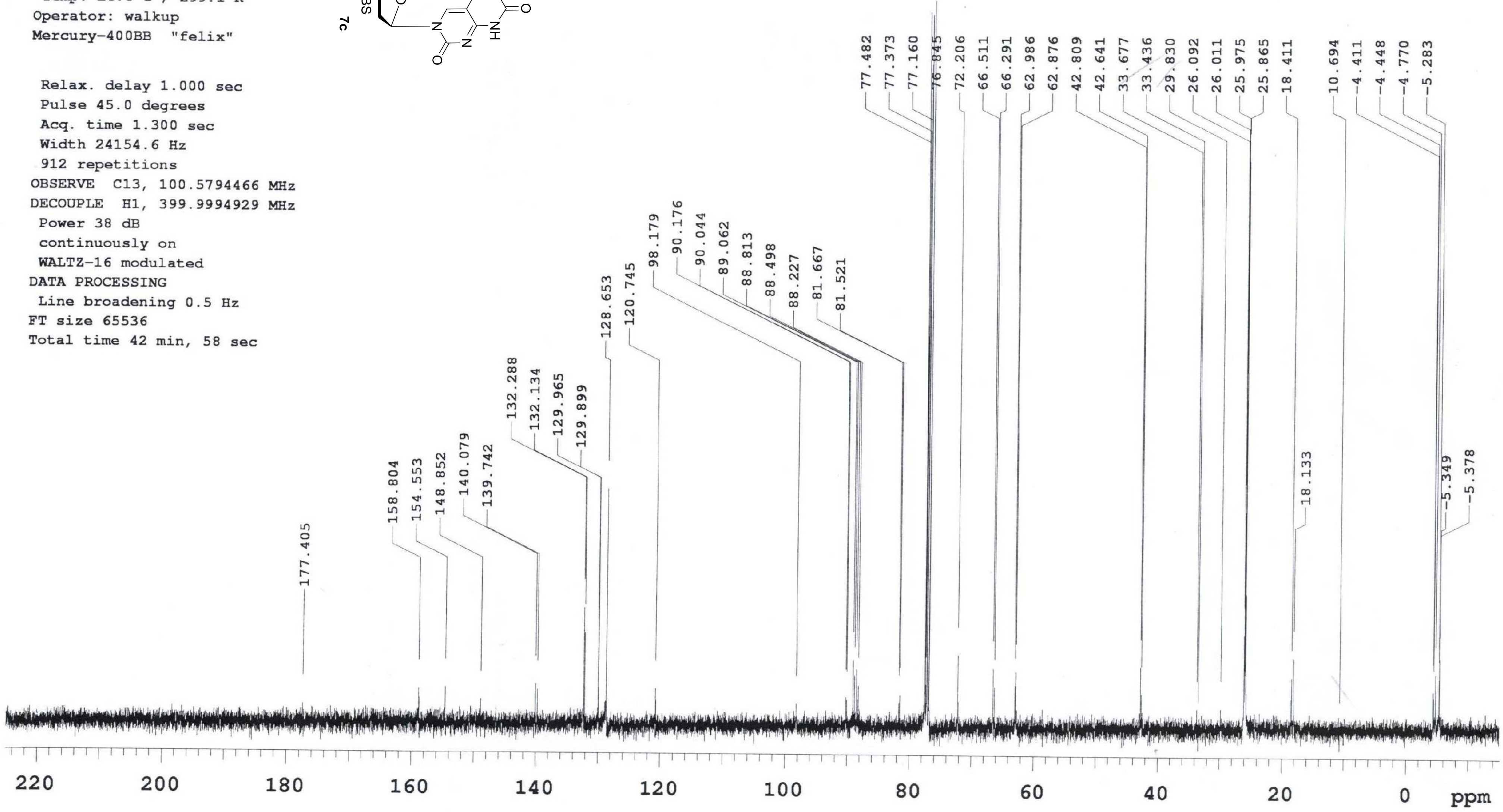

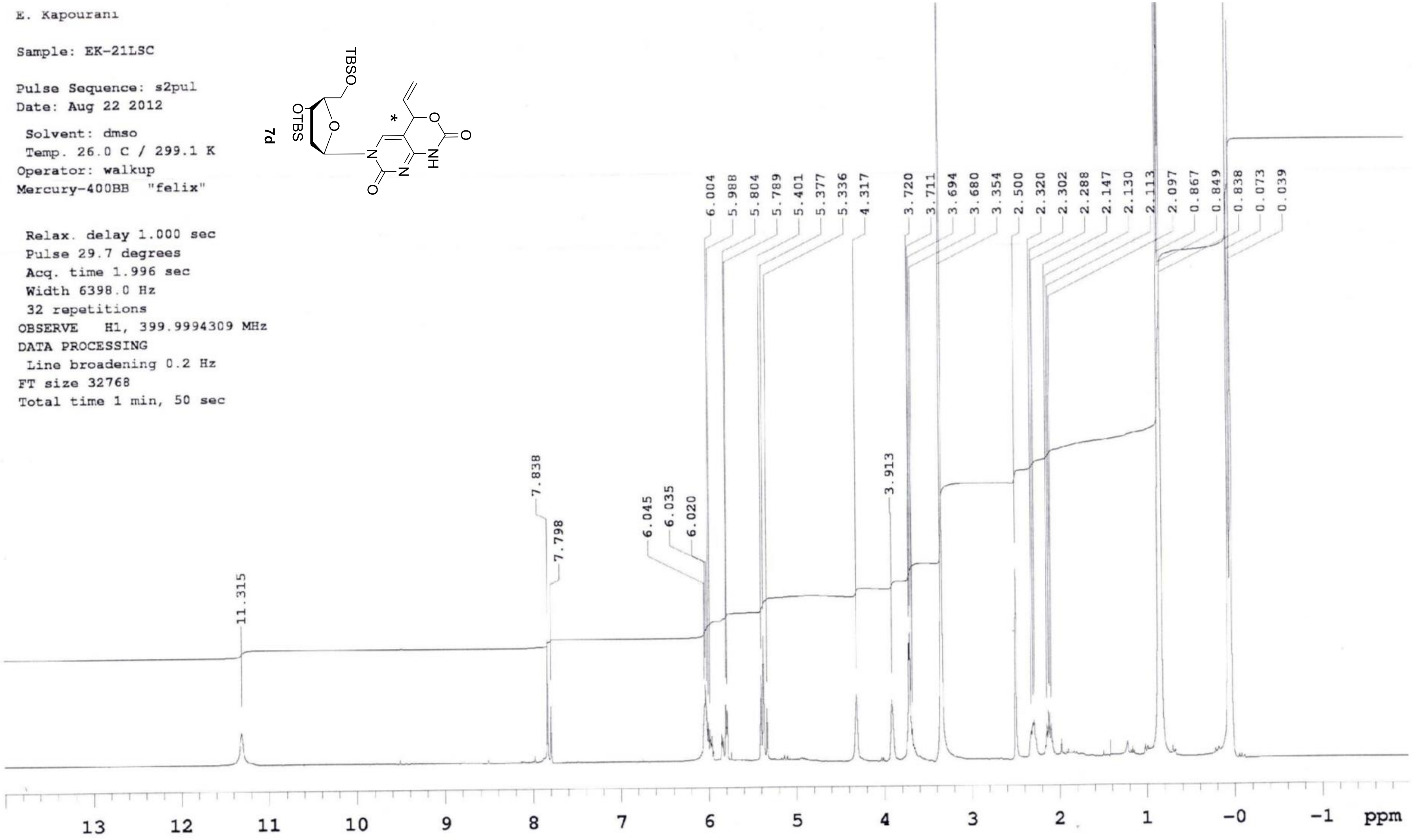

E. Kapourani
Sample: EK-21LSC
Pulse Sequence: s2pul
Date: Aug 22 2012
Solvent: dmso
Temp. 26.0 C / 299.1 K
Operator: walkup
Mercury-400BB "felix"
Relax. delay 1.000 sec
Pulse 29.7 degrees
Acq. time 1.996 sec
Width 6398.0 Hz
32 repetitions
OBSERVE H1, 399.9994309 MHz
DATA PROCESSING
Line broadening 0.2 Hz
FT size 32768
Total time 1 min, 50 sec
11.315
7.838
7.798
6.045
6.035
6.020
6.004
5.988
5.804
5.789
5.401
5.377
5.336
4.317
3.913
3.720
3.711
3.694
3.680
3.354
2.500
2.320
2.302
2.288
2.147
2.130
2.113
2.097
0.867
0.849
0.838
0.073
0.039
13
12
11
10
9
8
7
6
5
4
3
2
1
-0
-1
ppm
TBSO
OTBS
O
N
N
NH
O
O
O
*

7d

E. Kapourani

Sample: EK-21LSC

Pulse Sequence: s2pul
Date: Aug 22 2012

Solvent: dmso
Temp. 26.0 C / 299.1 K
Operator: walkup
Mercury-400BB "felix"

Relax. delay 1.000 sec
Pulse 45.0 degrees
Acq. time 1.300 sec
Width 24154.6 Hz
10000 repetitions
OBSERVE C13, 100.5799830 MHz
DECOUPLE H1, 400.0013929 MHz
Power 38 dB
continuously on
WALTZ-16 modulated
DATA PROCESSING
Line broadening 1.0 Hz
FT size 65536
Total time 6 hr, 49 min, 3 sec

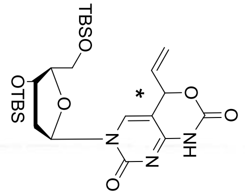

7d

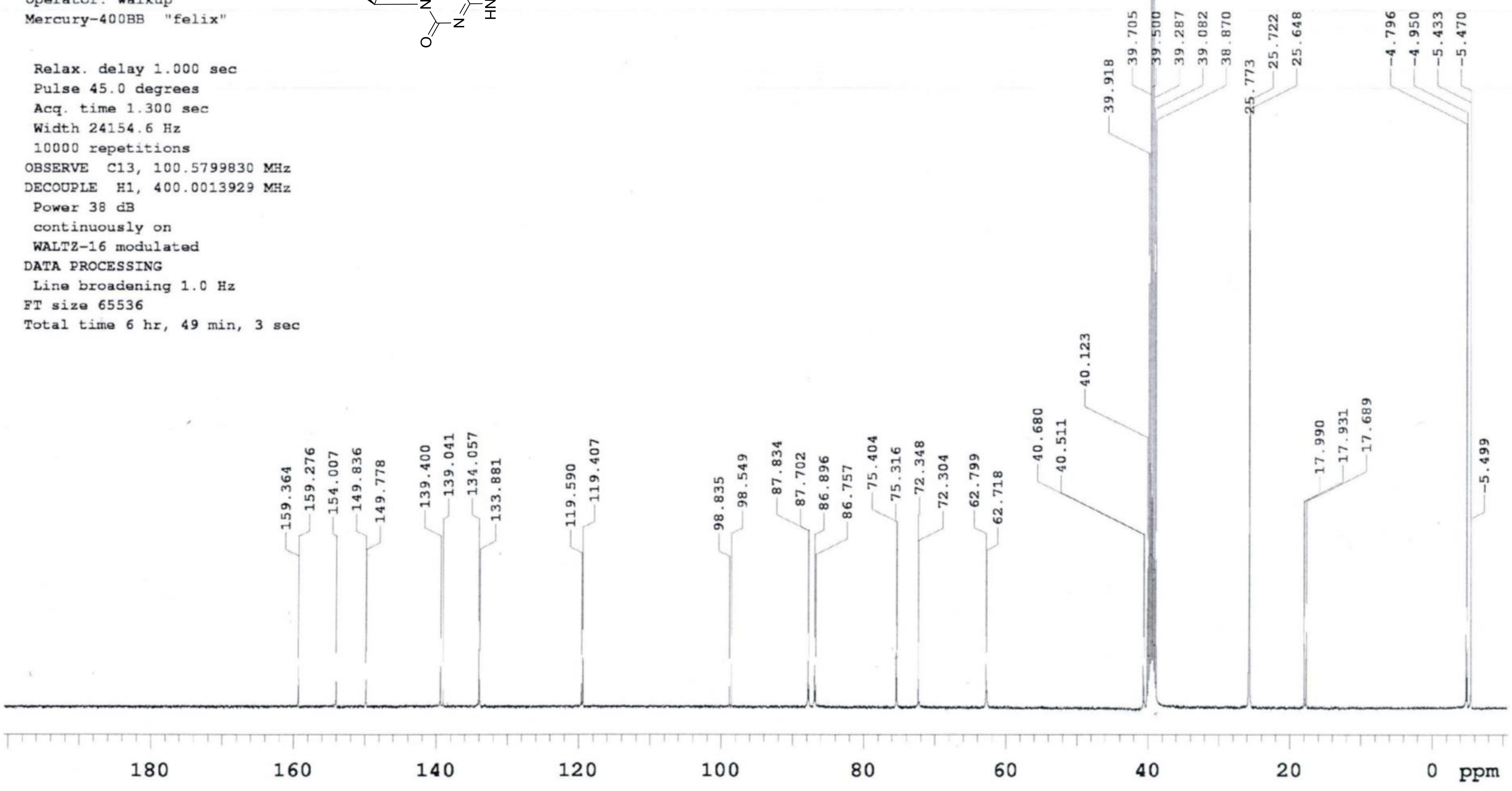

A. Chentsova

Sample: AC5-52A2

Pulse Sequence: s2pul

Solvent: cdcl3
Temp. 26.0 C / 299.1 K
Operator: vnmr1
Mercury-300BB "jutta"

Relax. delay 1.000 sec
Pulse 29.7 degrees
Acq. time 1.998 sec
Width 4800.8 Hz
32 repetitions
OBSERVE H1, 300.0816984 MHz
DATA PROCESSING
Line broadening 0.2 Hz
FT size 32768
Total time 1 min, 47 sec

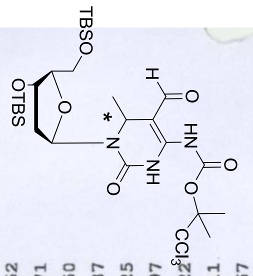

8a

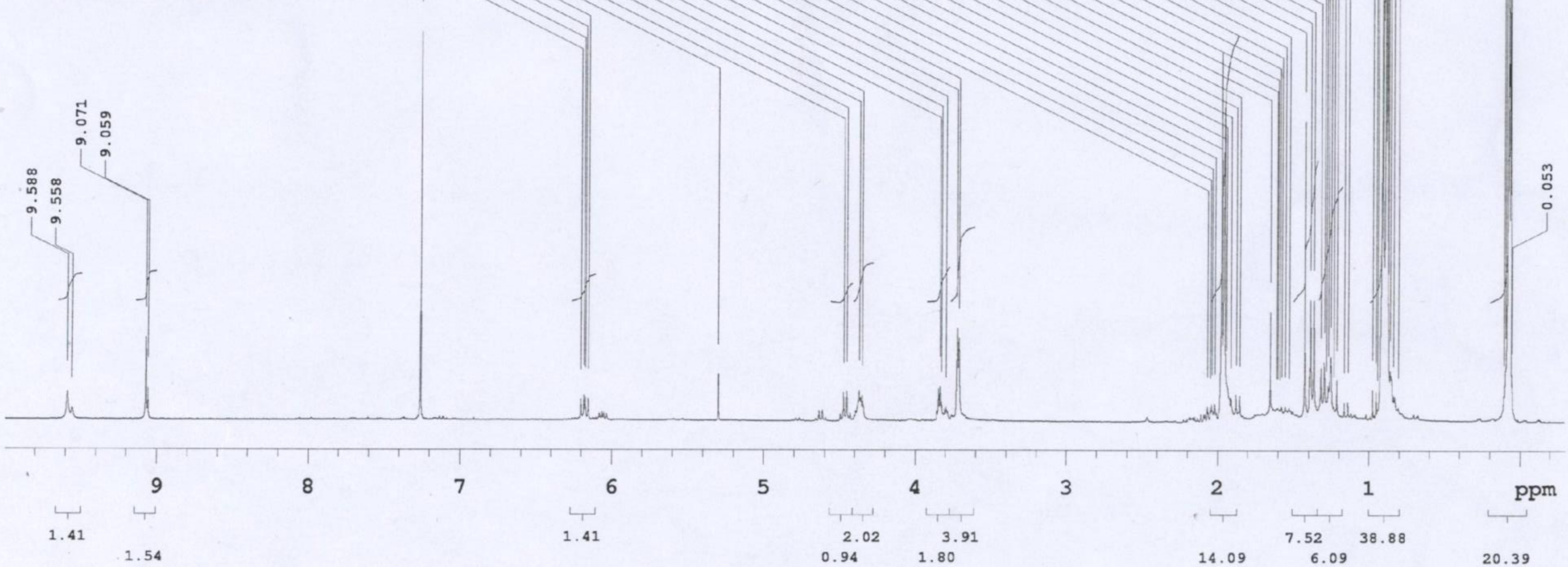

A. Chentsova

Sample: AC5-52A2

Pulse Sequence: s2pul

Solvent: cdcl3
Temp. 26.0 C / 299.1 K
Operator: vnmr1
Mercury-300BB "jutta"

Relax. delay 1.000 sec
Pulse 45.0 degrees
Acq. time 1.301 sec
Width 18115.9 Hz
20000 repetitions
OBSERVE C13, 75.4555905 MHz
DECOUPLE H1, 300.0831467 MHz
Power 37 dB
continuously on
WALTZ-16 modulated
DATA PROCESSING
Line broadening 0.5 Hz
FT size 65536
Total time 14 hr, 14 sec

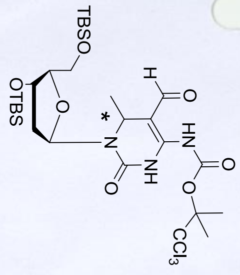

8a

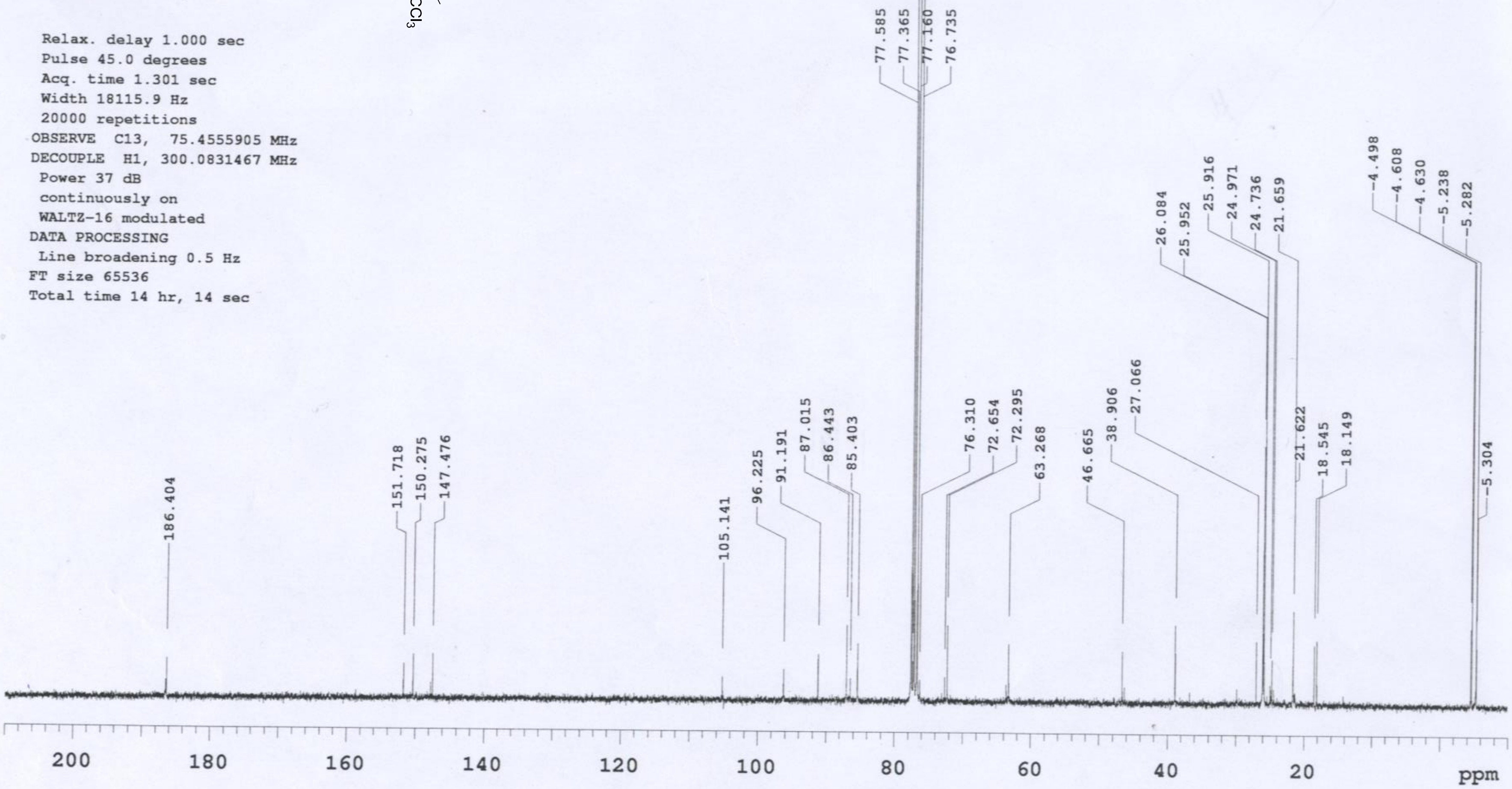

A. Chentsova

Sample: AC5-501

Pulse Sequence: s2pul
Date: Dec 19 2012

Solvent: cdcl3
Temp. 26.0 C / 299.1 K
Operator: walkup
Mercury-400BB "felix"

Relax. delay 1.000 sec
Pulse 29.7 degrees
Acq. time 1.996 sec
Width 6398.0 Hz
32 repetitions
OBSERVE H1, 399.9975363 MHz
DATA PROCESSING
Line broadening 0.2 Hz
FT size 32768
Total time 1 min, 50 sec

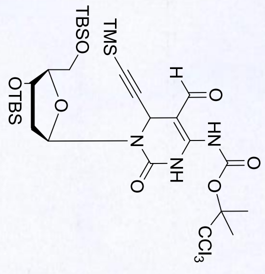

diastereomer 8b

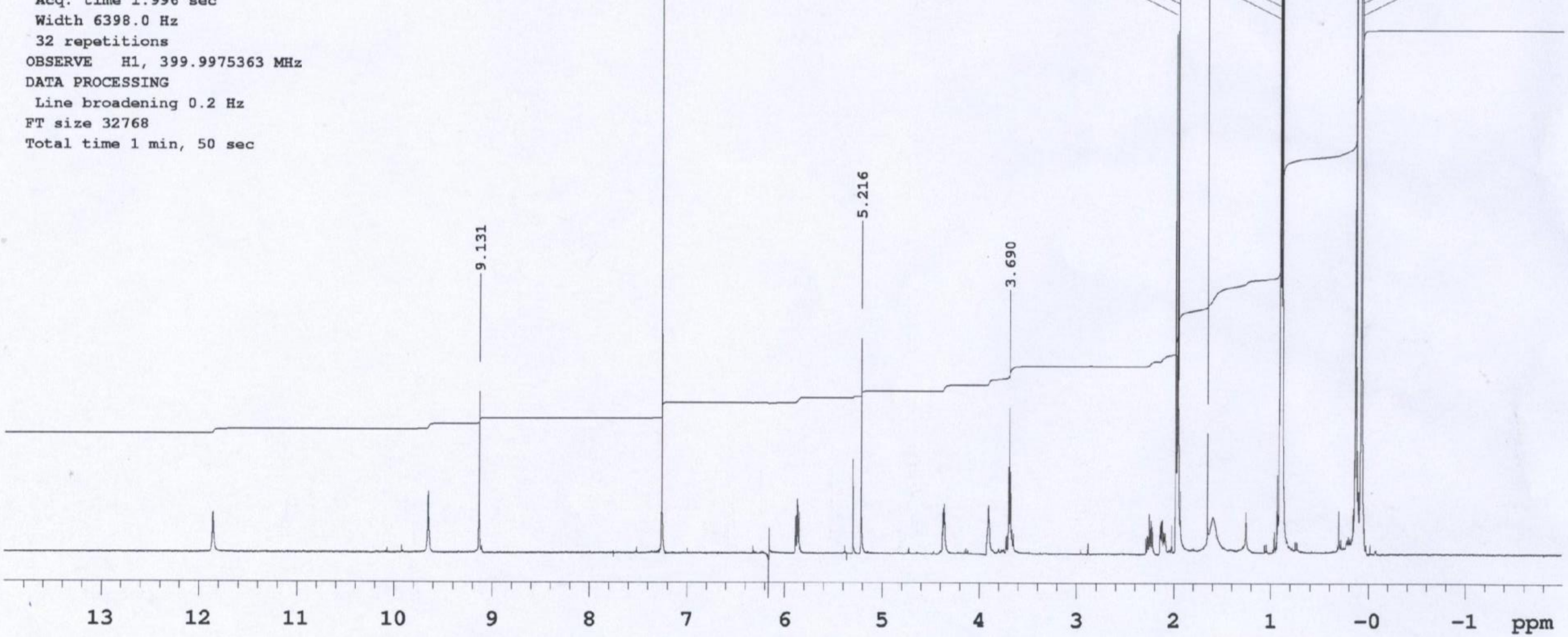

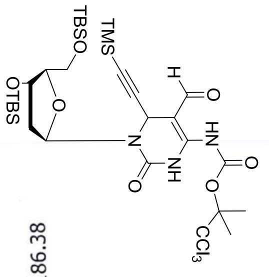

diastereomer 8b

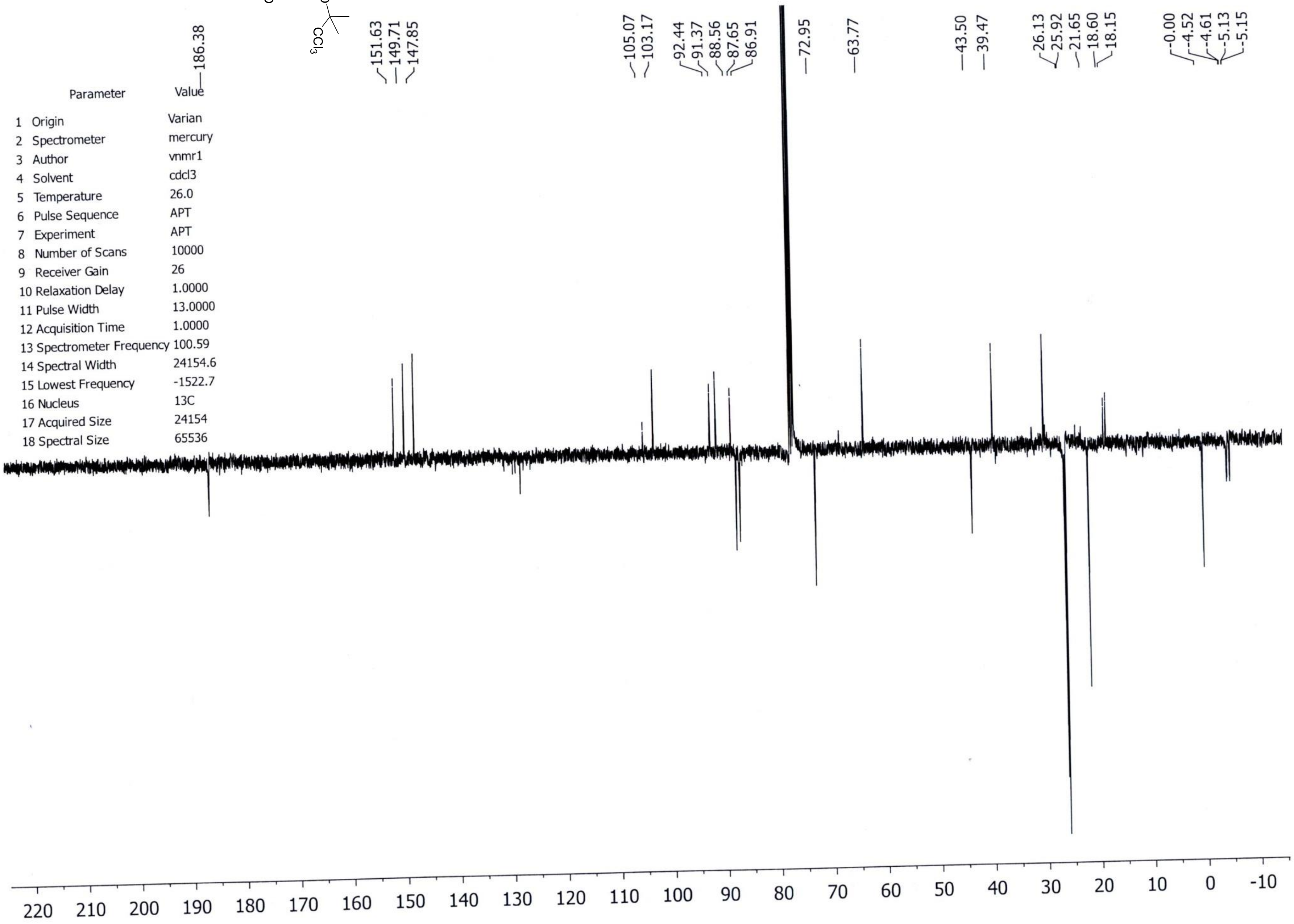

	Parameter	Value
1	Origin	Varian
2	Spectrometer	mercury
3	Author	vnmr1
4	Solvent	cdcl3
5	Temperature	26.0
6	Pulse Sequence	APT
7	Experiment	APT
8	Number of Scans	10000
9	Receiver Gain	26
10	Relaxation Delay	1.0000
11	Pulse Width	13.0000
12	Acquisition Time	1.0000
13	Spectrometer Frequency	100.59
14	Spectral Width	24154.6
15	Lowest Frequency	-1522.7
16	Nucleus	13C
17	Acquired Size	24154
18	Spectral Size	65536

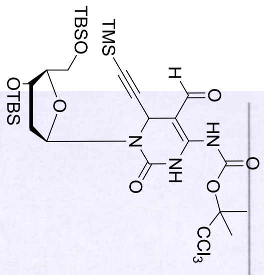

diastereomer 8b*

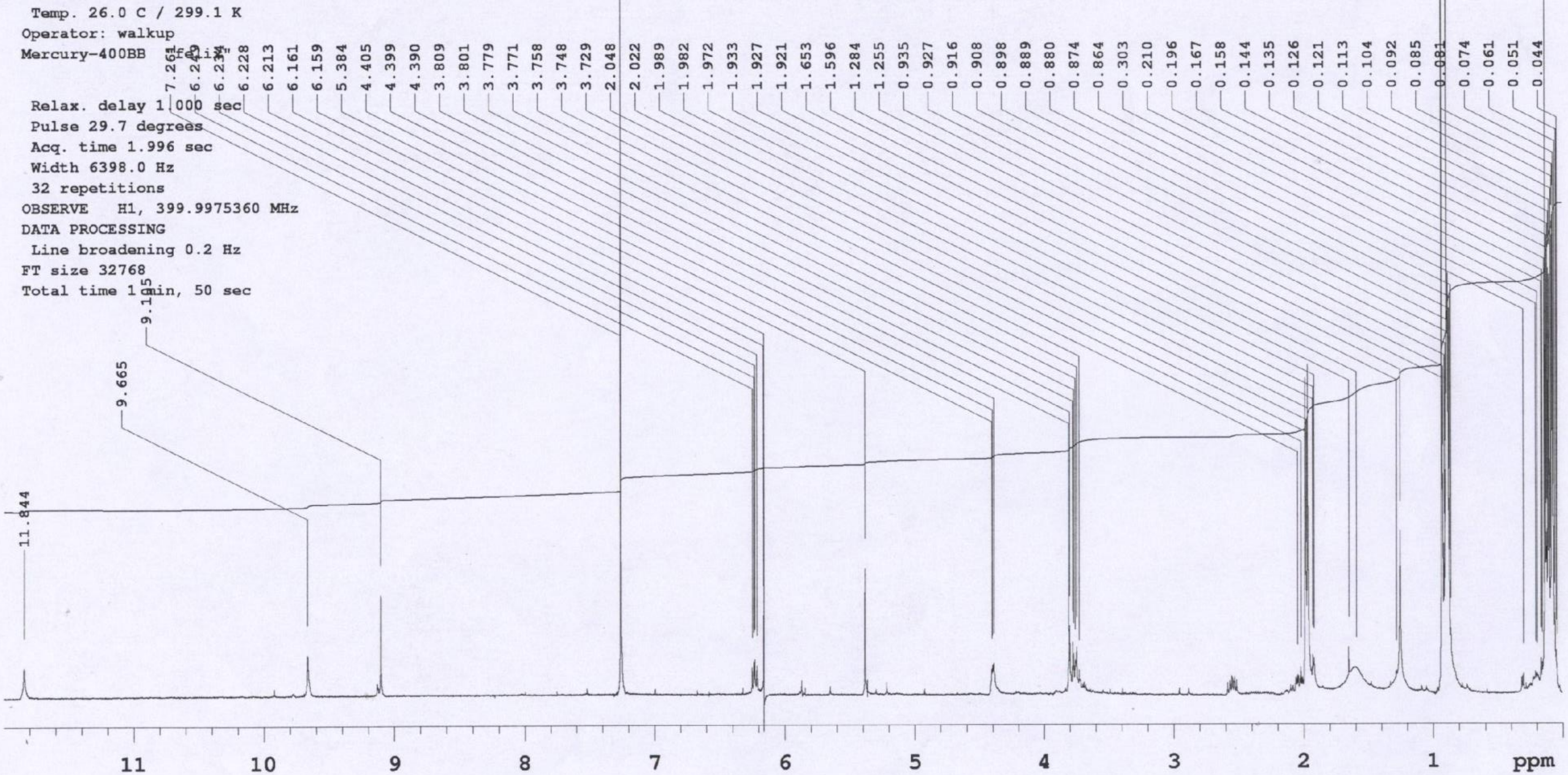

A. Chentsova

exp3 Carbon

SAMPLE		SPECIAL	
date	Dec 17 2012	temp	26.0
solvent	cdcl3	gain	20
file	exp	spin	20
ACQUISITION		hst	0.008
sw	24154.6	pw90	13.000
at	1.300	alfa	10.000
np	62826	FLAGS	
fb	13400	il	n
bs	32	in	n
d1	1.000	dp	y
nt	20000	hs	nn
ct	20000	PROCESSING	
TRANSMITTER		lb	1.00
tn	C13	fn	not used
sfrq	100.590	DISPLAY	
tof	1054.2	sp	-1522.8
tpwr	59	wp	24153.9
pw	6.500	rfl	9284.3
DECOUPLER		rfp	7760.7
dn	H1	rp	85.6
dof	0	lp	-149.0
dm	yyy	PLOT	
dmm	w	wc	250
dpwr	38	sc	0
dmf	9100	vs	5522
		th	3
		ai	cdc ph

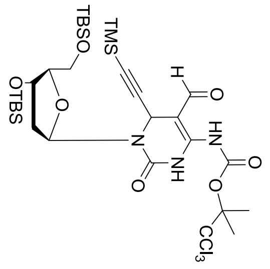

diastereomer 8b*

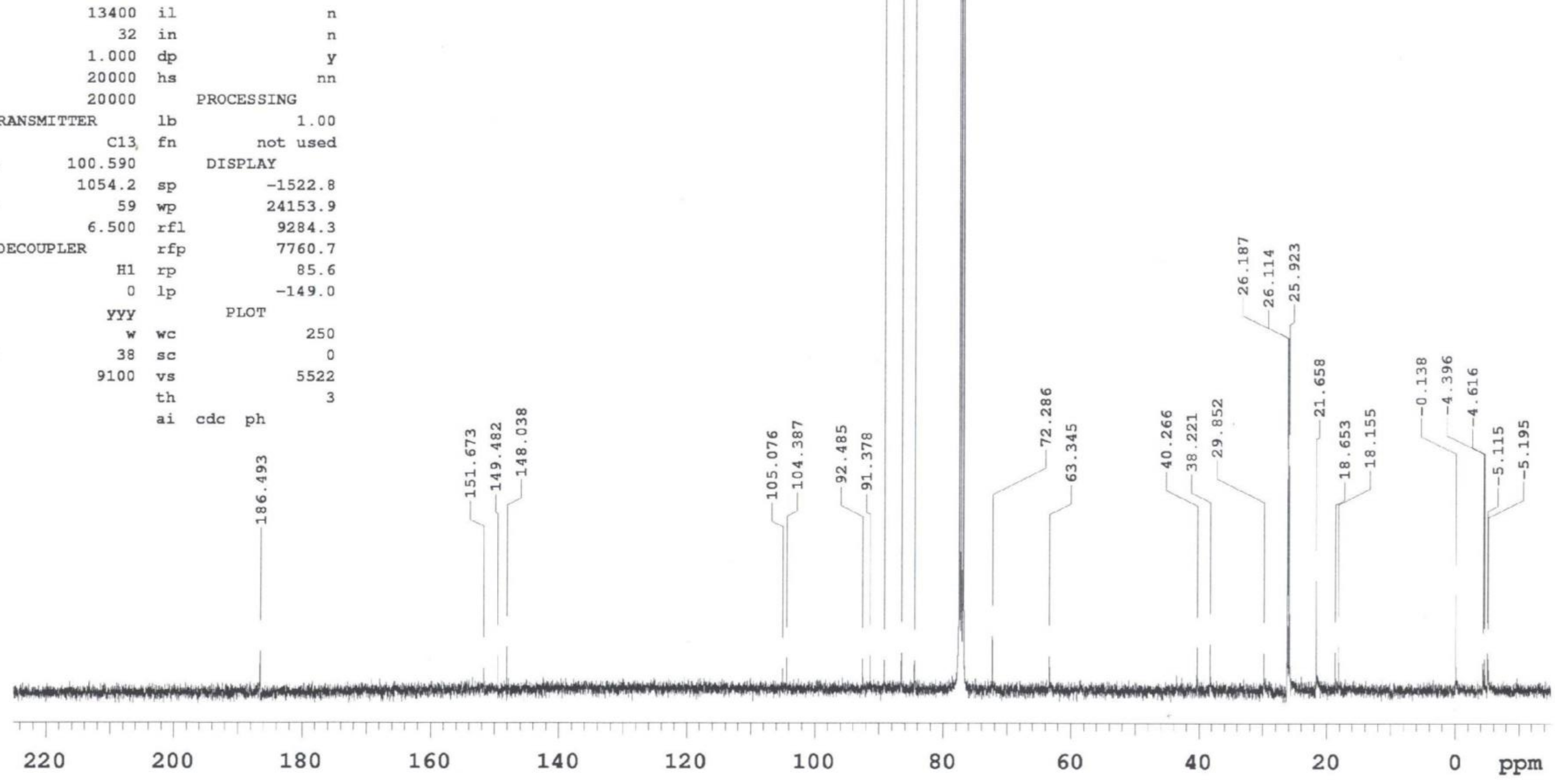

Sample: AC5-51A3

Pulse Sequence: s2pul

Solvent: cdcl3
Temp. 26.0 C / 299.1 K
Operator: vnmr1
Mercury-300BB "jutta"

Relax. delay 1.000 sec
Pulse 29.7 degrees
Acq. time 1.998 sec
Width 4800.8 Hz
24 repetitions
OBSERVE H1, 300.0816988 MHz
DATA PROCESSING
Line broadening 0.2 Hz
FT size 32768
Total time 1 min 47 sec

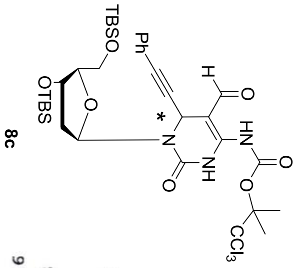

8c

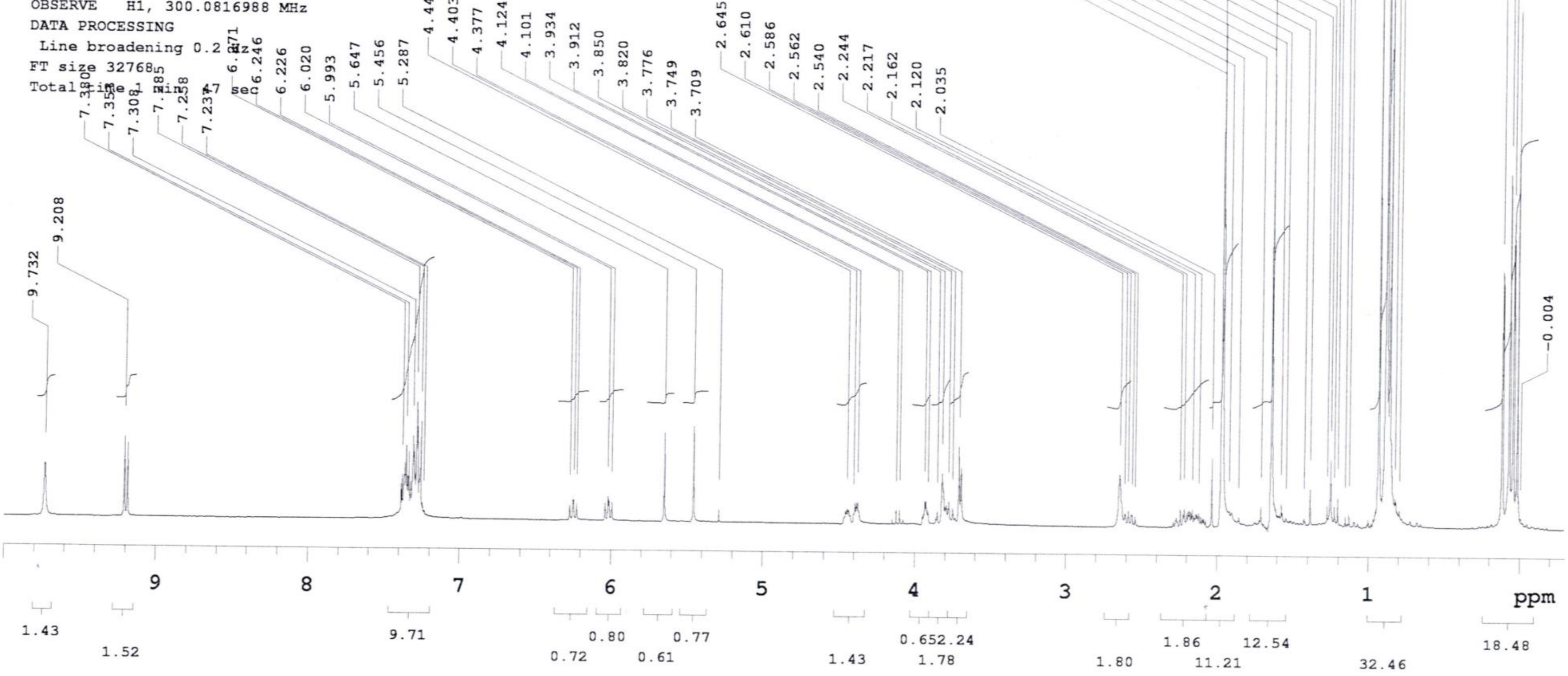

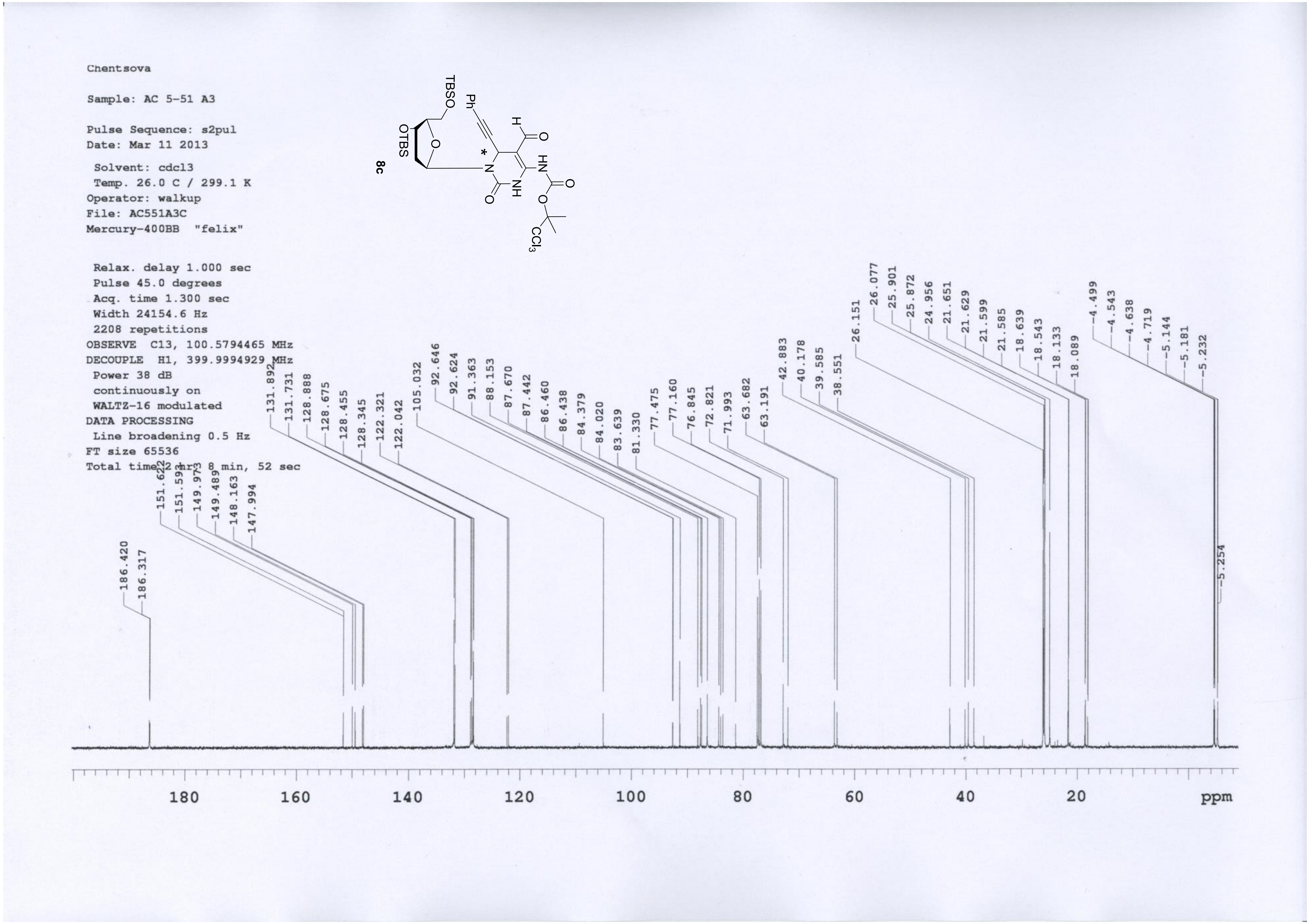
Chentsova
Sample: AC 5-51 A3
Pulse Sequence: s2pul
Date: Mar 11 2013
Solvent: cdcl3
Temp. 26.0 C / 299.1 K
Operator: walkup
File: AC551A3C
Mercury-400BB "felix"
Relax. delay 1.000 sec
Pulse 45.0 degrees
Acq. time 1.300 sec
Width 24154.6 Hz
2208 repetitions
OBSERVE C13, 100.5794465 MHz
DECOUPLE H1, 399.9994929 MHz
Power 38 dB
continuously on
WALTZ-16 modulated
DATA PROCESSING
Line broadening 0.5 Hz
FT size 65536
Total time 2 hr, 8 min, 52 sec
TBSO
OTBS
Ph
H
O
HN
O
O
CCl3
NH
N
O
*
8c
186.420
186.317
151.622
151.593
149.973
149.489
148.163
147.994
131.892
131.731
128.888
128.675
128.455
128.345
122.321
122.042
105.032
92.646
92.624
91.363
88.153
87.670
87.442
86.460
86.438
84.379
84.020
83.639
81.330
77.475
77.160
76.845
72.821
71.993
63.682
63.191
42.883
40.178
39.585
38.551
26.151
26.077
25.901
25.872
24.956
21.651
21.629
21.599
21.585
18.639
18.543
18.133
18.089
-4.499
-4.543
-4.638
-4.719
-5.144
-5.181
-5.232
-5.254
180
160
140
120
100
80
60
40
20
ppm

Chentsova

Sample: AC 5-41A12

Pulse Sequence: s2pul
Date: Apr 3 2013

Solvent: cdcl3
Temp. 26.0 C / 299.1 K
Operator: walkup
Mercury-400BB "Felix"

Relax. delay 1.000 sec
Pulse 29.7 degrees
Acq. time 1.996 sec
Width 6398.0 Hz
28 repetitions
OBSERVE H1, 399.9975363 MHz
DATA PROCESSING
Line broadening 0.2 Hz
FT size 32768
Total time 1 min, 50 sec

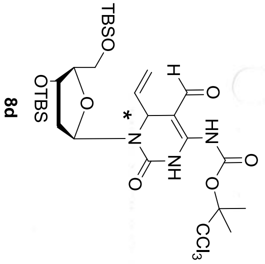

8d

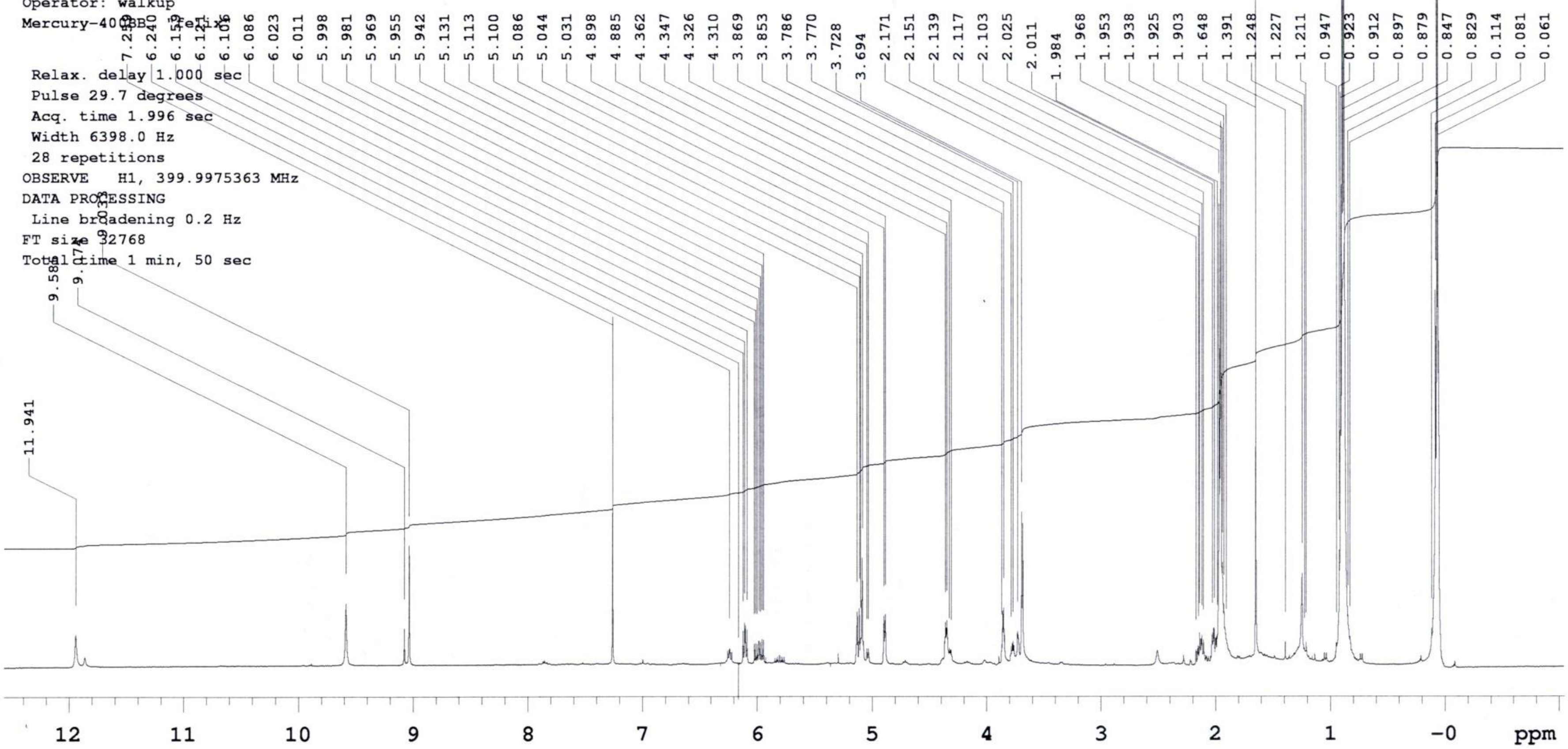

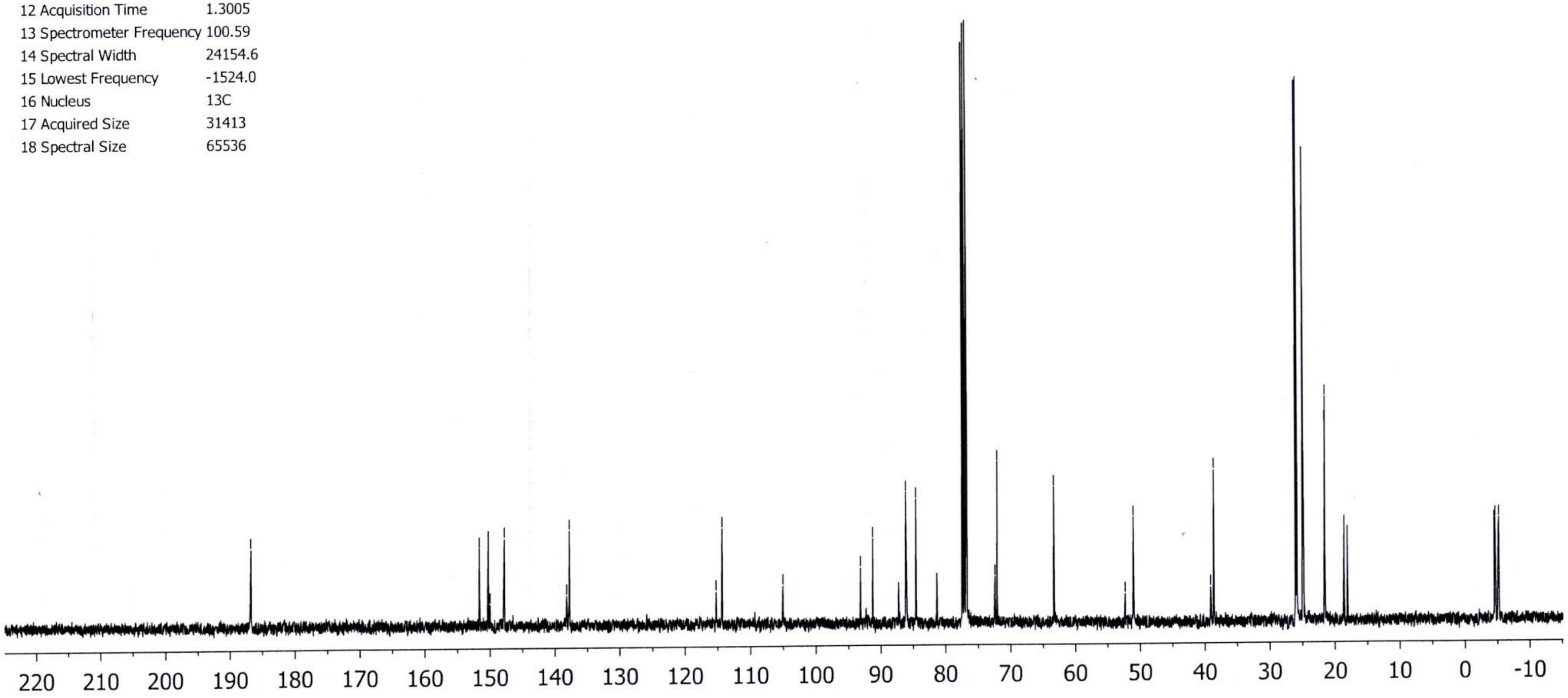

	Parameter	Value
1	Origin	Varian
2	Spectrometer	mercury
3	Author	walkup
4	Solvent	cdcl3
5	Temperature	26.0
6	Pulse Sequence	s2pul
7	Experiment	1D
8	Number of Scans	1376
9	Receiver Gain	20
10	Relaxation Delay	1.0000
11	Pulse Width	0.0000
12	Acquisition Time	1.3005
13	Spectrometer Frequency	100.59
14	Spectral Width	24154.6
15	Lowest Frequency	-1524.0
16	Nucleus	13C
17	Acquired Size	31413
18	Spectral Size	65536

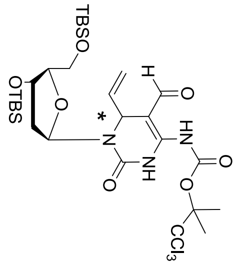

8d

A. Chentsova

Sample: AC5-41A4

Pulse Sequence: s2pul
Date: Feb 18 2013

Solvent: cdcl3
Temp. 26.0 C / 299.1 K
Operator: walkup
Mercury-400BB "felix"

Relax. delay 1.000 sec
Pulse 29.7 degrees
Acq. time 1.996 sec
Width 6398.0 Hz
32 repetitions
OBSERVE H1, 399.9975357 MHz
DATA PROCESSING
Line broadening 0.2 Hz
FT size 32768
Total time 1 min, 50 sec

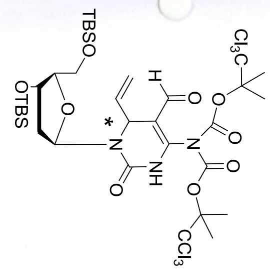

8e

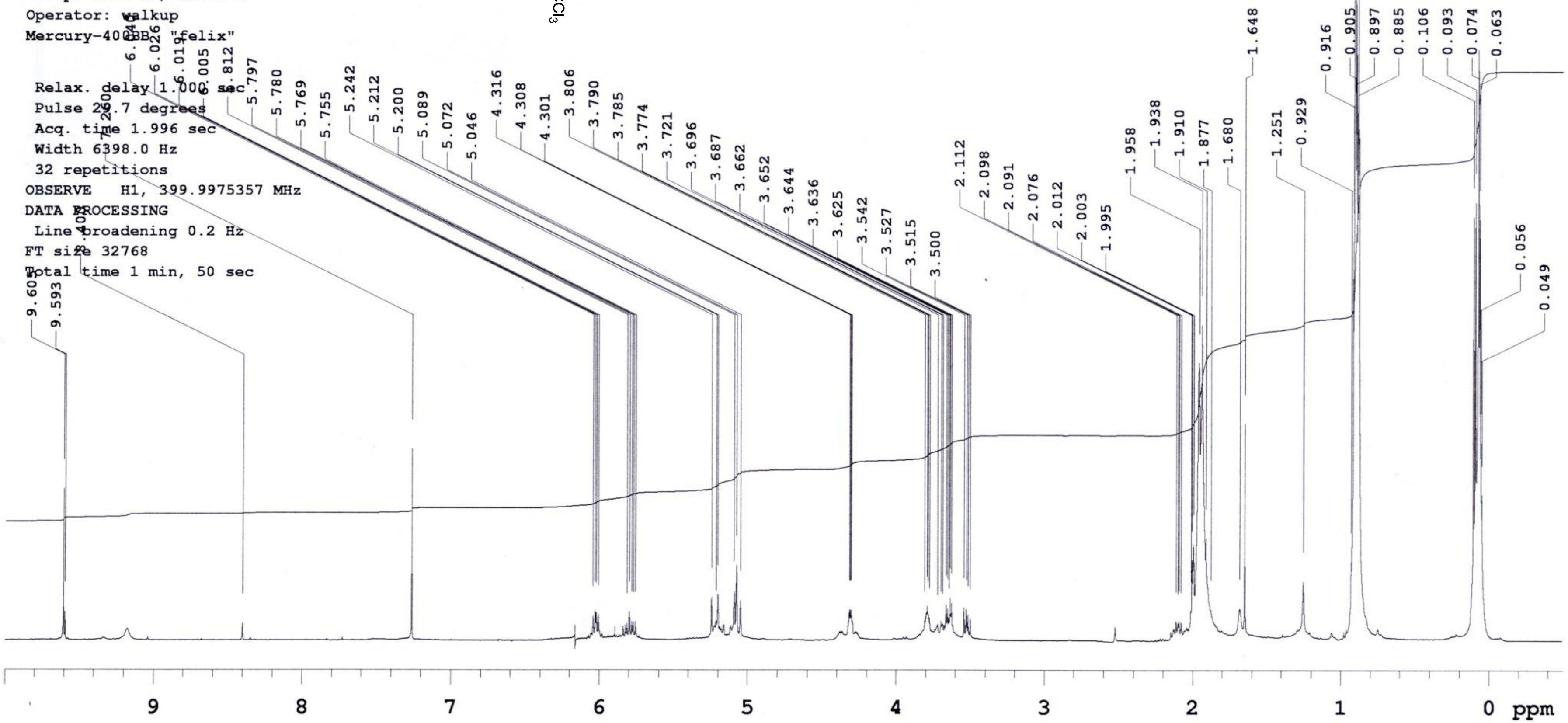

A. Chentsova

Sample: AC5-41A4

Pulse Sequence: s2pul

Solvent: cdcl3
Temp. 26.0 C / 299.1 K
Operator: vnmr1
Mercury-300BB "jutta"

Relax. delay 1.000 sec
Pulse 45.0 degrees
Acq. time 1.301 sec
Width 18115.9 Hz
1872 repetitions
OBSERVE C13, 75.4555905 MHz
DECOUPLE H1, 300.0831467 MHz
Power 37 dB
continuously on
WALTZ-16 modulated
DATA PROCESSING
Line broadening 0.5 Hz
FT size 65536
Total time 1 hr, 24 min, 1 sec

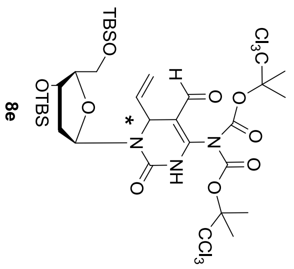

8e

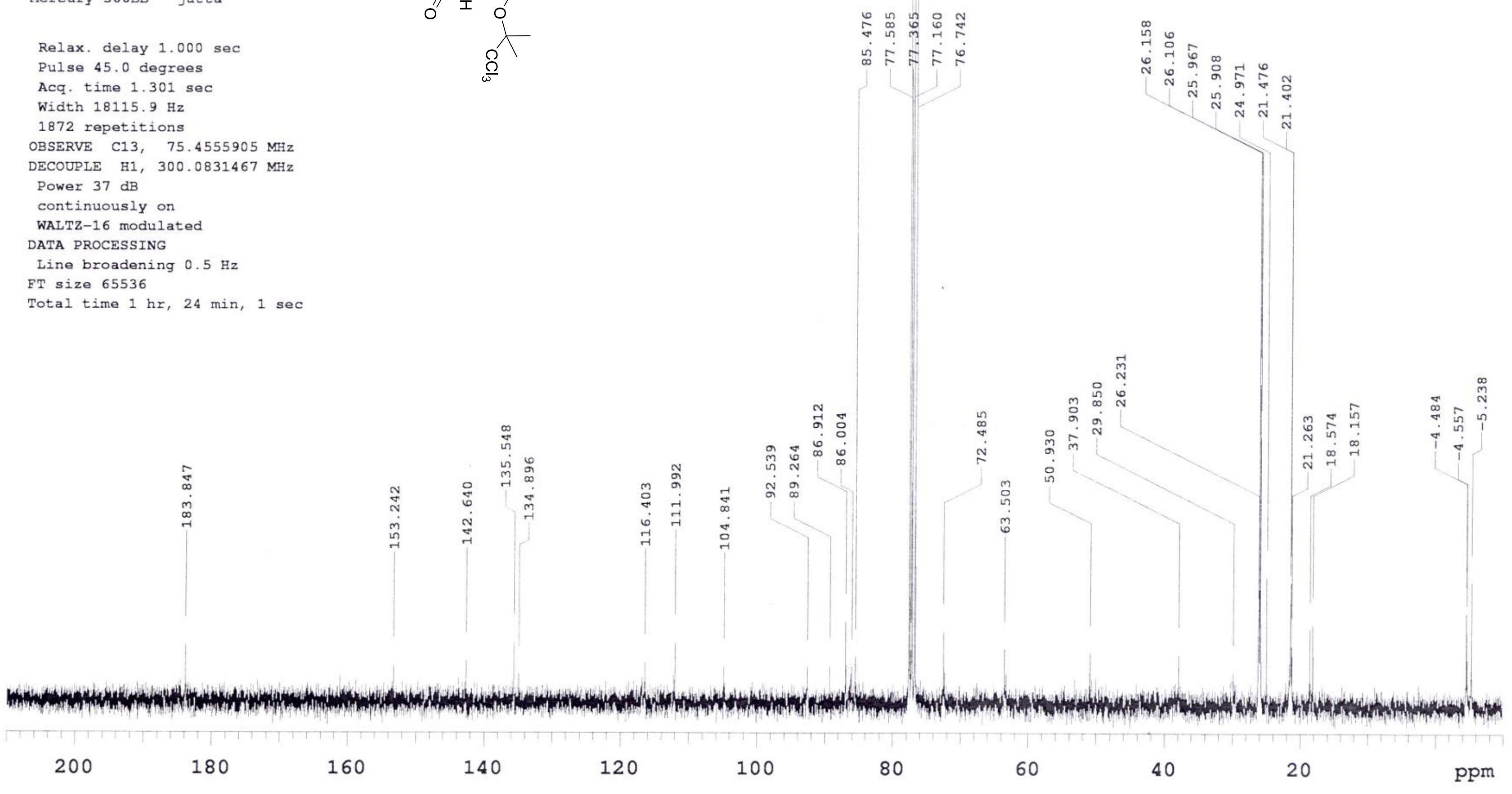

A. Chentsova

Sample: AC5-57A1

Pulse Sequence: s2pul

Solvent: cdcl3
Temp. 26.0 C / 299.1 K
Operator: vnmr1
Mercury-300BB "jutta"

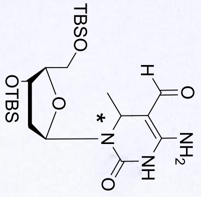

9a

Relax. delay 1.000 sec
Pulse 45.0 degrees
Acq. time 1.998 sec
Width 4800.8 Hz
16 repetitions
OBSERVE H1, 300.0816988 MHz
DATA PROCESSING
FT size 32768
Total time 1 min, 47 sec

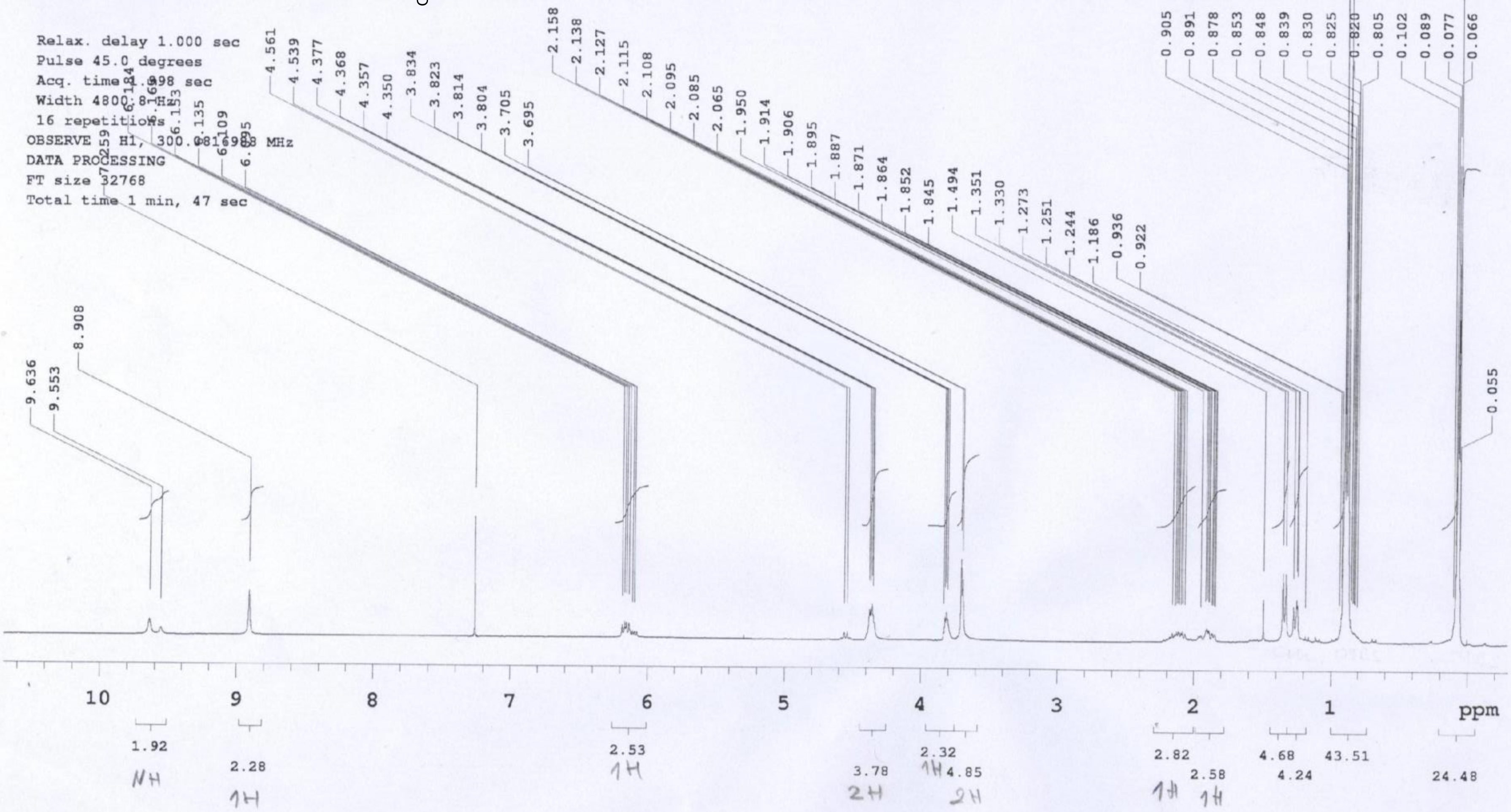

Chentsova

Sample: AC 5-57A1

Pulse Sequence: s2pul
Date: Apr 4 2013

Solvent: cdcl3
Temp. 26.0 C / 299.1 K
Operator: walkup
Mercury-400BB "felix"

Relax. delay 1.000 sec
Pulse 45.0 degrees
Acq. time 1.300 sec
Width 24154.6 Hz
736 repetitions
OBSERVE C13, 100.5794465 MHz
DECOUPLE H1, 399.9994929 MHz
Power 38 dB
continuously on
WALTZ-16 modulated
DATA PROCESSING
Line broadening 0.5 Hz
FT size 65536
Total time 42 min, 58 sec

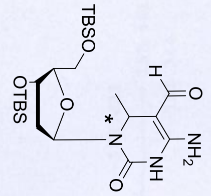

9a

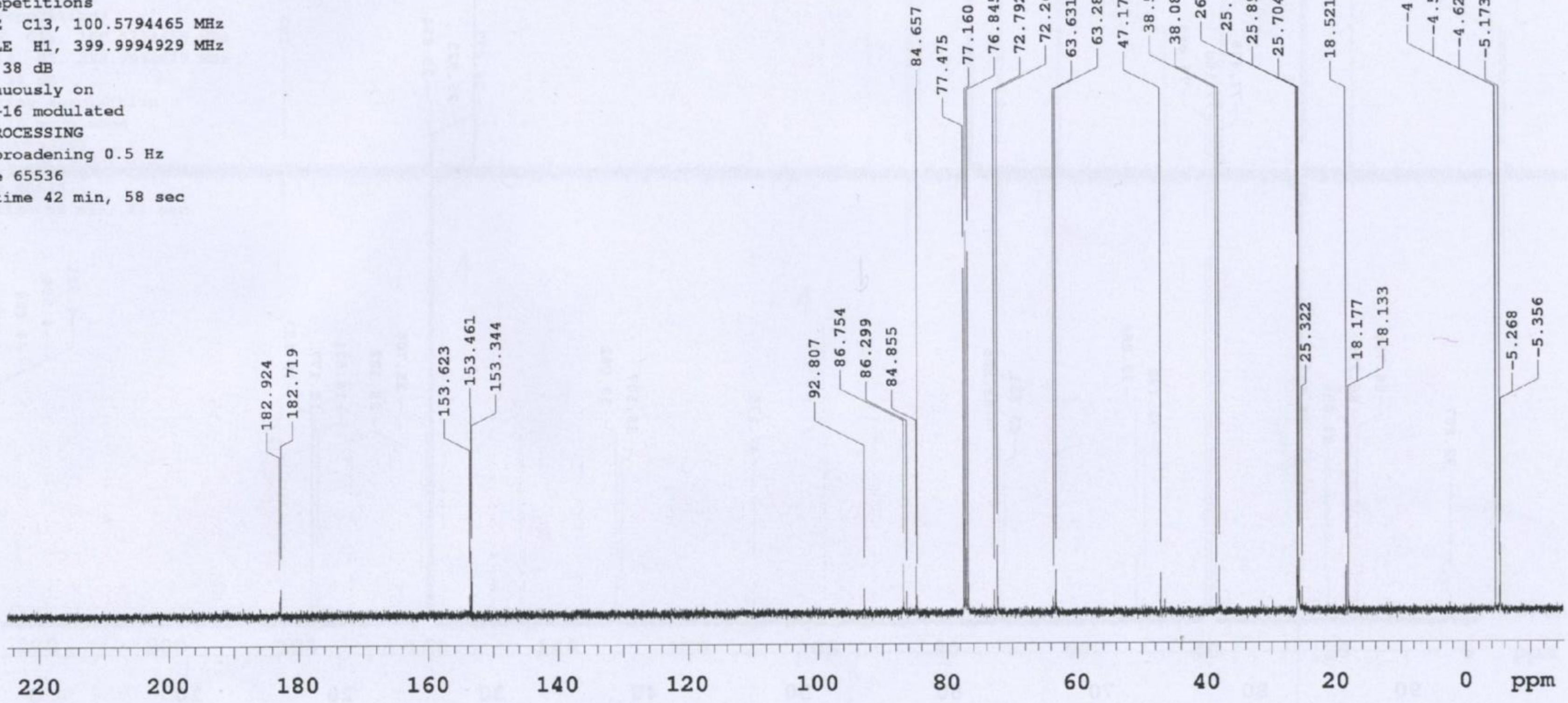

A. Chentsova

Sample: AC5-58

Pulse Sequence: s2pul
Date: Apr 11 2013

Solvent: cdcl3
Temp. 26.0 C / 299.1 K
Operator: walkup
Mercury-400BB "felix"

Relax. delay 1.000 sec
Pulse 29.7 degrees
Acq. time 1.996 sec
Width 7199.4 Hz
16 repetitions
OBSERVE H1, 399.9975363 MHz
DATA PROCESSING
Line broadening 0.2 Hz
FT size 32768
Total time 1 min, 52 sec

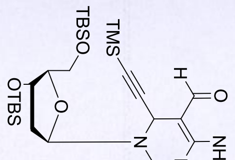

9b

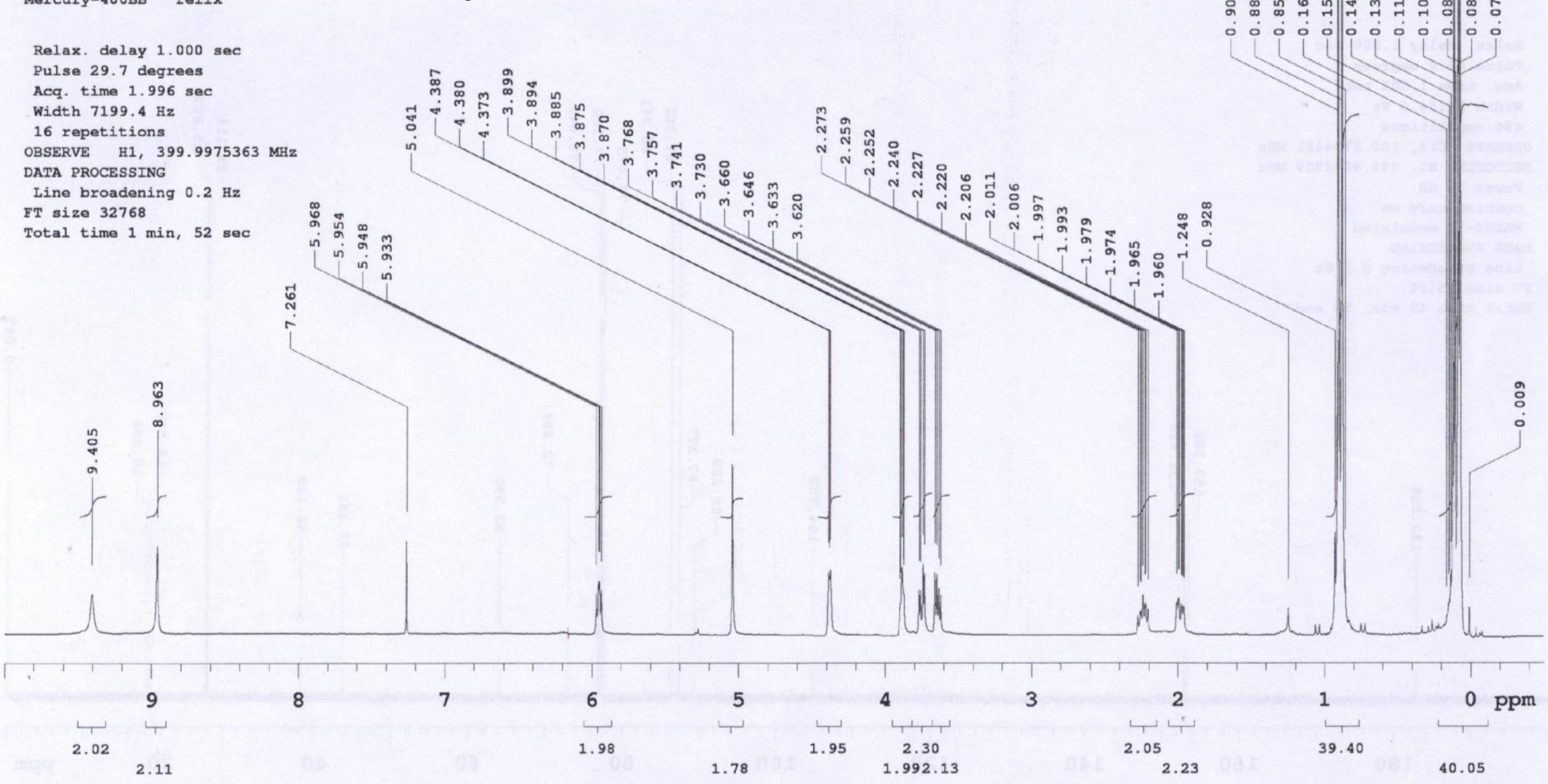

A. Chentsova

Sample: AC5-58

Pulse Sequence: s2pul
Date: Apr 11 2013

Solvent: cdcl3
Temp. 26.0 C / 299.1 K
Operator: walkup
File: AC558C
Mercury-400BB "felix"

Relax. delay 1.000 sec
Pulse 45.0 degrees
Acq. time 1.300 sec
Width 24154.6 Hz
496 repetitions
OBSERVE C13, 100.5794451 MHz
DECOUPLE H1, 399.9994929 MHz
Power 38 dB
continuously on
WALTZ-16 modulated
DATA PROCESSING
Line broadening 0.5 Hz
FT size 65536
Total time 42 min, 58 sec

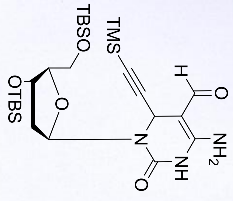

9b

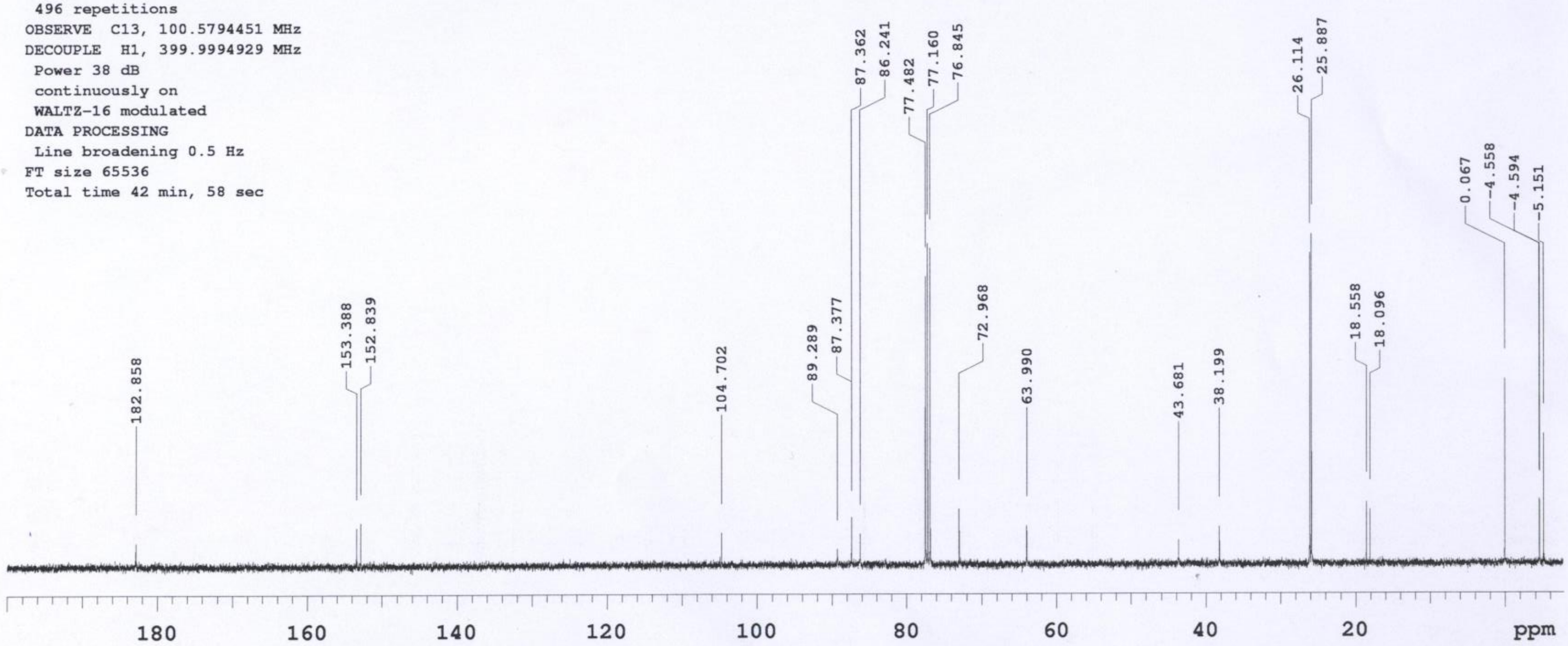

Std Proton parameters
A. Chentsova

Sample: AC5-56

Pulse Sequence: s2pul
Solvent: cdcl3
Temp. 26.0 C / 299.1 K
Operator: vnmr1
Mercury-300BB "jutta"

Relax. delay 1.000 sec
Pulse 45.0 degrees
Acq. time 2.000 sec
Width 4800.8 Hz
16 repetitions
OBSERVE H1, 300.0816981 MHz
DATA PROCESSING
Line broadening 0.2 Hz
FT size 32768
Total time 1 min, 11 sec

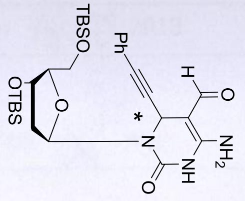

9c

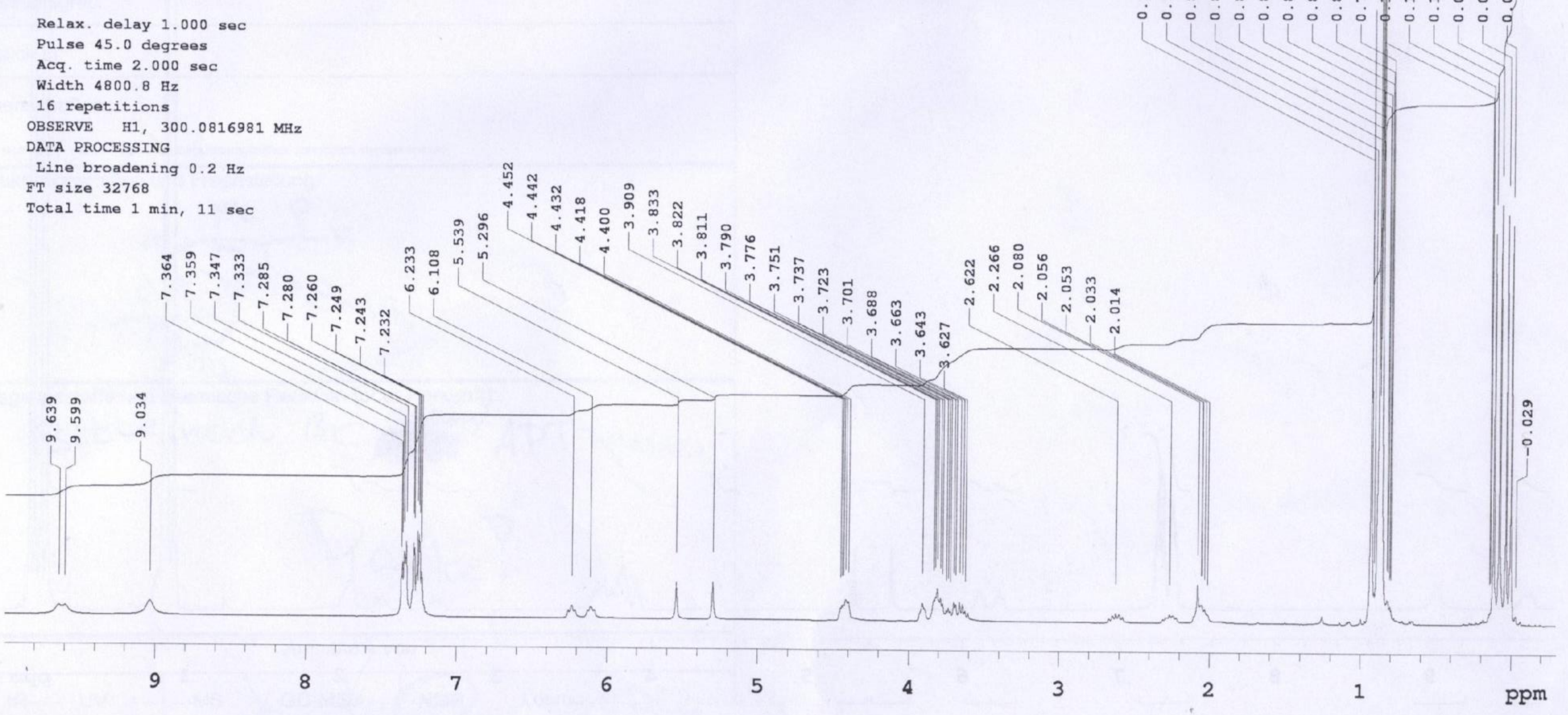

A. Chentsova

Sample: AC5-56

Pulse Sequence: s2pul
Date: Mar 14 2013

Solvent: cdcl3
Temp. 26.0 C / 299.1 K
Operator: walkup
Mercury-400BB "felix"

Relax. delay 1.000 sec
Pulse 45.0 degrees
Acq. time 1.300 sec
Width 24154.6 Hz
10000 repetitions
OBSERVE C13, 100.5794458 MHz
DECOUPLE H1, 399.9994929 MHz
Power 38 dB
continuously on
WALTZ-16 modulated
DATA PROCESSING
Line broadening 1.0 Hz
FT size 65536
Total time 6 hr, 49 min, 3 sec

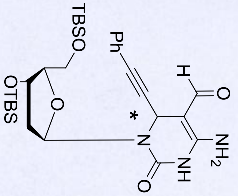

9c

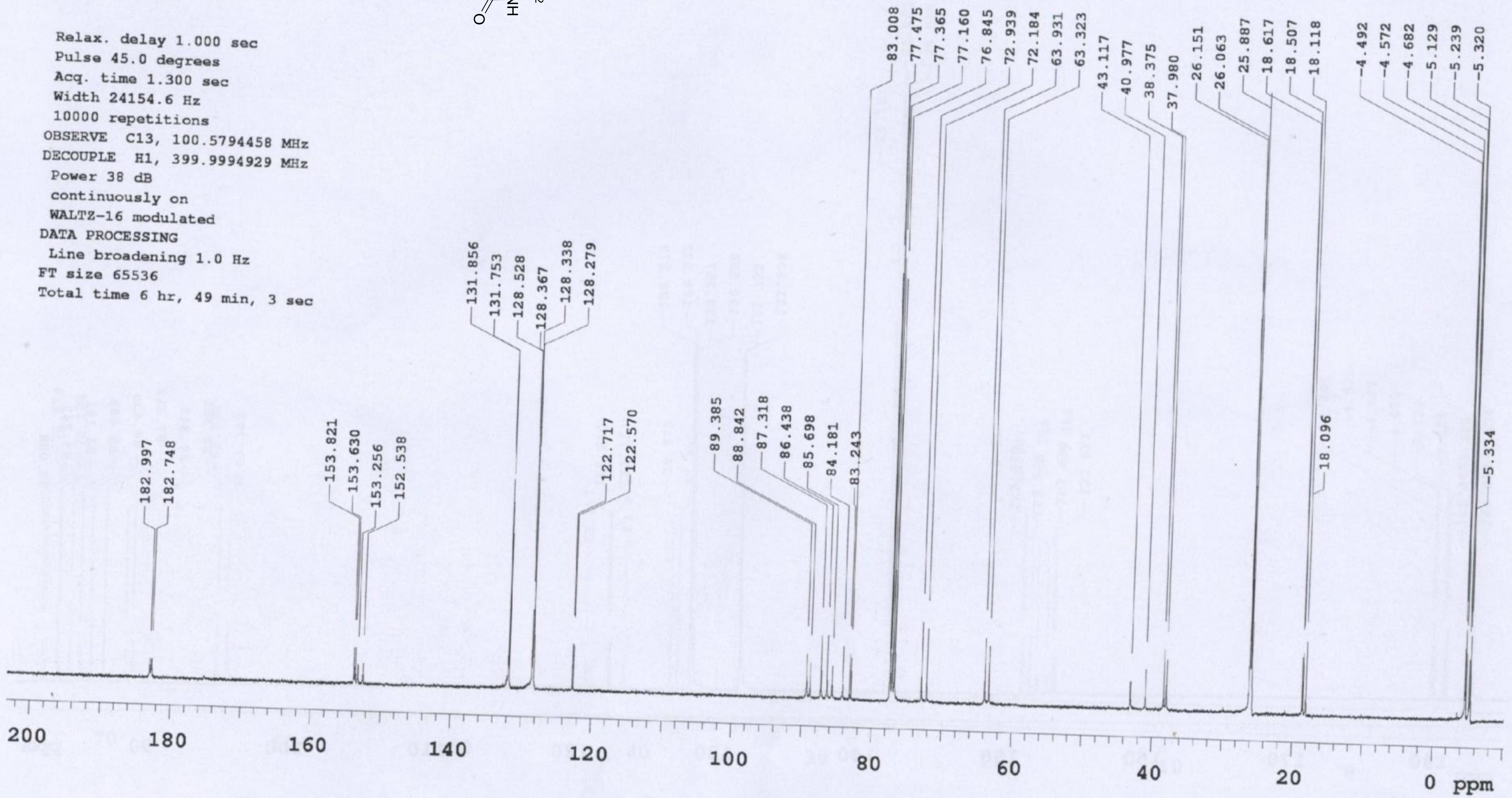

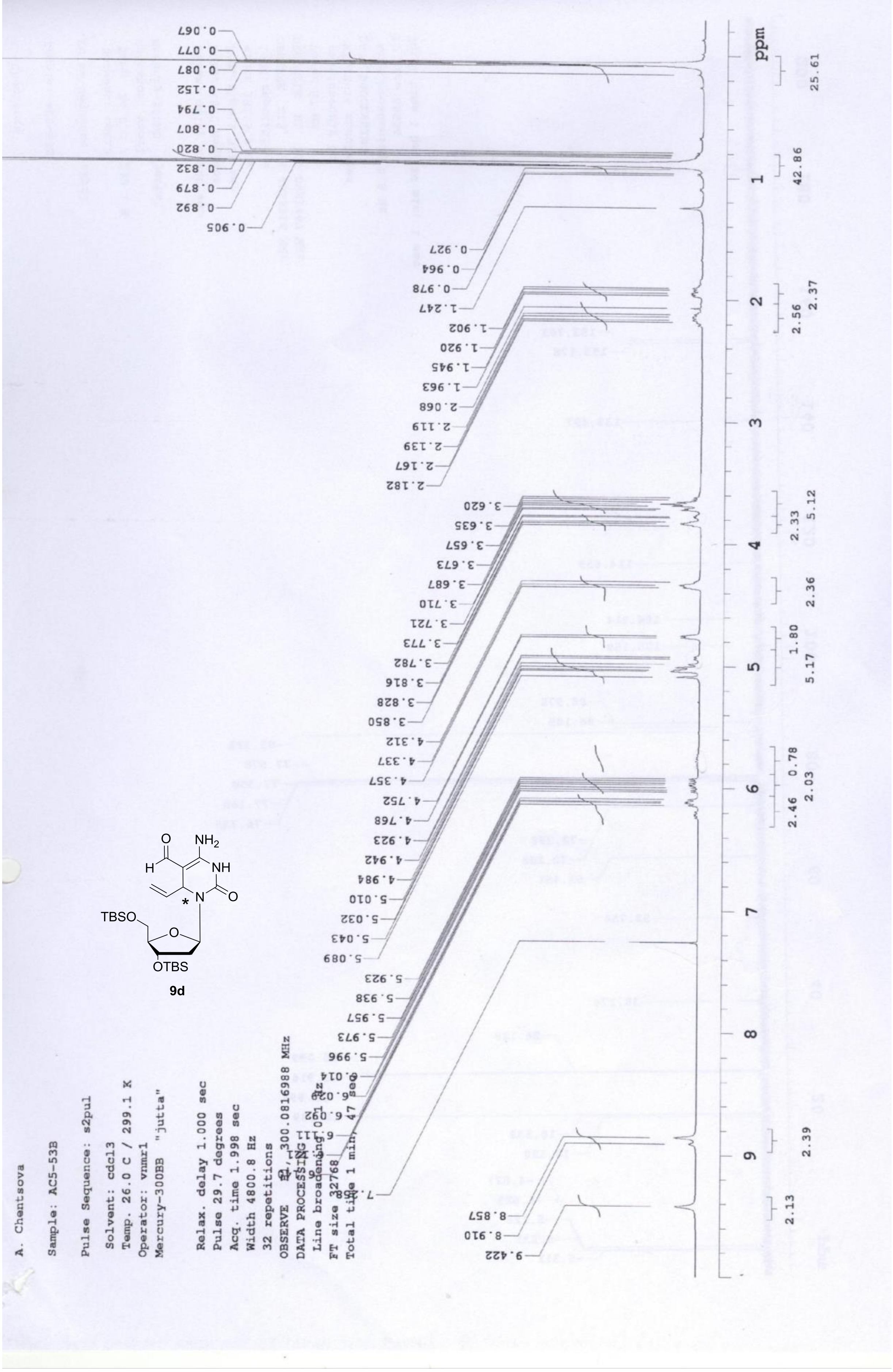
A. Chentsova
Sample: AC5-53B
Pulse Sequence: s2pul
Solvent: cdcl3
Temp. 26.0 C / 299.1 K
Operator: vnmr1
Mercury-300BB "jutta"
Relax. delay 1.000 sec
Pulse 29.7 degrees
Acq. time 1.998 sec
Width 4800.8 Hz
32 repetitions
DATA PROCESSING
FT size 32768
TBSO
OTBS
NH
O
H
9d
ppm

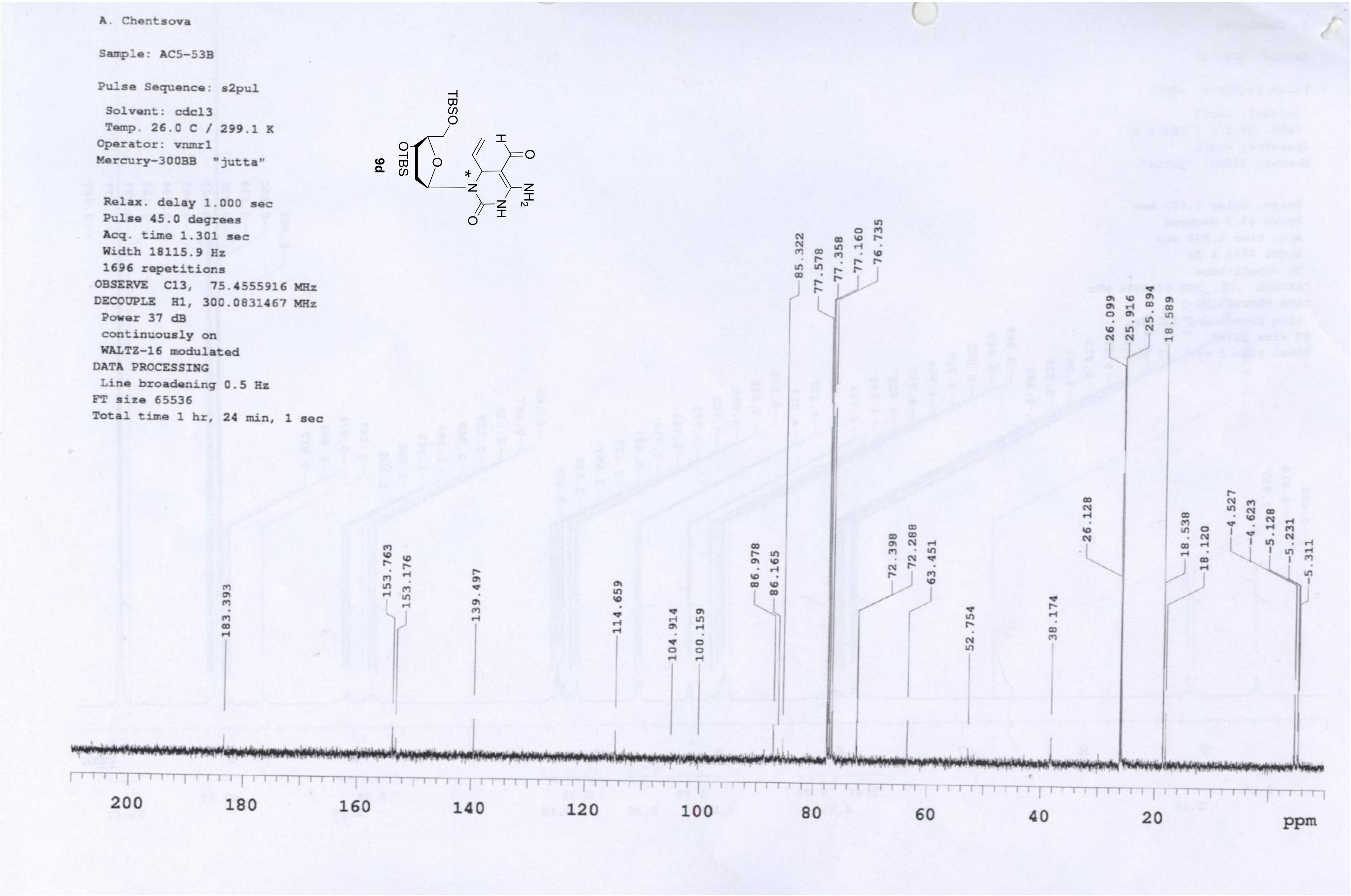
A. Chentsova
Sample: AC5-53B
Pulse Sequence: s2pul
Solvent: cdcl3
Temp. 26.0 C / 299.1 K
Operator: vnmr1
Mercury-300BB "jutta"
Relax. delay 1.000 sec
Pulse 45.0 degrees
Acq. time 1.301 sec
Width 18115.9 Hz
1696 repetitions
OBSERVE C13, 75.4555916 MHz
DECOUPLE H1, 300.0831467 MHz
Power 37 dB
continuously on
WALTZ-16 modulated
DATA PROCESSING
Line broadening 0.5 Hz
FT size 65536
Total time 1 hr, 24 min, 1 sec
TBSO
OTBS
O
H
NH2
NH
N
*
9d
183.393
153.763
153.176
139.497
114.659
104.914
100.159
86.978
86.165
85.322
77.578
77.358
77.160
76.735
72.398
72.288
63.451
52.754
38.174
26.128
26.099
25.916
25.894
18.589
18.538
18.120
-4.527
-4.623
-5.128
-5.231
-5.311
200
180
160
140
120
100
80
60
40
20
ppm
